# Comparative efficacy and safety of tislelizumab and other programmed cell death protein 1 inhibitors in first-line treatment of advanced gastroesophageal cancers: a systematic review and network meta-analysis

**DOI:** 10.1007/s10120-025-01660-4

**Published:** 2025-10-04

**Authors:** Jaffer A. Ajani, Maria Alsina, Markus Moehler, Keun-Wook Lee, Wenxi Tang, Jason Steenkamp, Emily Prentiss, Kaijun Wang, Becky Hooper, Lin Zhan

**Affiliations:** 1 Division of Cancer Medicine, Department of Gastrointestinal Medical Oncology, The University of Texas MD Anderson Cancer Center, Houston, TX USA; 2 Medical Oncology Department, Unidad de Oncología Médica Traslacional, Hospital Universitario de Navarra, Navarrabiomed, IdiSNA, Pamplona, Spain; 3 Gastrointestinal Oncology, Johannes Gutenberg-University Clinic, Mainz, Germany; 4 Department of Internal Medicine, Seoul National University College of Medicine, Seoul National University Bundang Hospital, Seongnam, Republic of Korea; 5 BeOne Medicines, Ltd., San Carlos, CA USA; 6 Value & Evidence Services, EVERSANA, Burlington, ON Canada

**Keywords:** Stomach neoplasms, Immune checkpoint inhibitors, Programmed cell death protein-1, Network meta-analysis

## Abstract

**Background:**

Several programmed cell death protein-1 inhibitors are approved for the first-line treatment of advanced gastric/gastroesophageal junction cancer, including pembrolizumab, nivolumab and, more recently, tislelizumab. Since direct comparisons between these agents are lacking, advanced statistical modeling can be utilized to evaluate the relative efficacy and safety of tislelizumab compared with other first-line immunotherapy regimens in this indication.

**Methods:**

A systematic literature review was performed to identify and summarize published randomized controlled trials investigating first-line treatments in adult patients with unresectable, locally advanced, or metastatic human epidermal growth factor receptor 2-negative gastric/gastroesophageal junction cancer. Relevant trials were synthesized using a Bayesian network meta-analysis; fixed-effect models were conducted for all analyses. The network meta-analysis base case used the intent-to-treat populations for tislelizumab + chemotherapy and placebo + chemotherapy from RATIONALE-305.

**Results:**

Key comparators included nivolumab + chemotherapy (ATTRACTION-4, CheckMate 649), and pembrolizumab + chemotherapy (KEYNOTE-062, KEYNOTE-859). Tislelizumab + chemotherapy demonstrated similar efficacy compared with nivolumab + chemotherapy and pembrolizumab + chemotherapy for both overall survival and progression-free survival. Tislelizumab + chemotherapy was associated with significantly lower odds of grade ≥ 3 treatment-related adverse events compared with nivolumab + chemotherapy, and there were no statistically significant differences between tislelizumab + chemotherapy compared with pembrolizumab + chemotherapy.

**Conclusion:**

Overall, these analyses suggest that tislelizumab + chemotherapy is similarly efficacious to pembrolizumab + chemotherapy and nivolumab + chemotherapy, and is associated with a similar or lower incidence of grade ≥ 3 treatment-related adverse events in the first-line treatment of gastric/gastroesophageal junction cancer.

**Supplementary Information:**

The online version contains supplementary material available at 10.1007/s10120-025-01660-4.

## Introduction

Gastric cancer (GC) imposes a significant global burden as the fourth most common cancer type and accounting for 8% of all cancer-related deaths [1, 2]. In 2020, there were an estimated 1.1 million incident cases of GC and 770,000 GC-related deaths globally [1]. Approximately 90–95% of GC cases are adenocarcinomas, which can be further categorized according to the tumor’s anatomical location, histology, and molecular characteristics [3–6]. The majority of tumors arising at the gastroesophageal junction (GEJ) are also adenocarcinomas and are often grouped together with either GC or esophageal cancer depending on the tumor location [7, 8]. Due to the asymptomatic nature of early-stage GC, most cases are not diagnosed until an advanced stage (particularly in Western countries, which do not have screening programs), resulting in poor outcomes and limited treatment options [9–12]. Historically, first-line (1L) treatments for unresectable, advanced or metastatic gastric cancer/gastroesophageal junction cancer (GC/GEJC) have included platinum-based doublet chemotherapies, although these regimens are associated with limited survival benefit [5–7, 13, 14]. Programmed cell death protein-1 (PD-1) inhibitors are immune checkpoint inhibitors (ICIs) that have emerged as promising treatment options in a range of cancer types, including unresectable, advanced or metastatic GC/GEJC [15].

The PD-1 inhibitors pembrolizumab and nivolumab, in combination with fluoropyrimidine- and platinum-containing chemotherapy (CT), have received broad regulatory approval for the 1L treatment of adults with unresectable, advanced or metastatic human epidermal growth factor receptor 2 (HER2)-negative GC/GEJC, including in the United States (US) and European Union (EU), and are recommended as treatment options in clinical practice guidelines for patients whose tumors express programmed cell death-ligand 1 (PD-L1) [16–21]. In the EU, pembrolizumab in combination with CT is indicated in patients with GC/GEJC whose tumors express PD-L1 with a combined positive score (CPS) of ≥ 1, and nivolumab in combination with CT is indicated in patients whose tumors express PD-L1 with a CPS of ≥ 5 [18, 19]. In the US, pembrolizumab and nivolumab (each in combination with CT) are approved for patients with GC/GEJC whose tumors express PD-L1 (CPS ≥ 1 for pembrolizumab; PD-L1 ≥ 1 for nivolumab) [16, 17].

Tislelizumab is a humanized immunoglobulin G4 (IgG4) anti-PD-1 monoclonal antibody recently approved for the treatment of HER2-negative GC/GEJC (in combination with platinum and fluoropyrimidine-based chemotherapy) in the US (in patients whose tumors express PD-L1 [≥ 1]) and EU (in patients whose tumors express PD-L1 Tumor Area Positivity [TAP] score ≥ 5%) [22, 23]. The efficacy and safety of tislelizumab + CT as a 1L treatment in patients with unresectable, locally advanced recurrent or metastatic gastric or GEJ adenocarcinoma was evaluated in the Phase 3, double-blind, placebo-controlled RATIONALE-305 trial (NCT03777657) [24]. Tislelizumab + CT was found to provide more favorable overall survival (OS) versus placebo + CT in patients whose tumors express PD-L1 and in all randomized patients [24–26]. However, no studies to date have directly compared tislelizumab with other relevant 1L immunotherapy treatments for GC/GEJC.

The objective of this study was to conduct a network meta-analysis (NMA) to evaluate the relative efficacy and safety of tislelizumab + CT compared with other broadly approved immunotherapy regimens for the 1L treatment of patients with unresectable, locally advanced, or metastatic GC/GEJC.

## Methods

### Systematic literature review

To identify relevant studies, a systematic literature review (SLR) was performed to find and summarize published randomized controlled trials (RCTs) investigating 1L treatments in adult patients with unresectable, locally advanced, or metastatic HER2-negative GC/GEJC. The review was conducted in accordance with the Cochrane Handbook for Systematic Reviews of Interventions and reported in alignment as per the Preferred Reporting Items for Systematic Literature Reviews and Meta-Analyses (PRISMA) statement [27–29]. The Population, Intervention, Comparator, Outcome, Study design (PICOS) framework was used to develop the search strategy and structure the reporting of the eligibility criteria. The search strategy was created and performed by a medical information specialist in collaboration with the review team (see details in Online Resource 1). The search was conducted in February 2024 using the Ovid® platform to search the following electronic databases: Embase, Ovid MEDLINE® (including Epub Ahead of Print and In-Process & Other Non-Indexed Citations), Ovid MEDLINE® Daily, Cochrane Central Register of Controlled Trials, and the Cochrane Database of Systematic Reviews. The searches were performed from database inception to February 16, 2024.

Additional supplemental searches of relevant conference proceedings, health technology assessments, and trial registries were also conducted to maximize the inclusion of relevant studies. Study selection was conducted by two reviewers who independently assessed eligibility based on the pre-defined criteria. Discrepancies were resolved by consensus or a third independent reviewer. Data extraction and quality assessment were performed for studies meeting all inclusion criteria by a single reviewer and confirmed by a second reviewer. A standardized data extraction form in Microsoft® Excel (Microsoft Corporation, Seattle, WA, US) was used. Details of the above are provided in Online Resource 1.

### Network meta-analysis

An NMA feasibility assessment was conducted to evaluate clinical heterogeneity across all relevant trials. Trial design characteristics, patient eligibility criteria, baseline patient characteristics, outcome characteristics (i.e., definitions, methods of reporting outcomes, and follow-up time for safety outcomes) were all sources of clinical heterogeneity explored in the feasibility assessment. Feasibility was confirmed for the following outcomes: OS, progression-free survival (PFS), objective response rate (ORR), and grade ≥ 3 treatment-related adverse events (TRAEs).

Network diagrams were developed to visualize the evidence base for each outcome. To form connected network diagrams, all chemotherapy backbone treatments were assumed to be comparable and were pooled together into a single node [30, 31]. This assumption is aligned with other published NMAs that assessed treatment options in this disease space [32–35].

NMAs were conducted using a Bayesian framework and performed using R version 3.6.1, Just Another Gibbs Sampler (JAGS), and WinBUGS [30]. Point estimates and 95% credible intervals (CrIs) were modeled for outcomes using Markov Chain Monte Carlo methods. The probability that each treatment was the most efficacious regimen (P-best), and the Surface area Under the Cumulative Ranking curve (SUCRA) values were calculated to reflect the probability of an intervention being among the best options [31]. While ranking metrics like SUCRA and P-best estimate the probability that a treatment is among the best, they must be interpreted alongside CrIs, as they do not account for statistical or clinical significance and may be misleading if the effect sizes are small or not statistically significant. When the pairwise comparisons are not significant (i.e., the CrIs overlap), rankings are uncertain and should be interpreted with caution as the differences may be due to chance.

For time-to-event outcomes (OS, PFS), a fixed-effect, contrast-based normal model with identity link function was used with vague priors for treatment effects. Hazard ratios (HRs) and their 95% CrIs were calculated for comparisons between treatments, with the HR and 95% confidence intervals (CIs) extracted from included trials when available. Stratified HRs and 95% CIs were used when available; otherwise, the unstratified HRs and associated 95% CIs were used. As comparing time-to-event outcomes between interventions using HRs requires the proportional hazards (PH) assumption to hold, the PH assumption for OS and PFS was assessed via visual inspection of log-cumulative hazard plots, Schoenfeld residuals plots, and performance of the Grambsch-Therneau test. For binomial outcomes (ORR, grade ≥ 3 TRAEs), a fixed-effect, arm-based binomial model with logit link function was used with vague priors for treatment effects. The odds ratio (OR) and its 95% CrI were calculated. The treatment effects were estimated on the log odds scale and then transformed back to OR for presentation. Observed differences in HRs and ORs were considered statistically significant if the 95% CrI range did not cross 1.

Although random-effects models may have been feasible for the base case intent-to-treat (ITT) scenario networks as these networks were the largest with some multiple study connections, for all other analyses, robust scenario analyses could not be conducted given the frequency of single trial connections. For this reason, fixed-effect models were used for all analyses because they are favored over random-effects models when networks have relatively few studies (i.e., five or fewer) or many single-study connections [36, 37].

### Base case and subgroup analyses

The base case used the randomized, ITT populations for tislelizumab + CT and placebo + CT from RATIONALE-305 (data cut-off: February 28, 2023) [24]. Key comparators included nivolumab + CT (ATTRACTION-4 [data cut-off: October 31, 2018]; Checkmate 649 [data cut-off: May 27, 2021]), and pembrolizumab + CT (KEYNOTE-062 [data cut-off March 26, 2019]; KEYNOTE-859 [data cut-off October 3, 2022]) [38–41]. Baseline characteristics of patients included in these trials are summarized in Online Resource 2. In several instances where specific data were not reported in the publications identified in the SLR, they were retrieved from the US Food and Drug Administration (FDA) Briefing Document on immune checkpoint inhibitors in patients with metastatic or unresectable HER2-negative gastric adenocarcinoma [42].

Subgroup analyses were conducted to assess OS, PFS, and ORR based on PD-L1 status, including PD-L1 ≥ 1 (TAP score  ≥ 1% or CPS ≥ 1) and PD-L1 ≥ 5 (TAP score ≥ 5% or CPS ≥ 5). Although nivolumab and pembrolizumab trials used CPS to measure PD-L1 expression, whereas RATIONALE-305 utilized TAP scores, the two measures were deemed equivalent and interchangeable with respect to clinical utility based on the high concordance between TAP score and CPS demonstrated in RATIONALE-305 [25, 43]. To further confirm this assumption, a sensitivity analysis was conducted that compared OS in the PD-L1 ≥ 5 subgroup using CPS rather than TAP scores for RATIONALE-305. Additional subgroup analyses of efficacy outcomes were conducted where feasible (i.e., contingent on data availability to inform comparisons). These included subgroup analyses of OS and PFS by geographic region (Asia and non-Asia [rest of world; ROW]), and analyses of OS by primary tumor location (stomach or GEJ). Further, to confirm the assumption that the underlying chemotherapy treatments were equivalent, a subgroup analysis of OS was conducted with only platinum plus fluoropyrimidine chemotherapy treatments. No subgroup analyses were performed for grade ≥ 3 TRAEs because of a lack of available data.

## Results

### Study selection

**Fig. 1:**
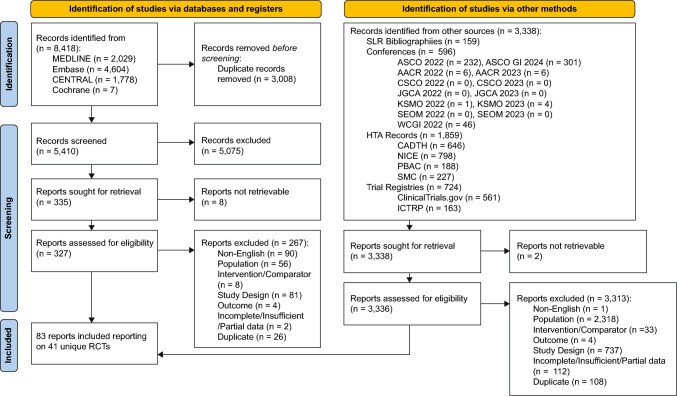
PRISMA flow diagram of clinical evidence. *AACR* American Association for Cancer Research, *ASCO* American Society of Clinical Oncology, *ASCO GI* American Society of Clinical Oncology Gastrointestinal Cancers Symposium, *CADTH* Canadian Agency for Drugs and Technologies in Health, *CSCO* Chinese Society of Clinical Oncology, *HTA* health technology assessment, *ICTRP* International Clinical Trials Registry Platform, *JGCA* Japanese Gastric Cancer Association, *KSMO* Korean Society of Medical Oncology; *NICE* National Institute for Health and Care Excellence, *PBAC* Pharmaceutical Benefits Advisory Committee, *RCT* randomized controlled trial, *SEOM* Sociedad Espanola de Oncologia Medica, *SLR* systematic literature review, *SMC* Scottish Medicines Consortium, *WCGI* World Congress on Gastrointestinal Cancer

A total of 5410 unique records were screened from the database searches after deduplication, with an additional 3336 records identified from additional sources during the supplemental search. Following screening, 83 records reporting on 41 unique RCTs met the eligibility criteria and were included in the feasibility assessment (Fig. 1). The feasibility assessment confirmed the appropriateness of an NMA approach, given the similarity of study populations across eligible trials. Trial design characteristics, patient eligibility criteria, baseline patient characteristics, outcome definitions, and follow-up time for safety were all sources of clinical heterogeneity explored in the feasibility assessment. It was considered feasible to conduct NMAs for all outcomes of interest between RATIONALE-305 and 11 other identified trials. In the PH assumption tests using the RATIONALE-305 data (data cut-off date [DCO]: February 28th, 2023), despite patterns suggestive of violation for OS and PFS between tislelizumab and placebo when assessing cumulative hazard log plots and Schoenfeld residuals plots, the Grambsch-Therneau p-value remained > 0.05 indicating no violation of the PH assumption (see Online Resource 4 for details). After restricting the analysis to immunotherapy agents with global regulatory approval for the 1L treatment of HER2-negative GC/GEJC, in addition to tislelizumab + CT, five trials remained for the base case analysis (see Online Resource 2 for details).

### Network meta-analysis

**Fig. 2:**
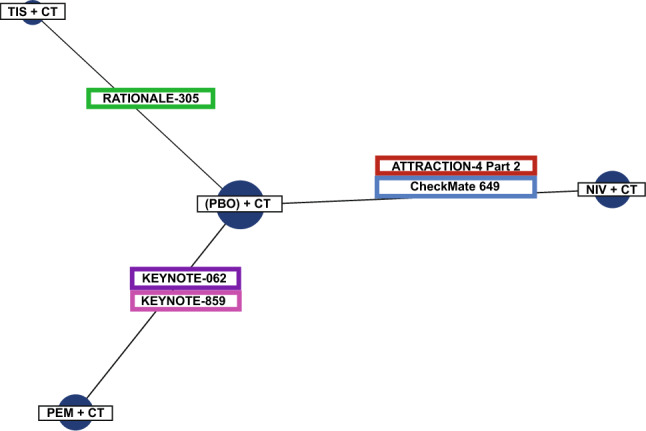
Network diagram for all outcomes. *CT* chemotherapy, *NIV* nivolumab, *PBO* placebo, *PEM* pembrolizumab, *TIS* tislelizumab

The base case network consisted of four treatment nodes informed by five RCTs of PD-1 inhibitors (RATIONALE-305, ATTRACTION-4, CheckMate 649, KEYNOTE-062, and KEYNOTE-859) (Fig. 2) [38–41, 44]. All treatments were anchored to the CT control across trials. The network consisted of 5388 patients for OS and PFS analyses, and 5324 patients for the grade ≥ 3 TRAE analysis.

### Overall survival, progression-free survival, and objective response rate

**Fig. 3:**
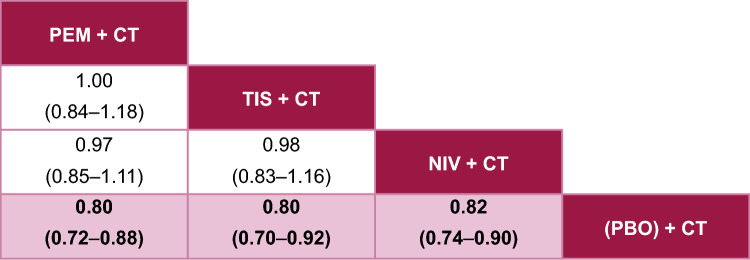
Fixed-effects League Table for OS. Overall population; reported as HR [95% CrI], HR < 1 implies that column is better than row. Pink squares are statistically significant. The treatment with the most favorable estimate is positioned at the top left corner; the second, third, and fourth most favorable treatments are shown in descending order to the lower right. *CrI* credible interval, *CT* chemotherapy, *HR* hazard ratio, *NIV* nivolumab, *OS* overall survival, *PBO* placebo, *PEM* pembrolizumab, *TIS* tislelizumab

**Fig. 4:**
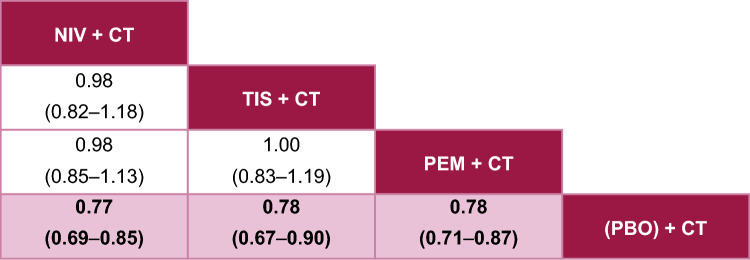
Fixed-effects League Table for PFS. Overall population; reported as HR [95% CrI], HR < 1 implies that column is better than row. Pink squares are statistically significant. The treatment with the most favorable estimate is positioned at the top left corner; the second, third, and fourth most favorable treatments are shown in descending order to the lower right. *CrI* credible interval, *CT* chemotherapy, *HR* hazard ratio, *NIV* nivolumab, *PBO* placebo, *PEM* pembrolizumab, *PFS* progression-free survival, *TIS* tislelizumab

**Fig. 5:**
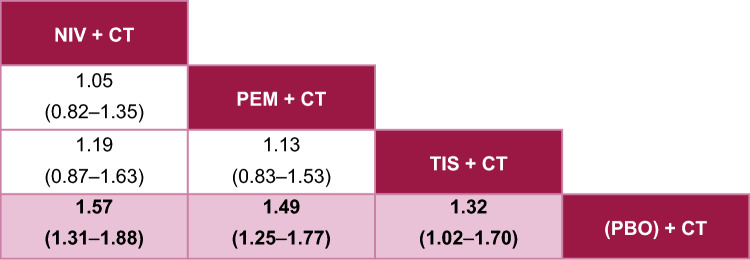
Fixed-effects League Table for ORR. Overall population; reported as OR [95% CrI], OR > 1 implies that column is better than row. Pink squares are statistically significant. The treatment with the most favorable estimate is positioned at the top left corner; the second, third, and fourth most favorable treatments are shown in descending order to the lower right. *CrI* credible interval, *CT* chemotherapy, *NIV* nivolumab, *NMA* network meta-analysis, *OR* odds ratio, *ORR* objective response rate, *PBO* placebo, *PEM* pembrolizumab, *TIS* tislelizumab

In pairwise comparisons, all active treatments were statistically more favorable than the CT control for OS (Fig. 3), PFS (Fig. 4), and ORR (Fig. 5).

For OS, tislelizumab + CT performed similarly to both pembrolizumab + CT and nivolumab + CT, with no significant differences observed (Fig. 3). Pembrolizumab + CT and nivolumab + CT also performed similarly with no significant differences observed. Tislelizumab + CT was associated with the second highest probability of being the top-ranked therapy (SUCRA 69%; P-best 40%), following pembrolizumab + CT (SUCRA 72%; P-best 40%) (see Online Resource 3).

With respect to PFS, tislelizumab + CT performed similarly to both nivolumab + CT and pembrolizumab + CT, with no significant differences observed (Fig. 4). There were also no significant differences between pembrolizumab + CT and nivolumab + CT. Tislelizumab + CT was associated with the second highest SUCRA value of 64% and P-best score of 33%, following nivolumab + CT (see Online Resource 3 for details).

Likewise, for ORR, tislelizumab + CT performed similarly to both nivolumab + CT and pembrolizumab + CT, with no significant differences observed (Fig. 5). Pembrolizumab + CT and nivolumab + CT also performed similarly with no significant differences observed. Tislelizumab + CT was associated with a SUCRA value of 44% and P-best score of 9%, behind pembrolizumab + CT (SUCRA 71%; P-best 31%) and nivolumab + CT (SUCRA 84%; P-best 60%) (see Online Resource 3).

**Fig. 6:**
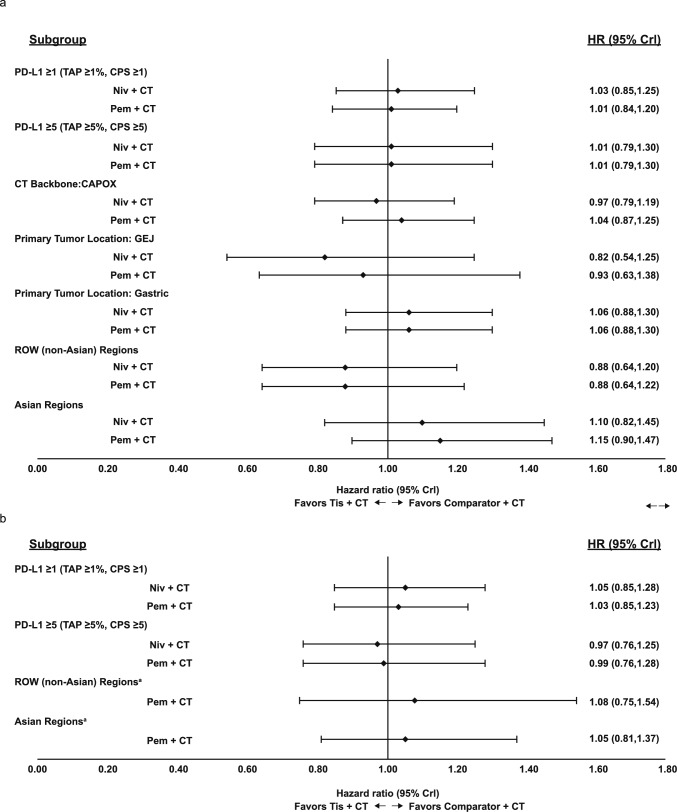
Forest Plots of Subgroup Analyses for OS (**a**) and PFS (**b**), Fixed-effects NMA. An HR > 1 indicates TIS + CT has a greater HR than the comparator therapy. An HR < 1 indicates TIS + CT has a lesser HR than the comparator therapy. For subgroup analyses by PD-L1 status, RATIONALE-305 data are reflective of the TAP scoring method, CheckMate 649, KEYNOTE-062, and KEYNOTE-859 data are reflective of the CPS method, and ATTRACTION-4 part 2 methods are not specified. ^a^Comparison vs. nivolumab + CT was not conducted due to lack of data. *CAPOX* capecitabine and oxaliplatin, *CPS* combined positive score, *CrI* credible interval, *CT* chemotherapy, *GEJ* gastroesophageal junction, *HR* hazard ratio, *ITT* intention-to-treat, *Niv* nivolumab, *OS* overall survival, *Pem* pembrolizumab, *PD-L1* programmed death-ligand 1, *ROW* rest of the world, *TAP* Tumor Area Positivity

Subgroup analyses by PD-L1 status, geographic region, primary tumor location, and chemotherapy backbone subgroups were consistent with the base case analysis for OS and PFS, with no significant differences observed between tislelizumab + CT and either immunotherapy comparator (Fig. 6). Results of a sensitivity analysis comparing OS in the PD-L1 ≥ 5 population using CPS rather than TAP scores for RATIONALE-305 were consistent with the above subgroup analysis in this population (see Online Resource 3). For ORR subgroup analyses of patients with PD-L1CPS  ≥ 5, the results were consistent with base case analysis, with no significant differences between active treatments (see Online Resource 3).

### Grade ≥ 3 TRAEs

**Fig. 7:**
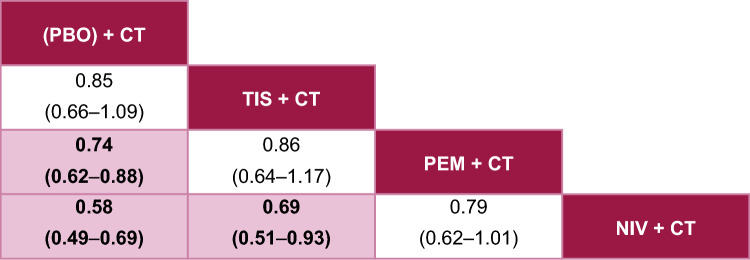
Fixed-effects League Table for Grade ≥ 3 TRAEs. Overall population; reported as OR [95% CrI], OR < 1 implies that column is better than row. Pink squares are statistically significant. The treatment with the most favorable estimate is positioned at the top left corner; the second, third, and fourth most favorable treatments are shown in descending order to the lower right. *CrI* credible interval, *CT* chemotherapy, *NIV* nivolumab, *OR* odds ratio, *PEM* pembrolizumab, *PBO* placebo, *TIS* tislelizumab, *TRAE* treatment-related adverse event

Tislelizumab + CT was associated with similar odds of grade ≥ 3 TRAEs compared with pembrolizumab + CT, with no statistically significant differences observed (OR 0.86, 95% CrI 0.64–1.17) (Fig. 7). Of note, a significantly lower odds of grade ≥ 3 TRAEs was observed for tislelizumab + CT compared with nivolumab + CT (OR 0.69, 95% CrI 0.51–0.93). Meanwhile, no statistically significant differences were observed between pembrolizumab + CT and nivolumab + CT. Further, both nivolumab + CT and pembrolizumab + CT were associated with significantly increased toxicity compared with CT alone. In contrast, tislelizumab + CT was not associated with significantly higher odds of grade ≥ 3 TRAEs compared with the CT control. Among the ICI + CT regimens, tislelizumab + CT had the highest SUCRA score of 64% (P-best 11%) (see Online Resource 3).

## Discussion

In this study, an SLR and NMA were performed to evaluate the comparative efficacy and safety of tislelizumab + CT relative to nivolumab + CT and pembrolizumab + CT in patients with unresectable, locally advanced, or metastatic GC/GEJC. The results demonstrated that tislelizumab + CT had comparable efficacy across OS, PFS, and ORR outcomes to nivolumab + CT and pembrolizumab + CT. Further, tislelizumab + CT was not associated with a statistically significant increase in grade ≥ 3 TRAEs compared with CT alone, and had lower odds of toxicity compared with nivolumab + CT, while no significant difference was observed between pembrolizumab + CT and nivolumab + CT. The differential safety profile of tislelizumab vs. nivolumab and pembrolizumab in this NMA may translate to a more favorable risk–benefit ratio for tislelizumab in GC/GEJC.

Comparative results for survival outcomes (OS and PFS) were largely consistent across subgroup analyses by PD-L1 status (i.e., patients with PD-L1 ≥ 1 [TAP score ≥ 1% or CPS ≥ 1] and ≥ 5 [TAP score ≥ 5% or CPS ≥ 5]). Likewise, no significant differences were observed between the PD-1 inhibitors with respect to ORR in subgroup analyses by PD-L1 status. To our knowledge, this is the first study to demonstrate similar comparative efficacy of tislelizumab to other PD-1 inhibitors at several clinically relevant PD-L1 expression cut-offs that correspond to its approved indications in 1L GC/GEJC in the EU (PD-L1 TAP score ≥ 5%) and the US (PD-L1 ≥ 1) [22, 23]. Although RATIONALE-305 used a different PD-L1 scoring method than comparator trials (TAP score instead of CPS), similar clinical benefits were demonstrated irrespective of scoring method used and a post hoc analysis of RATIONALE-305 data showed high concordance between CPS and TAP score, with an interclass correlation coefficient (ICC) of 0.81 (95% CrI 0.79–0.83) [25]. In this NMA, the results of a sensitivity analysis comparing OS in the PD-L1 ≥ 5 subgroup using CPS data from RATIONALE-305 instead of TAP scores further support the equivalence of these scores. Taken together, the data indicate that these two scoring methods are probably interchangeable with respect to their clinical utility. Further, the US label for tislelizumab does not explicitly specify the scoring method, noting that exploratory analyses of the OS benefit of tislelizumab observed in RATIONALE-305 was shown to be consistent across the two scoring methods [22].

The comparative OS results in subgroups by geographic region (Asia or ROW) and by primary tumor location (i.e., stomach or GEJ) were consistent with the base case, demonstrating that the three anti-PD-1 regimens were similarly efficacious in patients with GC/GEJC irrespective of these factors. This is an important observation given the geographic differences in GC/GEJC epidemiology and presentation [1, 45]. The results of the OS subgroup analysis in patients receiving capecitabine and oxaliplatin as the CT backbone were also consistent with the base case, demonstrating that tislelizumab performed similarly to both nivolumab and pembrolizumab in this patient subgroup. These findings suggest that there may be flexibility in choosing the CT backbone based on individual patient factors and institutional or regional preferences. Moreover, these findings validate the assumption of equivalent efficacy of the CT backbone made in this NMA and further strengthen the conclusions about the efficacy of tislelizumab + CT in the 1L treatment of HER2-negative GC/GEJC.

Although tislelizumab was engineered to minimize Fcγ receptor (FcγR) binding to reduce antibody-dependent cellular phagocytosis of PD-1-expressing T cells [46, 47], this theoretical efficacy advantage over nivolumab and pembrolizumab was not apparent in this NMA. Several reasons may explain this, including survival endpoints not being sensitive enough to detect subtle mechanistic advantages (particularly when compared indirectly), other immunosuppressive factors (e.g., regulatory T cells and myeloid-derived suppressor cells) dampening antitumor immunity, making the FcγR modification less impactful [48, 49], and the CT backbone masking subtle differences in immune modulation by altering immune responses [50].

A notable finding in this NMA is that the odds of grade ≥ 3 TRAEs were lower with tislelizumab + CT compared with nivolumab + CT. As comparative safety results may be influenced by differences in treatment duration and length of follow-up, it is noteworthy that the median treatment durations were similar across the included trials (~ 6 months), as were the durations of the protocol-specified safety assessments (during treatment and for ~ 1 month after treatment discontinuation) [24, 38–41]. Further, given that all trials used platinum-based CT doublet, it is unlikely that cross-trial differences in CT backbone (and the proportion of patients receiving each regimen) have impacted the results of these safety analyses. In support of this, the overall rates of grade ≥ 3 TRAEs were similar in the CT control arms across the included RCTs, suggesting that CT backbones were similarly tolerable [38–41, 44]. Specifically, in RATIONALE-305, where > 90% of patients received capecitabine plus oxaliplatin and a minority were treated with 5-fluouracil plus cisplatin, the incidence of grade ≥ 3 TRAEs in the CT control arm was 50% [24]. In the nivolumab trials, CheckMate 649 (where nearly half of patients received capecitabine plus oxaliplatin and the rest 5-fluorouracil plus oxaliplatin) and ATTRACTION-4 (where 36% of patients were treated with capecitabine plus oxaliplatin and the rest with tegafur-gimeracil-oteracil potassium plus oxaliplatin), the rates of grade ≥ 3 TRAEs were 44% and 49%, respectively [39, 41, 51]. This indicates that the lower odds of grade ≥ 3 TRAEs observed with tislelizumab + CT compared with nivolumab + CT in this NMA are unlikely to be due to a more tolerable CT backbone. Further, the reported rates of specific grade ≥ 3 TRAEs commonly associated with CT (e.g., nausea, diarrhea, and neutropenia) were similarly low across these trials [39, 41, 51]. The rates of grade ≥ 3 immune-mediated TRAEs were low and not consistently reported, precluding additional analyses.

Two other NMAs have evaluated tislelizumab + CT along with other immunotherapy regimens as 1L treatments of GC/GEJC, with the results being generally aligned with the present analysis [32, 33]. The NMA by Zhang et al. used the same pivotal trials for the comparators of interest, including the final analysis results of RATIONALE-305, and similarly reported no significant differences in OS or PFS between tislelizumab, nivolumab, and pembrolizumab + CT regimens [32, 33]. Reported HRs and CrIs were closely aligned with those of the present analysis: pembrolizumab + CT versus tislelizumab + CT (OS: HR 0.97; 95% CrI 0.82–1.16; PFS: HR 1.03; 95% CrI 0.85–1.24); nivolumab + CT versus tislelizumab + CT (OS: 1.02; 95% CrI 0.87–1.21; PFS: HR 1.01; 95% CrI 0.85–1.21). Although Zhang and colleagues also performed subgroup analyses by PD-L1 status, these analyses did not include tislelizumab and therefore cannot be compared with the results of the present study for these patient subgroups. Of interest, Zhang and colleagues found that nivolumab + CT was the only immunotherapy regimen associated with a significantly increased likelihood of grade ≥ 3 AEs compared with CT alone. Further, although non-significant, they also reported numerically increased odds of grade ≥ 3 AEs with nivolumab + CT compared with tislelizumab + CT, which agrees with the higher odds of grade ≥ 3 TRAEs observed for nivolumab + CT in the present study. As mentioned above, the present NMA is the first to demonstrate similar comparative efficacy of tislelizumab to other PD-1 inhibitors across key PD-L1 expression subgroups.

The present study has several strengths. First, analyses were performed according to best practice for conducting and reporting NMAs as described by NICE to ensure transparency and reproducibility [30]. The NMAs were also informed by a recent, comprehensive SLR in adherence to best practices provided by PRISMA guidance. Further, all trials included in the SLR underwent a rigorous feasibility assessment to highlight any sources of inter-trial heterogeneity to ensure the validity of results. Key clinical subpopulations were also identified with clinical expert opinion for analysis to reflect the diversity of indications and reimbursement criteria for comparator treatments. Of note, the current study adds to the growing evidence base supporting the use of tislelizumab + CT in the 1L treatment of GC/GEJC.

Despite these strengths, there are several potential limitations. First, network structures were sparse and some connections were informed by a single trial, which may bias treatment effect estimates and weaken the analysis. Second, the limited number of trials informing each comparison prevented the use of meta regression to adjust for potential sources of inter-trial heterogeneity. Further, indirect treatment comparisons like NMAs rely on the assumption that the included trials are sufficiently similar such that the effect estimates are not biased by underlying differences in patient populations. In this analysis, minimal between-trial heterogeneity was observed, and potential differences were explored using subgroup analyses where possible. As such, population-adjusted analyses were not conducted. It should be acknowledged, however, that CheckMate 649 enrolled adults with unresectable advanced or metastatic GC/GEJC or esophageal adenocarcinoma, the latter of which were not excluded from base case NMA analyses as they represented only a minority of enrolled patients (14%) and had comparable OS outcomes to patients with GC/GEJC. Additionally, safety subgroup analyses were precluded by limited reporting of safety outcomes for patient subgroups in comparator trials. Consequently, the interpretation and generalizability of the safety results is limited in the absence of subgroup analyses of grade ≥ 3 TRAEs. Lastly, caution may be warranted when interpreting the results of the safety analyses due to potential differences in safety reporting and in definitions of TRAEs.

In summary, the NMA results showed that tislelizumab + CT was comparable across efficacy outcomes to nivolumab + CT and pembrolizumab + CT, with significantly more favorable safety compared with nivolumab + CT and a similar safety profile to pembrolizumab + CT. Results of subgroup analyses of efficacy outcomes were also largely consistent with the base case analysis. Overall, based on results from the present analysis, tislelizumab + CT represents an effective 1L treatment option for patients with unresectable or metastatic HER2-negative GC/GEJC.

## Supplementary Information

Below is the link to the electronic supplementary material.

Supplementary file1 (DOCX 174 KB)

Supplementary file2 (DOCX 169 KB)

Supplementary file3 (DOCX 111 KB)

Supplementary file4 (DOCX 325 KB)

## Data Availability

BeOne Medicines (formerly known as BeiGene) voluntarily shares anonymous data on completed studies responsibly and provides qualified scientific and medical researchers access to anonymous data and supporting clinical trial documentation for clinical trials in dossiers for medicines and indications after submission and approval in the United States, China, and Europe. Clinical trials supporting subsequent local approvals, new indications, or combination products are eligible for sharing once corresponding regulatory approvals are achieved. BeOne Medicines shares data only when permitted by applicable data privacy and security laws and regulations. In addition, data can only be shared when it is feasible to do so without compromising the privacy of study participants. Qualified researchers may submit data requests/research proposals for BeOne Medicines review and consideration through BeOne Medicines’ Clinical Trial Webpage at https://www.beigene.com/our-science-and-medicines/our-clinical-trials/.
