# Supplementary material for: Comparative efficacy and safety of tislelizumab and other programmed cell death protein 1 inhibitors in first-line treatment of advanced gastroesophageal cancers: a systematic review and network meta-analysis
Source: Gastric Cancer. 2025 Oct 4;28(6):1021–32. doi: 10.1007/s10120-025-01660-4 (PMC12630173; doi:10.1007/s10120-025-01660-4)
Supplement: Supplementary file 1 — Supplementary file1 (DOCX 174 KB) [file 10120_2025_1660_MOESM1_ESM.docx]

# Supplementary material

Online Resource #1 for Comparative Efficacy and Safety of Tislelizumab and other Programmed Cell Death Protein 1 Inhibitors in First-line Treatment of Advanced Gastroesophageal Cancers: A Systematic Review and Network Meta-Analysis

Jaffer A. Ajani^1^ • Maria Alsina ^2^ • Markus Moehler^3^ • Keun-Wook Lee^4^ • Wenxi Tang^5^ • Jason Steenkamp^6^ • Emily Prentiss^6^ • Kaijun Wang^5^ • Becky Hooper^6^ • Lin Zhan^5^

^1^ Department of Gastrointestinal Medical Oncology, Division of Cancer Medicine, The University of Texas MD Anderson Cancer Center, Houston, TX, USA

^2^ Medical Oncology Department, Unidad de Oncología Médica Traslacional, Hospital Universitario de Navarra, Navarrabiomed – IdiSNA, Pamplona, Spain

^3^ Gastrointestinal Oncology, Johannes Gutenberg-University Clinic, Mainz, Germany

^4^ Department of Internal Medicine, Seoul National University College of Medicine, Seoul National University Bundang Hospital, Seongnam, Republic of Korea

^5^BeOne Medicines, Ltd. San Carlos, CA, USA

^6^Value & Evidence Services, EVERSANA, Burlington, ON, Canada

*** Correspondence:**Jaffer A. Ajani
[jajani@mdanderson.org](mailto:jajani@mdanderson.org)

713-792-2828

## Online Resource 1 – Systematic Literature Review

# Search Strategy

**Date of the search:** 16 Feb 2024

**Database searched:**

- Ovid MEDLINE(R)
- Ovid MEDLINE Epub Ahead of Print, In-Process & Other Non-Indexed Citations and Daily
- Ovid Embase
- Ovid EBM Reviews - Cochrane Central Register of Controlled Trials

• Ovid EBM Ovid EBM Reviews - Cochrane Database of Systematic Reviews

**Limits:**

RCTs Only - balanced RCT Filter version for Ovid MEDLINE only

Humans only

Adults - 18 years and older

Last 2 yrs of abstracts retained in Embase & CENTRAL

Trial protocols & Opinion publications – removed

No date limit

**Filters:**

**[Randomized Studies]**

(both MEDLINE and Embase filters have Phase 2-3 - additional terms to supplement RCTs filter) from:

Higgins JPT, Thomas J, Chandler J, Cumpston M, Li T, Page MJ, Welch VA (editors). *Cochrane Handbook for Systematic Reviews of Interventions* version 6.2 (updated February 2021). Cochrane, 2021. Available from [www.training.cochrane.org/handbook](http://www.training.cochrane.org/handbook).

**MEDLINE RCT Filter – Cochrane Handbook, 2019,** Box 3.d Cochrane Highly Sensitive Search Strategy for identifying randomized trials in MEDLINE: sensitivity- and precision-maximizing version (2008 revision); Ovid format <https://training.cochrane.org/handbook/current/chapter-04-technical-supplement-searching-and-selecting-studies#section-3-6-1>

**Embase RCT sensitive Filter – Cochrane Handbook, 2019, Box 3.e** Cochrane Highly Sensitive Search Strategy for identifying controlled trials in Embase: (2018 revision) <https://training.cochrane.org/handbook/current/chapter-04-technical-supplement-searching-and-selecting-studies#section-3-6-2>

**MULTIFILE SEARCH**

Database(s): **EBM Reviews - Cochrane Central Register of Controlled Trials**January 2024**, EBM Reviews - Cochrane Database of Systematic Reviews**2005 to February 14, 2024**, Embase**1974 to 2024 February 14**, Ovid MEDLINE(R) and Epub Ahead of Print, In-Process, In-Data-Review & Other Non-Indexed Citations and Daily**1946 to February 14, 2024

Search Strategy:

| **#** | **Searches** | **Results** |
| --- | --- | --- |
| 1 | Stomach Neoplasms/ or Gastroesophageal Junction Adenocarcinoma/ [Search design error: no MeSH for "Gastroesophageal Junction Adenocarcinoma"] | 131891 |
| 2 | (adenocarcinoma-GEJ or "esophagogastric adenocarcinoma" or "esophagogastric junction adenocarcinoma").af. | 1228 |
| 3 | (adenocarcinoma-GEJ or "esophagogastric adenocarcinoma" or "esophagogastric junction adenocarcinoma").ti,ab. | 1012 |
| 4 | ((gastric or stomach or gej) and (cancer* or carcinoma* or adenocarcinoma* or tumor* or tumour* or neoplasm* or malignan*)).af. | 506246 |
| 5 | or/1-4 | 506597 |
| 6 | (advanc* or malignan* or metastat* or unopera* or (non adj5 resect*) or nonresect* or unresect* or inopera* or (late adj2 stage) or stage iii or stage 3 or stage-3 or stage-iii or stage iv or stage 4 or stage-4 or (stage adj3 (iii or (adenocarcinoma-GEJ or "esophagogastric adenocarcinoma" or "esophagogastric junction adenocarcinoma") or c or iv or ((gastric or stomach or gej) and (cancer* or carcinoma* or adenocarcinoma* or tumor* or tumour* or neoplasm* or malignan*))))).af. | 6642925 |
| 7 | 5 and 6 | 187828 |
| 8 | limit 7 to english language [Limit not valid in CDSR; records were retained] | 163106 |
| 9 | 8 use medp,prem [Ovid MEDLINE® Segments: Epub Ahead of Print & In-Process & In-Data-Review Citations] | 558 |
| 10 | exp stomach cancer/ or exp stomach carcinoma/ or (stomach cancer or stomach carcinoma).af. | 281248 |
| 11 | Gastroesophageal Junction Adenocarcinoma/ or (adenocarcinoma-GEJ or "esophagogastric adenocarcinoma" or "esophagogastric junction adenocarcinoma").af. [Search design error: no MeSH for "Gastroesophageal Junction Adenocarcinoma"] | 1611 |
| 12 | ((exp neoplasm/ or exp adenocarcinoma/) and (gastric or stomach or gej or gastro-esophag* or gastro-oesophag* or gastro*esophageal junction).af.) or ((gastric or stomach or gej or gastro-esophag* or gastro-oesophag* or gastro*esophageal junction) and (cancer* or carcinoma* or adenocarcinoma* or tumor* or tumour* or neoplasm* or malignan*)).af. | 527011 |
| 13 | or/10-12 | 527616 |
| 14 | (advanc* or malignan* or metastat* or unopera* or (non adj5 resect*) or nonresect* or unresect* or inopera* or (late adj2 stage) or stage iii or stage 3 or stage-3 or stage-iii or stage iv or stage 4 or stage-4 or (stage adj3 (iii or (adenocarcinoma-GEJ or "esophagogastric adenocarcinoma" or "esophagogastric junction adenocarcinoma") or c or iv or ((gastric or stomach or gej) and (cancer* or carcinoma* or adenocarcinoma* or tumor* or tumour* or neoplasm* or malignan*))))).af. | 6642925 |
| 15 | 13 and 14 | 190338 |
| 16 | nivolumab/ or (nivolumab or bms 936558 or bms936558 or cmab 819 or cmab819 or mdx 1106 or mdx1106 or ono 4538 or ono4538 or opdivo).af. | 55452 |
| 17 | fluorouracil/ or (fluorouracil or 5-fluorouracil or 5 fluorouracil or 5fu or 5-fu or "5-fu lederle" or "5 fu lederle" or "5-fu medac" or "5 fu medac" or "5-hu hexal" or "5 hu hexal" or adrucil or carac or efudix or efudex or fluoroplex or flurodex or fluorouracile or dakota or fluracedyl or haemato-fu or "haemato fu" or neofluor or onkofluor or ribofluor).af. | 314899 |
| 18 | capecitabine/ or (capecitabine or xeloda or 154361-50-9).af. | 55820 |
| 19 | oxaliplatin/ or (oxaliplatin or oxaliplatine or eloxatine or eloxatin).af. | 79550 |
| 20 | cisplatin/ or (cisplatin or "mpi 5010" or mpi5010 or neoplatin or niyaplat or "nk 801" or nk801 or noveldexis or "nsc 119875" or platamine or "platamine rtu" or platiblastin or platidiam or platimine or platinex or platinil or platinol or "platinol aq" or "platinol-aq" or platinoxan or platiran or platistil or platistin or platosin or "pronto platamine" or randa or romcis or sicatem or "spi 077" or "spi 77" or spi077 or spi77 or tecnoplatin or "tr 170" or tr170).af. | 345803 |
| 21 | docetaxel/ or (docetaxel or taxotere or docefrez or "rp 56976" or "rp-56976" or 114977-28-5).af. | 107027 |
| 22 | tegafur/ or (tegafur or utefos or futraful or sunfural or uftoral or florafur or fluorofur or ftorafur).af. | 23748 |
| 23 | exp folinic acid/ or ("folinic acid" or "citrovorum factor" or wellcovorin or leucovorin or leukovorum or leucovorin).af. | 66157 |
| 24 | exp paclitaxel/ or (paclitaxel or abraxane or paxene or nsc-125973 or nsc125973 or anzatax or onxol or praxel or taxol or 33069-62-4).af. | 207025 |
| 25 | zolbetuximab/ or (zolbetuximab or claudiximab or imab362 or "imab 362").af. | 319 |
| 26 | bemarituzumab/ or (bemarituzumab or fpa144).af. | 117 |
| 27 | exp cetuximab/ or (cetuximab or erbitux or "imc c225" or imc-c225).af. | 47650 |
| 28 | (onartuzumab or oa-5d5 or metmab).af. | 797 |
| 29 | (rilotumumab or "amg 102").af. | 724 |
| 30 | pembrolizumab/ or (pembrolizumab or keytruda or lambrolizumab or "mk 3475" or mk3475 or "sch 900475" or sch900475).af. | 54626 |
| 31 | andecaliximab/ or (andecaliximab or gs-5745).af. | 186 |
| 32 | camrelizumab/ or (camrelizumab or carrelizumab or shr-1210 or "shr 1210" or shr1210 or hr-301210).af. | 3433 |
| 33 | (apatinib or rivoceranib or yn968d1 or "yn 968d1").af. | 5009 |
| 34 | avelumab/ or (avelumab or msb-0010682 or msb0010682 or bavencio or msb0010718c or msb-0010718c).af. | 8403 |
| 35 | pazopanib/ or ramucirumab/ or sintilimab/ or epirubicin/ or regorafenib/ or (pazopanib or gw786034 or ramucirumab or sintilimab or epirubicin or regorafenib).af. | 76875 |
| 36 | tislelizumab/ or (tislelizumab or bgb-a317).af. | 2416 |
| 37 | toripalimab/ or (toripalimab or js001).af. | 2166 |
| 38 | or/16-37 | 968351 |
| 39 | exp clinical trial/ or randomization/de or controlled study/de or comparative study/de or single blind procedure/de or double blind procedure/de or crossover procedure/de or placebo/de or prospective study/de or ("clinical trial" or "clinical trials" or "controlled clinical trial" or "controlled clinical trials" or "randomised controlled trial" or "randomised controlled trials" or "randomisation" or "randomization" or rct or "random allocation" or "randomly allocated" or "allocated randomly" or placebo* or (allocated adj2 random) or (random* adj1 assign*) or random* or ((single or double or triple or treble) adj1 (blind* or mask*))).ti,ab,kw,kf. | 7342682 |
| 40 | 15 and 38 and 39 | 14723 |
| 41 | (books or editorial or letter or note or "review" or short survey).pt. or (letter.pt. not randomized controlled trial/) or animal/ or case report/ or exp practice guideline/ or exp book/ [OPINION PIECES REMOVE - Embase] | 22779338 |
| 42 | 40 not 41 | 11771 |
| 43 | (conference abstract or conference paper or conference review).pt. | 5831545 |
| 44 | 42 and 43 | 2587 |
| 45 | limit 44 to yr="2019 -2022" | 777 |
| 46 | 42 not 43 | 9184 |
| 47 | 45 or 46 | 9961 |
| 48 | limit 47 to english language [Limit not valid in CDSR; records were retained] | 9034 |
| 49 | (systematic and review*).ti. | 553839 |
| 50 | (meta and analys*).ti. | 439028 |
| 51 | metaanalys*.ti. | 4347 |
| 52 | exp meta analysis/ | 502590 |
| 53 | exp systematic review/ | 704804 |
| 54 | "systematic review (topic)"/ | 34000 |
| 55 | or/49-54 | 1104452 |
| 56 | 40 and 55 | 762 |
| 57 | 47 or 56 | 10384 |
| 58 | limit 57 to english language [Limit not valid in CDSR; records were retained] | 9434 |
| 59 | 58 use oemezd [Embase results] | 4815 |
| 60 | Stomach Neoplasms/ [Search design error: this MeSH does not have subtrees so no need to explode] | 131489 |
| 61 | ((gastric or stomach or gej or gastro-esophag* or gastro-oesophag* or gastro*esophageal junction) and (cancer* or carcinoma* or adenocarcinoma* or tumor* or tumour* or neoplasm* or malignan*)).ti,ab,kw. | 389320 |
| 62 | (adenocarcinoma-GEJ or "esophagogastric adenocarcinoma" or "esophagogastric junction adenocarcinoma").ti,ab,kw. | 1063 |
| 63 | or/60-62 | 417457 |
| 64 | (advanc* or malignan* or metastat* or unopera* or (non adj5 resect*) or nonresect* or unresect* or inopera* or (late adj2 stage) or stage iii or stage 3 or stage-3 or stage-iii or stage iv or stage 4 or stage-4 or (stage adj3 (iii or (adenocarcinoma-GEJ or "esophagogastric adenocarcinoma" or "esophagogastric junction adenocarcinoma") or c or iv or ((gastric or stomach or gej) and (cancer* or carcinoma* or adenocarcinoma* or tumor* or tumour* or neoplasm* or malignan*))))).ti,ab,kw. | 5152802 |
| 65 | 63 and 64 | 156618 |
| 66 | Nivolumab/ [Search design error: this MeSH does not have subtrees so no need to explode] | 46817 |
| 67 | (nivolumab or bms 936558 or bms936558 or cmab 819 or cmab819 or mdx 1106 or mdx1106 or ono 4538 or ono4538 or opdivo).ti,ab,kw. | 33905 |
| 68 | exp Fluorouracil/ [Search design error: this MeSH does not have subtrees so no need to explode] | 221559 |
| 69 | (fluorouracil or 5-fluorouracil or 5 fluorouracil or 5fu or 5-fu or "5-fu lederle" or "5 fu lederle" or "5-fu medac" or "5 fu medac" or "5-hu hexal" or "5 hu hexal" or adrucil or carac or efudix or efudex or fluoroplex or flurodex or fluorouracile or dakota or fluracedyl or haemato-fu or "haemato fu" or neofluor or onkofluor or ribofluor).ti,ab,kw. | 138362 |
| 70 | Capecitabine/ | 46180 |
| 71 | (capecitabine or xeloda).ti,ab,kw. | 28803 |
| 72 | Oxaliplatin/ | 64907 |
| 73 | (oxaliplatin or oxaliplatine or eloxatine or eloxatin).ti,ab,kw. | 44400 |
| 74 | Cisplatin/ | 290095 |
| 75 | (cisplatin or "mpi 5010" or mpi5010 or neoplatin or niyaplat or "nk 801" or nk801 or noveldexis or "nsc 119875" or platamine or "platamine rtu" or platiblastin or platidiam or platimine or platinex or platinil or platinol or "platinol aq" or "platinol-aq" or platinoxan or platiran or platistil or platistin or platosin or "pronto platamine" or randa or romcis or sicatem or "spi 077" or "spi 77" or spi077 or spi77 or tecnoplatin or "tr 170" or tr170).ti,ab,kw. | 197179 |
| 76 | Docetaxel/ | 90732 |
| 77 | (docetaxel or taxotere or docefrez or "rp 56976" or "rp-56976" or 114977-28-5).ti,ab,kw. | 61498 |
| 78 | Tegafur/ | 13972 |
| 79 | (tegafur or utefos or futraful or sunfural or uftoral or florafur or fluorofur or ftorafur).ti,ab,kw. | 6576 |
| 80 | exp Leucovorin/ | 57772 |
| 81 | (leucovorin or leukovorum or leucovorin or "folinic acid" or "citrovorum factor" or wellcovorin).ti,ab,kw. | 27979 |
| 82 | exp Paclitaxel/ | 175262 |
| 83 | (paclitaxel or abraxane or paxene or nsc-125973 or nsc125973 or anzatax or onxol or praxel or taxol).ti,ab,kw. | 123080 |
| 84 | (zolbetuximab or claudiximab or imab362 or "imab 362").ti,ab,kw. | 254 |
| 85 | (bemarituzumab or fpa144).ti,ab,kw. | 82 |
| 86 | Cetuximab/ | 41047 |
| 87 | (cetuximab or erbitux or "imc c225" or imc-c225).ti,ab,kw. | 26677 |
| 88 | (onartuzumab or oa-5d5 or metmab).ti,ab,kw. | 349 |
| 89 | (rilotumumab or "amg 102").ti,ab,kw. | 238 |
| 90 | (pembrolizumab or keytruda or lambrolizumab or "mk 3475" or mk3475 or "sch 900475" or sch900475).ti,ab,kw. | 32722 |
| 91 | (andecaliximab or gs-5745).ti,ab,kw. | 132 |
| 92 | (camrelizumab or carrelizumab or shr-1210 or "shr 1210" or shr1210 or hr-301210).ti,ab,kw. | 1910 |
| 93 | (apatinib or rivoceranib or yn968d1 or "yn 968d1").ti,ab,kw. | 3781 |
| 94 | (avelumab or msb-0010682 or msb0010682 or bavencio or msb0010718c or msb-0010718c).ti,ab,kw. [Search design error: missing MeSH] | 3518 |
| 95 | (pazopanib or gw786034).ti,ab,kw. | 7422 |
| 96 | (tislelizumab or bgb-a317).ti,ab,kw. | 1283 |
| 97 | (toripalimab or js001).ti,ab,kw. | 1061 |
| 98 | Epirubicin/ | 40489 |
| 99 | (epirubicin or imi-28 or "imi 28" or epilem or epi-cell or farmorubicin).ti,ab,kw. | 18613 |
| 100 | (ramucirumab or ly3009806 or cyramza or "imc 1121b" or imc1121b or imc-1121b or 1121b).ti,ab,kw. [Search design error: missing MeSH] | 4342 |
| 101 | (sintilimab or ibi308 or ibi-308 or "ibi 308" or tyvyt).ti,ab,kw. | 1605 |
| 102 | (regorafenib or stivarga or msb-0010718c or resihance).ti,ab,kw. | 6459 |
| 103 | or/66-102 | 889648 |
| 104 | 65 and 103 | 29675 |
| 105 | 104 use cctr,coch | 3102 |
| 106 | 65 and 98 [Check for typo in the original Novartis Cochrane Reviews, Trials search (Line #40: #6 AND #39 which should be #6 AND #44)] | 2215 |
| 107 | 106 use cctr,coch [Proves that the Line #40: #6 AND #39 was a typo since the recall in the original Novaris search was 2903 hits] | 97 |
| 108 | 9 or 59 or 107 | 5470 |
| 109 | remove duplicates from 108 | 5301 |
| 110 | limit 9 to dt="20220701-20241231" [Limit not valid in CCTR,CDSR,Embase; records were retained] | 402 |
| 111 | limit 59 to dc="20220701-20241231" [Limit not valid in CCTR,CDSR; records were retained] | 681 |
| 112 | 107 and (202207$ or 2202208$ or 202209$ or 202210$ or 202211$ or 202212$ or 2023$ or 2024$).up. | 29 |
| 113 | 110 or 111 or 112 | 1112 |
| 114 | limit 113 to yr="2022 -Current" [Isolate results set published within the date span Jul 2022 - Current - since the date Novartis search was run] | 1063 |
| **115** | **108 not 114** | **4407** |
| 116 | Stomach Neoplasms/ or (Esophageal Neoplasms/ and exp Esophagogastric Junction/) | 132993 |
| 117 | Neoplasm Metastasis/ or Neoplasm Recurrence, Local/ | 503214 |
| 118 | ((((stomach? or gastric$ or cardia or cardiac or antrum? or antral$ or fundus$ or pyloric$ or pylorus$ or ventricul$ or linitis plastica or leather-bottle or ((stomach? or gastric$) and (GC or GEJ))) adj3 (neoplas$ or cancer$ or tumo?r$ or carcinoma$ or malignan$ or oncolog$ or adenocancer$ or adeno-cancer$ or adenoma$ or adenocarcinoma$ or adeno-carcinoma$ or blastoma$ or carcinosarcoma$ or carcino-sarcoma$ or adenoacanthoma$ or adeno-acanthoma$ or epithelioma$ or melanoma$ or mesenchymoma$ or sarcoma$ or thymoma$ or granuloma$ or choriocarcinoma$ or cancerogenes?s or carcinoid$)) or ((stomach? or gastric$) adj3 SCC) or ((esophag$ or oesophag$ or esophagogastric$ or esophago-gastric$ or oesophagogastric$ or oesophago-gastric$ or gastroesophageal$ or gastro-esophageal$ or gastrooesophageal$ or gastro-oesophageal$ or cardio-esophageal$ or cardio-oesophageal$ or cardioesophageal$ or cardiooesophageal$ or EG or GE) adj3 (junction$ or sphincter$) adj3 (neoplas$ or cancer$ or tumo?r$ or carcinoma$ or malignan$ or oncolog$ or adenocancer$ or adeno-cancer$ or adenoma$ or adenocarcinoma$ or adeno-carcinoma$ or blastoma$ or carcinosarcoma$ or carcino-sarcoma$ or adenoacanthoma$ or adeno-acanthoma$ or epithelioma$ or melanoma$ or mesenchymoma$ or sarcoma$ or thymoma$ or granuloma$ or choriocarcinoma$ or cancerogenes?s or carcinoid$))) adj4 ((meta adj sta$) or metastas$ or metastatic$ or recur$ or secondar$ or relaps$ or advance$ or inoperab$ or disseminat$ or spread or migration or lethal$ or incurable or noncurable or non-curable or uncurable or progressive or terminal or invasive$ or aggressive$ or (late? adj2 stage$) or ((stage? or grade? or type?) adj2 (3a$ or 3b$ or 3c$ or III$ or 4a$ or 4b$ or IV$)) or "stage 3" or "stage 4" or met or mets or N1? or N2? or N3? or pN1? or pN2? or pN3?)).ti,ab,kw,kf. [Metastatic GC/GEJ TERMS] | 71738 |
| 119 | (116 and 117) or 118 [GC-GEJ TERMS] | 76938 |
| 120 | (tislelizumab$2 or tirelizumab$2 or bgb-a317 or bgba317 or bgn-1 or bgn1 or jhl-2108 or jhl2108 or vdt-482 or vdt482 or 1858168-59-8 or 0kvo411b3n).ti,ab,kw,kf,ot,hw,rn,nm. [TISLELIZUMAB TERMS] | 2437 |
| 121 | Immune Checkpoint Inhibitors/ or ((Programmed Cell Death 1 Receptor/ or Programmed Cell Death 1 Ligand 2 Protein/) and (inhibit$ or block?).ti,ab,kw,kf.) or ((immune$ adj3 checkpoint? adj3 (inhibit$ or block?)) or (((programmed adj3 death) or PD-1 or PD-1-PD-L1 or PDCD1) adj3 (ligand? or inhibit$ or block?)) or ((B7-H1 or B7H1 or "B7 homolog 1" or CD274 or CD273 or PDCD1LG1 or PDCD1LG2) adj3 (antigen? or protein?)) or ((Cytotoxic-T-Lymphocyte-Associated Protein-4 Inhibitor? or CTLA-4) adj3 (inhibit$ or block?)) or (ICI? and "Immun$ Checkpoint") or BMS-1 or EX-A947 or HY-19991 or J-690233 or MFCD28978741 or s7911 or D000082082 or SCHEMBL16555159 or ZINC230477930 or 1675201-83-8).ti,ab,kw,kf,ot,hw,rn,nm. [IMMUNE CHECKPOINT PROTEINS TERMS] | 174996 |
| 122 | Immunotherapy/ or Radioimmunotherapy/ or Antibodies, Monoclonal/ or (immunotherap$ or immuno-therap$ or (((biologic$ adj3 response? adj3 modifier?) or BRM or immunogenic$ or immunologic$ or immuno-genic$ or immuno-logic$ or radioimmunotherapy$ or radio-immunotherap$ or ((monoclonal$ or clonal$ or hybridoma$) adj2 antibod$)) adj3 (therap$ or intervention? or treat$))).ti,ab,kw,kf. [IMMUNOTHERAPY TERMS] | 898956 |
| 123 | Molecular Targeted Therapy/ or ((molecular$ or neoplas$ or cancer$ or tumo?r$ or carcinoma$) adj3 (target$ adj3 therap$)).ti,ab,kw,kf. [TARGETED THERAPY TERMS] | 189745 |
| 124 | (atezolizumab$2 or anti-PDL1 or MPDL-3280A or MPDL3280A or RG-7446 or RG7446 or ro-5541267 or ro5541267 or Tecentriq$2 or Tecntriq$2 or 1380723-44-3 or 0INE2SFD9E or 52CMI0WC3Y).ti,ab,kw,kf,ot,hw,rn,nm. [ATEZOLIZUMAB TERMS] | 23649 |
| 125 | (avelumab$2 or bavencio$2 or msb-0010682 or msb-0010718c or msb0010682 or msb0010718c or msb-10682 or msb-10718c or msb10682 or msb10718c or pf-06834635 or pf-6834635 or pf06834635 or pf6834635 or KXG2PJ551I or 1537032-82-8).ti,ab,kw,kf,ot,hw,rn,nm. [AVELUMAB TERMS] | 8386 |
| 126 | (camrelizumab$2 or "anti-pd-1 monoclonal antibody" or shr-1210 or shr1210 or carilizumab$2 or carrelizumab$2 or 73096E137E or 1798286-48-2).ti,ab,kw,kf,ot,hw,rn,nm. [CAMRELIZUMAB TERMS] | 4464 |
| 127 | (1428935-60-7 or 28x28x9okv or anti-b7h1-monoclonal-antibody or durvalumab$2 or durvalumabum$2 or imfinzi$2 or l01xc28 or medi4736 or medi-4736).ti,ab,kw,kf,ot,hw,rn,nm. [DURVALUMAB TERMS] | 14679 |
| 128 | Ipilimumab/ or (ipilimumab$2 or bms-734016 or bms734016 or cs-1002 or cs1002 or ibi-310 or ibi310 or mdx-ctla-4 or mdx-010 or mdx-101 or mdx010 or mdx101 or strentarga$2 or yervoy$2 or 6T8C155666 or 477202-00-9).ti,ab,kw,kf,ot,hw,rn,nm. [IPILIMUMAB TERMS] | 34734 |
| 129 | Nivolumab/ or (nivolumab$2 or bms-936558 or bms-986213 or bms-986298 or cmab819 or bms936558 or bms986213 or bms986298 or cmab-819 or mdx-1106 or mdx1106 or ono-4538 or ono4538 or opdivo$2 or opdualag$2 or 31YO63LBSN or 946414-94-4).ti,ab,kw,kf,ot,hw,rn,nm. [NIVOLUMAB TERMS] | 55423 |
| 130 | (pembrolizumab$2 or keytruda$2 or lambrolizumab$2 or mk3475 or mk-1308a or mk-3475 or mk7684a or sch-900475 or sch900475 or "keylynk-010 component" or DPT0O3T46P or 1422183-02-5 or 1374853-91-4).ti,ab,kw,kf,ot,hw,rn,nm. [PEMBROLIZUMAB TERMS] | 54555 |
| 131 | (2072873-06-2 or 8fu7fq8upk or ibi308 or ibi-308 or sintilimab$2 or tyvyt$2 or who-10801).ti,ab,kw,kf,ot,hw,rn,nm. [SINTILIMAB TERMS] | 3049 |
| 132 | (1924598-82-2 or 8jxn261vva or js001 or js-001 or tab001 or tab-001 or teripalimab$2 or toripalimab$2 or treipril$2 or treprizumab$2 or tripleitriumab$2 or triprizumab$2 or tuoyi$2 or who-10820).ti,ab,kw,kf,ot,hw,rn,nm. [TORIPALIMAB TERMS] | 2185 |
| 133 | (2079108-44-2 or 2226345-85-1 or 2y3t5if01z or aex1188 or aex-1188 or incmga00012 or incmga-00012 or incmga0012 or incmga-0012 or mga012 or mga-012 or retifanlimab$2 or zynyz$2).ti,ab,kw,kf,ot,hw,rn,nm. [RETIFANLIMAB TERMS] | 239 |
| 134 | (2102192-68-5 or anti-pd-l1-monoclonal-antibody-kn035 or asc22 or asc-22 or envafolimab$2 or es1m06m6qh or kn035 or kn-035).ti,ab,kw,kf,ot,hw,rn,nm. [ENVAFOLIMAB TERMS] | 199 |
| 135 | (2245725-04-4 or l62556gpxb or mgd013 or mgd-013 or tebotelimab$2).ti,ab,kw,kf,ot,hw,rn,nm. [TEBOTELIMAB TERMS] | 99 |
| 136 | (2394841-59-7 or 6fyg1ds4nw or ak104 or ak-104 or cadonilimab$2 or who-11581).ti,ab,kw,kf,ot,hw,rn,nm. [CADONILIMAB TERMS] | 155 |
| 137 | (2231029-82-4 or hlx10 or hlx-10 or s3gqz2k36v or serplulimab$2).ti,ab,kw,kf,ot,hw,rn,nm. [SERPLULIMAB TERMS] | 186 |
| 138 | (2256084-03-2 or 90iqr2i6tr or cs1001 or cs-1001 or sugemalimab$2 or wbp315 or wbp-315 or wbp3155 or wbp-3155).ti,ab,kw,kf,ot,hw,rn,nm. [SUGEMALIMAB TERMS] | 199 |
| 139 | (1496553-00-4 or claudiximab$2 or imab362 or imab-362 or tf5mpq8wgy or zolbetuximab$2).ti,ab,kw,kf,ot,hw,rn,nm. [ZOLBETUXIMAB TERMS] | 318 |
| 140 | (1952272-74-0 or bemarituzumab$2 or fpa144 or fpa-144 or rjw23bq0kw).ti,ab,kw,kf,ot,hw,rn,nm. [BEMARITUZUMAB TERMS] | 129 |
| 141 | Cetuximab/ or (205923-56-4 or abp494 or abp-494 or c225 or c-225 or c225-03 or c-22503 or c-225-03 or cdp1 or cdp-1 or cetuximab$2 or cetuximabum$2 or ch225 or ch-225 or chimeric-anti-egfr-monoclonal-antibody or cmab009 or cmab-009 or ctp15 or ct-p15 or dtxsid0040830 or erbitux$2 or hsdb-7454 or imc225 or imc-225 or imcc225 or imcc-225 or imc-c225 or kl140 or kl-140 or l01xc06 or ly2939777 or ly-2939777 or mab-c225 or moab-c225 or nsc714692 or pqx0d8j21j or sti001 or sti-001).ti,ab,kw,kf,ot,hw,rn,nm. [CETUXIMAB TERMS] | 48593 |
| 142 | (1133766-06-9 or metmab$2 or metma-b or ms1j9720wc or onartuzumab$2 or pro143966 or pro-143966 or pro-143996 or pro143996 or ro5490258 or ro-5490258).ti,ab,kw,kf,ot,hw,rn,nm. [ONARTUZUMAB TERMS] | 778 |
| 143 | (51wew898ij or 872514-65-3 or amg102 or amg-102 or rilotumumab$2).ti,ab,kw,kf,ot,hw,rn,nm. [RILOTUMUMAB TERMS] | 725 |
| 144 | (1518996-49-0 or 571045eim4 or andecaliximab$2 or gs5745 or gs-5745).ti,ab,kw,kf,ot,hw,rn,nm. [ANDECALIXIMAB TERMS] | 186 |
| 145 | (444731-52-6 or 635702-64-6 or 790713-33-6 or 7rn5dr86ck or a19406 or a839572 or ab01273967-01 or ab01273967-02 or ab01273967-05 or ab01273967-06 or ac-8522 or akos005145819 or am20090659 or ar-270-43507999 or armala$2 or as-11066 or bcp01839 or bcp9001053 or bcpp000129 or bd164238 or bdbm26474 or brd-k74514084-003-02-7 or ccg-265010 or chebi-71219 or chembl477772 or cid-10113978 or cs-0269 or db06589 or dtxcid1028659 or dtxsid8048733 or en300-57325 or ex-a1241 or ft-0659928 or ft-0684794 or gtpl5698 or gw780604 or gw-780604 or gw-78603 or gw786034 or gw7-86034 or gw-786034 or gw786034b or gw-786034b or gw786034x or gw-786034x or hms3244c21 or hms3244c22 or hms3244d21 or hms3656l14 or hms3745g05 or hsdb-8210 or hy-10208 or indazolylpyrimidine-13 or jmc514632-compound-13 or kinome-3790 or mfcd11616589 or ncgc00188865-01 or ncgc00188865-02 or ncgc00188865-03 or ncgc00188865-10 or nsc752782 or nsc-752782 or nsc800839 or nsc-800839 or p-6706 or pazopanib$2 or pazopanibum$2 or q-101400 or q7157043 or s3012 or sb17290 or sb710468 or sb-710468 or sb710468a or sb-710468a or schembl588608 or sw218082-3 or tox21-113174 or tox21-113174-1 or votrient$2 or z1541638525).ti,ab,kw,kf,ot,hw,rn,nm. [PAZOPANIB TERMS] | 14645 |
| 146 | (1218779-75-9 or 5s371k6132 or 811803-05-1 or ab01274807-01 or ab01274807-02 or ac-27461 or akos024464453 or amy21302 or apatinib$2 or ba175030 or bcp02840 or c76598 or ccg-268625 or chembl3186534 or cs-0003200 or d11288 or db14765 or ds-7455 or dtxsid601024366 or ex-a1794 or gtpl7648 or hy-13342a or mfcd21648511 or ncgc00249393-01 or ncgc00249393-08 or nsc772886 or nsc-772886 or nsc799333 or nsc-799333 or q27262801 or rivoceranib$2 or s5248 or sb16590 or schembl1814966 or yn968d1 or yn-968d1).ti,ab,kw,kf,ot,hw,rn,nm. [RIVOCERANIB/APATINIB TERMS] | 5000 |
| 147 | Induction Chemotherapy/ or Consolidation Chemotherapy/ or Maintenance Chemotherapy/ or Antineoplastic Combined Chemotherapy Protocols/ or exp Chemotherapy, Adjuvant/ or Chemoradiotherapy/ or (chemotherap$ or chemo-therap$ or carcinochemotherap$ or chemoradiotherap$ or chemoradiation? or radiochemotherap$ or carcino-chemotherap$ or chemo-radiotherap$ or chemo-radiation? or radio-chemotherap$).ti,ab,kw,kf. [CHEMOTHERAPY TERMS] | 1842747 |
| 148 | exp Leucovorin/ or (leucovorin$ or 6-s-leucovorin or 6s-leucovorin or acide folinique or akos015961207 or bdbm50039121 or bpbio1-000766 or bspbio-000696 or bspbio-002218 or brd-a75919782-238-01-8 or calcium folinate or chebi-15640 or chembl1679 or chembl69905 or citrovoeum-factor or citrovorum-factor or d93089 or divk1c-000222 or dtxsid0048216 or einecs-200-361-6 or en300-27068710 or folinate folinic-acid-sf or folinic acid or formyltetrahydrofolate or fusilev$2 or gtpl4816 or gtpl6690 or hsdb-6544 or hy-17556 or idi1-000222 or kbio1-000222 or kbio2-001339 or kbio2-003907 or kbio2-006475 or kbio3-001438 or kbiogr-000461 or kbioss-001339 or lencovorin$2 or leucal$2 or leukovorin$2 or leukovoran$2 or leucovorin$2 or levoleucovorin$2 or levo-leucovorin$2 or mfcd00867488 or ninds-000222 or nsc3590 or prestwick0-000738 or prestwick1-000738 or prestwick2-000738 or prestwick3-000738 or q45435667 or q573i9dvlp or s5790 or schembl10068238 or schembl8349 or sd-204098 or s-leucovorin$2 or sleucovorin$2 or spectrum2-000116 or spectrum3-000479 or spectrum4-000031 or spectrum5-000910 or spectrum-000859 or spbio-000132 or spbio-002635 or sbi-0051427-p003 or welcovorin$2 or "formyltetrahydropteroylglutamic acid" or Q573I9DVLP or 58-05-9).ti,ab,kw,kf,ot,hw,rn,nm. [LEUCOVORIN TERMS] | 68277 |
| 149 | Carboplatin/ or (Carboplatin$2 or blastocarb$2 or boplatex$2 or carboplat$ or carbosin$2 or carbotec$2 or carplan$2 or CBDCA or (platinum adj3 (cis or diamin? or cyclobutanedicarboxylat? or dicarboxylatediammine)) or Dicarboxylatoplatinum or diamminecyclobutanedicarboxylatoplatinum or cycloplatin$ or erbakar$2 or ercar$2 or ifacap$2 or jm8 or jm-8 or kemocarb$2 or nsc-241240 or nsc241240 or nsc-201345 or nsc201345 or oncocarbin$2 or paraplatin$ or Platinwas$2 or Ribocarbo$2 or Neocarbo$2 or Nealorin$2 or HSDB-6957 or BG3F62OND5 or 41575-94-4).ti,ab,kw,kf,ot,hw,rn,nm. [CARBOPLATIN TERMS] | 129374 |
| 150 | exp Paclitaxel/ or (paclitaxel$ or abraxane$2 or abraxus$2 or act02709 or act-02709 or acon1-002231 or anx-513 or anzatax$2 or apealea$2 or asotax$2 or bidd-pxr0046 or biotax$2 or bms-181339 or bms181339-01 or bms181339 or bms-181339-01 or bmy-45622 or bmy45622 or bspbio-000290 or capxol$2 or ccris-8143 or chembl428647 or chebi-45863 or coroflex-please$2 or coroxane$2 or cmap-000068 or cynviloq$2 or cypher-select$2 or dsstox-cid-3413 or dsstox-gsid-23413 or dsstox-rid-77016 or dhp107 or dhp-107 or dhp-208 or dhp208 or dts-301 or dts301 or ebetaxel$2 or empac$2 or endotag-1 or endotag1 or formoxol$2 or genaxol$2 or genetaxyl$2 or genexol$2 or gtpl2770 or hms2090d07 or hms2095o12 or hms2231a16 or hms3712o12 or hsdb-6839 or hunxol$2 or hy-b0015 or ifaxol$2 or ig-001 or ig001 or infinnium$2 or intaxel$2 or kbiogr-002509 or kbio2-002509 or kbio2-005077 or kbio2-007645 or kbio3-002987 or lep-etu$2 or lipopac$2 or liporaxel$2 or mbt-0206 or mbt0206 or medixel or mfcd00869953 or mitotax$2 or nanopac$2 or nanotax$2 or nanotaxel$2 or ncgc00164367-01 or nk-105 or nk105 or nsc-125973 or nsc-673089 or nsc125973 or nsc673089 or nscc-125973 or nova-12005 or oas-pac-100 or oaspac100 or oncogel$2 or onxal$2 or onxol$2 or p-ssmm-vip$2 or paclical$2 or pacitaxel$2 or paclical$2 or padexol$2 or pacligel$2 or paclitaxel$2 or pacliex$2 or paxceed$2 or paxene$2 or paxoral$2 or paxus$2 or pazenir$2 or plaxicel$2 or praxel$2 or qw-8184 or schembl3976 or sb-05 or sb05 or sdp-013 or sindaxel$2 or smr000857385 or sr-01000075350 or taycovit$2 or taxalbin$2 or taxane$ or taxocris$2 or taxol$2 or taxus$2 or tocosol$2 or xorane$2 or yewtaxan$2 or zinc96006020 or zisu$2 or P88XT4IS4D or 33069-62-4).ti,ab,kw,kf,ot,hw,rn,nm. [PACLITAXEL TERMS] | 238687 |
| 151 | Docetaxel/ or (114915-20-7 or 114977-28-5 or 15h5577cqd or 699121phca or ab01273941-01 or ab01273941-02 or ac-383 or akos015960718 or akos024457953 or amy4356 or anx-514 or axtere$2 or bd164373 or bdbm36351 or bind014 or bind-014 or brd-k30577245-001-04-3 or brd-k30577245-341-01-9 or bs102 or bs-102 or chebi-4672 or chembl92 or cid148124 or ckd-810 or crlx301 or crlx-301 or cs-1144 or d07866 or d4102 or daxotel$2 or db01248 or dexotel$2 or docecad$2 or docefrez$2 or docetaxel$2 or docetaxelum$2 or docetaxol$2 or docetaxolum$2 or dtxcid8020464 or dtxsid0040464 or emdoc$2 or en300-123047 or ex-a1206 or gtpl6809 or hms2089k08 or hsdb-6965 or hy-b0011 or ks-1452 or l01cd02 or lit976 or lit-976 or mfcd00871399 or ncgc00181306-01 or ncgc00181306-02 or ncgc00181306-04 or ncgc00242509-01 or nsc628503 or nsc-628503 or nsc-759850 or oncodocel$2 or q-100074 or q420436 or rp56976 or rp-56976 or schembl4419 or sdp-014 or sid-530 or sr-01000003023 or sr-01000003023-5 or syp-0704a or taxanit$2 or taxespira$2 or taxoel$2 or taxoltere-metro or taxotel$2 or taxoter$2 or taxotere$2 or texot$2 or tox21-112781 or tox21-113088 or txl$2 or w-60384 or xrp6976 or xrp-6976 or xrp-6976l or z1546621742).ti,ab,kw,kf,ot,hw,rn,nm. [DOCETAXEL TERMS] | 108006 |
| 152 | Cisplatin/ or (Cisplatin$ or platinum$ or Cismaplat$2 or (cis adj3 ($platinum or platinous)) or cis-platinum or cis-Platin or dichloroplatinum or diaminodichloroplatinum or diamminedichloroplatinum or dichlorodiammineplatinum or AI3-62048 or abiplatin or biocisplatinum or biocysplatinum or blastolem$2 or briplatin$2 or cddp-ti or cis-ddp or cisPt$ or CACP or CCRIS-221 or CDDP or DDPt or CP-Ethypharm or CPDC or CPDD or CPPD or (DDP and antitumor) or cisplatyl$2 or citoplatino$2 or cytoplatin$2 or cytosplat$2 or docistin$2 or elvecis$2 or kemoplat$2 or Fauldiscipla$2 or IA-call or LiPlaCis$2 or lederplatin$2 or lipoplatin$2 or "liposomal cisplatin" or mpi-5010 or mpi5010 or neoplatin$2 or niyaplat$2 or nk-801 or noveldexis$2 or nsc-119875 or nci-c55776 or platamine$2 or platamine-rtu or platiblastin$2 or platidiam$2 or platimine$2 or platinex$2 or platinil$2 or platino$2 or platinol$2 or platinolaq$2 or platinol-aq$2 or platinoxan$2 or platiran$2 or platistil$2 or platistin$2 or platosin$2 or "pronto platamine" or "Peyrone's chloride" or randa$2 or romcis$2 or sicatem$2 or spi-077 or tr-170 or tecnoplatin$2 or Q20Q21Q62J or 15663-27-1 or 26035-31-4 or 96081-74-2).ti,ab,kw,kf,ot,hw,rn,nm. [CISPLATIN TERMS] | 471194 |
| 153 | Oxaliplatin/ or (oxaliplatin$2 or (Oxalat$ adj3 platin$) or axiplatin$2 or bendaplatin$2 or crisapla$2 or croloxat$2 or dacotin$2 or dacplat$2 or ebeoxal$2 or elatofen$2 or eloxatin$ or elplat$2 or euroxaliplatin$2 or geneplatin$2 or gessedil$2 or heloxatin$2 or lipoxal$2 or mbp-426 or mbp426 or medoxa$2 or oksaliplatin$ or oplat$2 or oxalatoplatin$ or oxalatplatin$2 or oxali$2 or oxalip$2 or oxaliplan$2 or oxaliprol$2 or oxaliquid$2 or oxalisan$2 or oxalisin$2 or oxalizor$2 or oxaltic$2 or oxaltin$2 or oxamed$2 or oxaplamyl$2 or oxaviatin$2 or platox$2 or plaxitin$2 or rectoxal$2 or riboxatin$2 or rp-54780 or rp54780 or sinoxal$2 or sr-96669 or sr96669 or transplastin$2 or velminox$2 or xaliplat$2 or xoplan$2 or L-OHP-Cpd or 1-OHP or ACT-078 or ACT078 or CCRIS-9143 or NSC-266046 or 04ZR38536J or 61825-94-3).ti,ab,kw,kf,ot,hw,rn,nm. [OXALIPLATIN TERMS] | 104675 |
| 154 | Capecitabine/ or (capecitabin$ or apecitab$2 or atubri$2 or bc164277 or bcpp000300 or bxeliri$2 or bs-1000 or cacit$2 or capcel$2 or capebina$2 or capecite$2 or capegard$2 or capezam$2 or capicet$2 or capiri$2 or capiibine$2 or captabin$2 or capnat$2 or capoda$2 or capostat$2 or capsy$2 or capxcel$2 or caxeta$2 or ccg-264841 or ccx-340 or cpecitabine$2 or cs-0768 or d01223 or db01101 or dsstox-cid-26451 or dsstox-gsid-46451 or dsstox-rid-81625 or dtxsid3046451 or ecansya$2 or ex-a835 or gtpl6799 or hsdb-7656 or hy-b0016 or j-700154 or k007 or m0297 or mfcd00930626 or mls003915642 or mls004774137 or ncgc00164569-01 or ncgc00164569-02 or ncgc00164569-05 or nsc-759853 or paxon$2 or q-200788 or q420207 or r-340 or rg-340 or r340 or rg340 or ro-09-1978 or ro-091978 or ro-09-1978 or ro-09-1978-000 or ro091978 or s1156 or s-1156 or sr-01000931255 or tox21-112198 or x-abine$2 or x-tabin$2 or xabine$2 or xecap$2 or xeliri$2 or xelocel$2 or xeloda$2 or xelox$2 or z1501480421 or zinc3806413 or 6804dj8z9u or 154361-50-9 or 958887-39-3).ti,ab,kw,kf,ot,hw,rn,nm. [CAPECITABINE TERMS] | 57834 |
| 155 | (platinum adj1 (fluoropyrimidine or fluoro-pyrimidine) adj3 (doublet? or combin$ or chemotherap$ or chemo-therap$ or ((first or front) adj1 line?) or 1-LOT or 1L or therap$ or regimen? or expos$)).ti,ab,kw,kf,ot,hw,rn,nm. [PLATINUM-FLUOROPYRIMIDINE DOUBLET TERMS] | 254 |
| 156 | (5-fluoropyrimidin$ or 5-fluoro-pyrimidine or pyrimidine-5-fluoro or (fluorinated adj1 pyrimidine) or a9048 or ac-453 or akos006346044 or am86123 or "bb 0260992" or c4h3fn2 or db-007051 or dtxsid80217851 or en300-6966105 or f14737 or ft-0601423 or mfcd06658278 or q42859845 or w-203496 or zinc1845840 or 675f218 or L36X4TD47C or 675-21-8).ti,ab,kw,kf,ot,hw,rn,nm. [FLUOROPYRIMIDINE TERMS] | 5169 |
| 157 | Fluorouracil/ or (fluorouracil$ or fluroblastin$ or 1upf or 5-Faracil or 5-Fluoracil or 5-Fluoracyl or 5-fluoro-uracil or 5-fluoro-uracil or 5-Fluoroblastin or 5-fluorouacil or 5-Ftouracyl or 5-FU or 5FU or 5F-uracil or Adrucil$2 or AI3-25297 or Arumel$2 or BSPBio-002048 or Cancersil$2 or Carac$2 or Carzonal$2 or CHEBI-46345 or CHEMBL185 or Cinco-FU or CCRIS-2582 or DSSTox-CID-634 or DSSTox-GSID-20634 or DSSTox-RID-75705 or Efudex$2 or Efudix$2 or Efurix$2 or EINECS-200-085-6 or Effluderm$2 or Fluoroblastin$2 or Fluoro-Uracil$2 or Fluoro-uracile$2 or Fluoro-uracilo$2 or Fluoroplex$2 or Fluorouracile$2 or Fluorouracilo$2 or Fluorouracilum$2 or Fluorouracilum$2 or Fluracil$2 or Fluracilum$2 or Fluri$2 or Fluril$2 or Fluuro-Uracil$2 or Fluorouracilo$2 or Fluroblastin$2 or Fluro-Uracil$2 or Ftoruracil$2 or GTPL4789 or HSDB 3228 or IDI1-000054 or Kecimeton$2 or KBio1-000054 or KBio2-001321 or KBio2-003889 or KBio2-006457 or KBioGR-001253 or KBioSS-001321 or Lopac-F-6627 or Lopac0-000536 or MFCD00006018 or MLS000069498 or MLS002415705 or NCGC00015442-01 or NCGC00015442-02 or NCGC00015442-03 or NCGC00015442-04 or NCGC00015442-05 or NCGC00015442-06 or NCGC00015442-07 or NCGC00015442-08 or NCGC00015442-09 or NCGC00015442-10 or NCGC00015442-11 or NCGC00015442-12 or NCGC00015442-15 or NCGC00015442-16 or NSC-19893 or NSC757036 or NSC816997 or Phtoruracil$2 or Pharmakon1600-01500305 or Phthoruracil$2 or Queroplex$2 or Ro-2-9757 or S1209 or 191047-64-0 or 191047-65-1 or 191115-88-5 or U3P01618RT or 51-21-8).ti,ab,kw,kf,ot,hw,rn,nm. [FLUOROURACIL TERMS] | 250857 |
| 158 | Irinotecan/ or (irinotecan$ or ab00698464-07 or ab00698464-09 or ab00698464-10 or ab00698464-11 or ab00698464-12 or ab00698464-13 or ab00698464-14 or ac-7469 or akos015894969 or amy4227 or as-14323 or bdbm50128267 or bcp02860 or bcp9000793 or biotecan$2 or brd-k08547377-003-02-4 or campto$2 or camptosar$2 or chebi-80630 or chembl481 or cs-1138 or cpt-11 or cpt11 or d08086 or db00762 or dq2805 or en300-708800 or gtpl6823 or hsdb-7607 or ihl-305 or ihl305 or irinophore-c$2 or irinotel$2 or mfcd00866307 or ncgc00178697-02 or ncgc00178697-05 or nsc-728073 or nsc728073 or nk012-compound or q412197 or s1198 or schembl4034 or sn38 or sn-38 or sn-38-11 or sn3811 or topotecin$2 or u-101440e or u101440e or zinc1612996 or "7673326042" or 100286-90-6 or 97682-44-5).ti,ab,kw,kf,ot,hw,rn,nm. [IRINOTECAN TERMS] | 69035 |
| 159 | (teysuno$2 or (tegafur adj4 gimeracil adj4 oteracil) or ((S-1 or S1) adj3 combination) or TS-1-cpd or S-1-cpd or TS-1 or TS1 or BMS247616 or BMS-247616 or S1-tegafur-oxonate or S1-fluoropyrimidine-oxoonate).ti,ab,kw,kf,ot,hw,rn,nm. [S1 COMBINATION TERMS] | 11045 |
| 160 | Tegafur/ or (1189456-27-6 or 1548r74nsz or 17902-23-7 or 82294-77-7 or a812417 or ab00572620-15 or ac-2112 or akos000121279 or as-13528 or atillon$2 or bcp22714 or bp-58663 or brn-0525766 or c8h9fn2o3 or ccg-100959 or ccg-50110 or ccris-2762 or chebi-32188 or chembl20883 or citofur$2 or coparogin$2 or cs-1128 or d01244 or db09256 or dtxsid001009966 or einecs-241-846-2 or en300-21668 or exonal$2 or f-5-fu or fental$2 or florafur$2 or fluorafur$2 or fluorofur$2 or franrose$2 or franroze$2 or ft-0653732 or ft-0654170 or ft-0674829 or ft-0693965 or ft207 or ft-207 or ftorafur$2 or fulaid$2 or fulfeel$2 or furafluor$2 or furflucil$2 or furofutran$2 or futraful$2 or gtpl10513 or hms1665i05 or hms2051b15 or hms2090k04 or hms2232e05 or hms3371h21 or hms3393b15 or hms3654p13 or hms3715d14 or hy-17400 or lamar$2 or lifril$2 or mfcd00012351 or mjf12264 or mjf-12264 or mls000069497 or mls000759414 or mls001076521 or mls001424119 or nc00209 or ncgc00159418-02 or ncgc00159418-04 or ncgc00159418-05 or neberk$2 or nitobanil$2 or nsc148958 or nsc-148958 or opera-id-1726 or phthorafur$2 or q-201784 or q413370 or racemic-ftorafur or riol$2 or schembl4552 or sfsp$2 or sf-sp or sinoflurol$2 or smr000059106 or sr-01000639511 or sr-01000639511-1 or sr-01000639511-4 or sunfral$2 or sunfural$2 or tefsiel-c or tegaful$2 or tegafur$2 or tegafurum$2 or ts-1 or uftoral$2 or upcmld-dp063 or utefos$2 or z104508106).ti,ab,kw,kf,ot,hw,rn,nm. [TEGAFUR TERMS] | 28565 |
| 161 | Ramucirumab/ or (1121b or 947687-13-0 or 947687-13-0 or a168 or a-168 or cyramza$2 or d99yvk4l0x or hlx12 or hlx-12 or hsdb-8314 or imc1121b or imc1121-b or imc-1121b or imc-1121-b or l01xc21 or ly3009806 or ly-3009806 or nsc-749128 or pbp2001 or pbp-2001 or ramucirumab$2 or ramucirumabum$2 or ro7234952 or ro-7234952).ti,ab,kw,kf,ot,hw,rn,nm. [RAMUCIRUMAB TERMS] | 8955 |
| 162 | Panitumumab/ or (339177-26-3 or 6a901e312a or abenix$2 or abx-egf or amg954 or amg-954 or e7-6-3 or l01xc08 or moab-abx-egf or moab-e7-6-3 or monoclonal-antibody-abx-egf or monoclonal-antibody-e7-6-3 or nsc-742319 or panitumab$2 or panitumumab$2 or panitumumabum$2 or panitunumab$2 or vectibex$2 or vectibix$2).ti,ab,kw,kf,ot,hw,rn,nm. [PANITUMUMAB TERMS] | 13499 |
| 163 | (6ns400bxkh or 780758-10-3 or 828933-51-3 or biomab-egfr or diacim$2 or h-r3 or nimotuzumab$2 or osag-101 or radiotheracim$2 or theracim$2 or theraloc$2).ti,ab,kw,kf,ot,hw,rn,nm. [NIMOTUZUMAB TERMS] | 2524 |
| 164 | (339186-68-4 or emd7200 or emd-7200 or emd72000 or emd-72000 or kgaa$2 or matuzumab$2 or merck-kgaa or mg4m3qb242).ti,ab,kw,kf,ot,hw,rn,nm. [MATUZUMAB TERMS] | 51359 |
| 165 | Bevacizumab/ or (12-igg1 or 1438851-35-4 or 216974-75-3 or 2s9zzm9q9v or abevmy$2 or abp215 or abp-215 or ainex$2 or altuzan$2 or alymsys$2 or ankeda$2 or anti-vegf or askb1202 or ask-b1202 or avastin$2 or avegra$2 or aybintio$2 or ba1101 or ba-1101 or bambevi$2 or bat1706 or bat-1706 or bcd021 or bcd-021 or bevacizumab$2 or bevacizumabum$2 or bevagen$2 or bevatas$2 or bevax$2 or bevz92 or bevz-92 or bi695502 or bi-695502 or bow030 or bow-030 or boyounuo$2 or bp01 or bp-01 or bp102 or bp-102 or bryxta$2 or bs503a or bs-503a or bxt2316 or bxt-2316 or byvasda$2 or cbt124 or cbt-124 or chs305 or chs-305 or chs5217 or chs-5217 or cizumab$2 or ctp16 or ct-p16 or equidacent$2 or fkb238 or fkb-238 or gb222 or gb-222 or gbs004 or gbs-004 or hanbeitai$2 or hd204 or hd-204 or hlx04 or hlx-04 or hot1010 or hot-1010 or hsdb-8080 or ibi305 or ibi-305 or idb0072 or idb-0072 or intp24 or intp-24 or ipique$2 or jhl1149 or jhl-1149 or js501 or js-501 or jy028 or jy-028 or krabeva$2 or kyomarc$2 or l01xc07 or lextemy$2 or "lumiere-(drug)" or ly01008 or ly-01008 or mabionvegf$2 or mb02 or mb-02 or mil60 or mil-60 or mvasi$2 or myl14020 or myl-14020 or myl1402o or myl-1402o or nsc704865 or nsc-704865 or onbevzi$2 or ons1045 or ons-1045 or ons5010 or ons-5010 or oyavas$2 or pf06439535 or pf-06439535 or pf6439535 or pf-6439535 or pmc901 or pmc-901 or pobevcy$2 or pro169 or pro-169 or pusintin$2 or ql1101 or ql-1101 or r435 or r-435 or rg435 or rg-435 or rhumab$2 or ro4876646 or ro-4876646 or rph001 or rph-001 or rtpr023 or r-tpr-023 or sb8 or sb-8 or sct501 or sct-501 or sct510 or sct-510 or sibp04 or sibp-04 or stc103 or stc-103 or stivant$2 or tab008 or tab-008 or tab014 or tab-014 or tot102 or tot-102 or trs003 or trs-003 or tx16 or tx-16 or vegzelma$2 or versavo$2 or zirabev$2 or zrc113 or zrc-113 or zybev$2).ti,ab,kw,kf,ot,hw,rn,nm. [BEVACIZUMAB TERMS] | 128124 |
| 166 | (2022215-59-2 or anb011 or anb-011 or dostarlimab$2 or gsk4057190 or gsk-4057190 or jemperli$2 or p0gvq9a4s5 or tsr042 or tsr-042 or wbp285 or wbp-285).ti,ab,kw,kf,ot,hw,rn,nm. [DOSTARLIMAB TERMS] | 925 |
| 167 | (chembl5095383 or retlirafusp-alfa or shr1701 or shr-1701).ti,ab,kw,kf,ot,hw,rn,nm. [SHR-1701 TERMS] | 56 |
| 168 | (2368219-35-4 or 45x7ou8c4j or ab154 or ab-154 or domvanalimab$2 or who-11559).ti,ab,kw,kf,ot,hw,rn,nm. [DOMVANALIMAB TERMS] | 101 |
| 169 | (2259860-24-5 or ab122 or ab-122 or gls010 or gls-010 or gs0122 or gs-0122 or wbp3055 or wbp-3055 or who-11413 or zbl7o904il or zimberelimab$2).ti,ab,kw,kf,ot,hw,rn,nm. [ZIMBERELIMAB TERMS] | 225 |
| 170 | Lapatinib/ or (0vua21238f or 1092929-10-6 or 1210608-87-9 or 1xkk or 231277-92-2 or 388082-78-8 or 437755-78-7 or 913989-15-8 or a25184 or ab01273965-01 or ab01273965-02 or ab01273965-03 or ab01273965-04 or ab01273965-05 or ac-1314 or akos005145766 or am20090641 or as-14065 or bc164610 or bcp01874 or bcp9000837 or bcp9000838 or bcpp000188 or bcpp000189 or bdbm5445 or brd-k19687926-001-01-7 or brd-k19687926-379-02-5 or c29h26clfn4o4s or ccg-270133 or chebi-49603 or chembl554 or cid-208908 or d08108 or db01259 or dtxcid5026675 or dtxsid7046675 or en300-117254 or ex-a402 or fmm$2 or ft-0659650 or gsk572016 or gsk-572016 or gtpl5692 or gw2016 or gw-2016 or gw282974x or gw-282974x or gw572016 or gw-572016 or gw572016f or gw-572016f or gw-572016x or hms2089h10 or hms3244n06 or hms3244n10 or hms3244n14 or hms3744k11 or hsdb-8209 or hy-50898 or kinome-3684 or kinome-3685 or l0360 or lapatinib$2 or mfcd09264194 or ncgc00167507-01 or ncgc00167507-02 or ncgc00167507-03 or ncgc00167507-04 or ncgc00167507-09 or ns00003012 or nsc745750 or nsc-745750 or nsc800780 or nsc-800780 or q-101353 or q420323 or sb16918 or schembl8100 or sr-05000001472-1 or sw199101-5 or tox21-112505 or tykerb$2 or tyverb$2).ti,ab,kw,kf,ot,hw,rn,nm. [LAPATINIB TERMS] | 21635 |
| 171 | (857890-39-2 or a825653 or ac-25047 or aiv007 or aiv-007 or akos025401742 or amy9240 or as-16203 or bcp01799 or bcp9000633 or bcpp000247 or bdbm50331094 or bl164616 or c21h19cln4o4 or ccg-264842 or chebi-85994 or chembl1289601 or cs-0109 or d09919 or db09078 or dtxcid50117096 or dtxsid50194605 or e7080 or e-7080 or ee083865g2 or en300-7418350 or er203492-00 or er-203492-00 or ex-a249 or ft-0700727 or gtpl7426 or hms3244a07 or hms3244a08 or hms3244b07 or hms3654a14 or hy-10981 or j-513372 or kisplyx$2 or l01xe29 or lenvatinib$2 or lenvatinibum$2 or lenvima$2 or lev$2 or mfcd16038644 or mk7902 or mk-7902 or mls006011239 or ncgc00263198-01 or ncgc00263198-04 or ncgc00263198-07 or ns00069283 or nsc755980 or nsc-755980 or nsc800781 or nsc-800781 or q6523413 or ro7071618 or ro-7071618 or s1164 or sb16580 or schembl864638 or smr004702999 or sw219259-1 or z2235801899).ti,ab,kw,kf,ot,hw,rn,nm. [LENVATINIB TERMS] | 8193324 |
| 172 | (0rf or 1001264-89-6 or 524y3ib4hq or ac-28420 or akos025396463 or as-17027 or bcp0726000195 or bcp9000712 or bdbm50398379 or ccg-269312 or chebi-95089 or chembl2177390 or cs-0975 or d10641 or db11743 or dtxsid101025595 or ex-a2077 or gdc0068 or gdc-0068 or gdc0068-di-hcl or gdc-0068-di-hcl or gtpl7887 or hy-15186 or ipatasertib$2 or mfcd22124514 or ncgc00346714-01 or ns00072927 or nsc767898 or nsc-767898 or nsc781451 or nsc-781451 or nsc800986 or nsc-800986 or nsc832484 or nsc-832484 or q27078088 or rg7440 or rg-7440 or rg-7440-di-hcl or s2808 or schembl191659).ti,ab,kw,kf,ot,hw,rn,nm. [IPATASERTIB TERMS] | 1190 |
| 173 | or/120-160 [INTERVENTION & COMPARATORS & CHEMO TERMS] | 3286481 |
| 174 | (("randomized controlled trial" or "controlled clinical trial").pt. or (randomized or placebo or randomly).ti,ab. or "clinical trials as topic".sh. or trial.ti.) not (exp animals/ not humans.sh.) [RCTs Filter MEDLINE – Balanced, sensitive vs precise - Cochrane Handbook] | 2886608 |
| 175 | 119 and 173 and 174 | 4590 |
| 176 | (Adolescent/ or exp Child/ or exp Infant/) not (exp Adult/ and (Adolescent/ or exp Child/ or exp Infant/)) [CHILDREN <19 REMOVE] | 4816395 |
| 177 | exp Animals/ not (exp Animals/ and Humans/) [ANIMAL STUDIES ONLY - REMOVE - MEDLINE] | 16930785 |
| 178 | (address or autobiography or bibliography or biography or comment or dictionary or directory or editorial or "expression of concern" or festschrift or historical article or interactive tutorial or lecture or legal case or legislation or news or newspaper article or patient education handout or personal narrative or portrait or video-audio media or webcast or (letter not (letter and randomized controlled trial))).pt. [Opinion publications - Remove -MEDLINE] | 5010919 |
| 179 | Clinical Trial Protocol.pt. | 484683 |
| 180 | 175 not (176 or 177 or 178 or 179) [CHILD <19, ANIMAL STUDIES, TRIAL PROTOCOLS and OPINION PUBLICATIONS - REMOVED - MEDLINE] | 4022 |
| 181 | 180 use ppez [MEDLINE results] | 2029 |
| 182 | exp stomach carcinoma/ or stomach cancer/ or ((esophagus carcinoma/ or esophageal adenocarcinoma/) and exp gastroesophageal junction/) | 269533 |
| 183 | local metastasis/ or metastasis/ or cancer recurrence/ or advanced cancer/ | 841798 |
| 184 | ((((stomach? or gastric$ or cardia or cardiac or antrum? or antral$ or fundus$ or pyloric$ or pylorus$ or ventricul$ or linitis plastica or leather-bottle or ((stomach? or gastric$) and (GC or GEJ))) adj3 (neoplas$ or cancer$ or tumo?r$ or carcinoma$ or malignan$ or oncolog$ or adenocancer$ or adeno-cancer$ or adenoma$ or adenocarcinoma$ or adeno-carcinoma$ or blastoma$ or carcinosarcoma$ or carcino-sarcoma$ or adenoacanthoma$ or adeno-acanthoma$ or epithelioma$ or melanoma$ or mesenchymoma$ or sarcoma$ or thymoma$ or granuloma$ or choriocarcinoma$ or cancerogenes?s or carcinoid$)) or ((stomach? or gastric$) adj3 SCC) or ((esophag$ or oesophag$ or esophagogastric$ or esophago-gastric$ or oesophagogastric$ or oesophago-gastric$ or gastroesophageal$ or gastro-esophageal$ or gastrooesophageal$ or gastro-oesophageal$ or cardio-esophageal$ or cardio-oesophageal$ or cardioesophageal$ or cardiooesophageal$ or EG or GE) adj3 (junction$ or sphincter$) adj3 (neoplas$ or cancer$ or tumo?r$ or carcinoma$ or malignan$ or oncolog$ or adenocancer$ or adeno-cancer$ or adenoma$ or adenocarcinoma$ or adeno-carcinoma$ or blastoma$ or carcinosarcoma$ or carcino-sarcoma$ or adenoacanthoma$ or adeno-acanthoma$ or epithelioma$ or melanoma$ or mesenchymoma$ or sarcoma$ or thymoma$ or granuloma$ or choriocarcinoma$ or cancerogenes?s or carcinoid$))) adj4 ((meta adj sta$) or metastas$ or metastatic$ or recur$ or secondar$ or relaps$ or advance$ or inoperab$ or disseminat$ or spread or migration or lethal$ or incurable or noncurable or non-curable or uncurable or progressive or terminal or invasive$ or aggressive$ or (late? adj2 stage$) or ((stage? or grade? or type?) adj2 (3a$ or 3b$ or 3c$ or III$ or 4a$ or 4b$ or IV$)) or "stage 3" or "stage 4" or met or mets or N1? or N2? or N3? or pN1? or pN2? or pN3?)).ti,ab,kw,kf. [Metastatic GC/GEJ TERMS] | 71738 |
| 185 | (182 and 183) or 184 [GC-GEJ TERMS] | 87902 |
| 186 | tislelizumab/ or (tislelizumab$2 or tirelizumab$2 or bgb-a317 or bgba317 or bgn-1 or bgn1 or jhl-2108 or jhl2108 or vdt-482 or vdt482 or 1858168-59-8 or 0kvo411b3n).ti,ab,kw,kf,ot,rn,dq. [TISLELIZUMAB TERMS] | 2437 |
| 187 | immune checkpoint inhibitor/ or ((programmed death 1 receptor/ or programmed death 1 ligand 2/) and (inhibit$ or block?).ti,ab,kw,kf.) or ((immune$ adj3 checkpoint? adj3 (inhibit$ or block?)) or (((programmed adj3 death) or PD-1 or PD-1-PD-L1 or PDCD1) adj3 (ligand? or inhibit$ or block?)) or ((B7-H1 or B7H1 or "B7 homolog 1" or CD274 or CD273 or PDCD1LG1 or PDCD1LG2) adj3 (antigen? or protein?)) or ((Cytotoxic-T-Lymphocyte-Associated Protein-4 Inhibitor? or CTLA-4) adj3 (inhibit$ or block?)) or (ICI? and "Immun$ Checkpoint") or BMS-1 or EX-A947 or HY-19991 or J-690233 or MFCD28978741 or s7911 or D000082082 or SCHEMBL16555159 or ZINC230477930 or 1675201-83-8).ti,ab,kw,kf,ot,rn,dq. [IMMUNE CHECKPOINT PROTEINS TERMS] | 139900 |
| 188 | cancer immunotherapy/ or monoclonal antibody/ or (immunotherap$ or immuno-therap$ or (((biologic$ adj3 response? adj3 modifier?) or BRM or immunogenic$ or immunologic$ or immuno-genic$ or immuno-logic$ or radioimmunotherapy$ or radio-immunotherap$ or ((monoclonal$ or clonal$ or hybridoma$) adj2 antibod$)) adj3 (therap$ or intervention? or treat$))).ti,ab,kw,kf. [IMMUNOTHERAPY TERMS] | 885815 |
| 189 | molecularly targeted therapy/ or ((molecular$ or neoplas$ or cancer$ or tumo?r$ or carcinoma$) adj3 target$ adj3 therap$).ti,ab,kw,kf. [TARGETED THERAPY TERMS] | 165564 |
| 190 | atezolizumab/ or (atezolizumab$2 or anti-PDL1 or MPDL-3280A or MPDL3280A or RG-7446 or RG7446 or ro-5541267 or ro5541267 or Tecentriq$2 or Tecntriq$2 or 1380723-44-3 or 0INE2SFD9E or 52CMI0WC3Y).ti,ab,kw,kf,ot,rn,dq. [ATEZOLIZUMAB TERMS] | 23465 |
| 191 | avelumab/ or (avelumab$2 or bavencio$2 or msb-0010682 or msb-0010718c or msb0010682 or msb0010718c or msb-10682 or msb-10718c or msb10682 or msb10718c or pf-06834635 or pf-6834635 or pf06834635 or pf6834635 or KXG2PJ551I or 1537032-82-8).ti,ab,kw,kf,ot,rn,dq. [AVELUMAB TERMS] | 8382 |
| 192 | camrelizumab/ or (camrelizumab$2 or "anti-pd-1 monoclonal antibody" or shr-1210 or shr1210 or carilizumab$2 or carrelizumab$2 or 73096E137E or 1798286-48-2).ti,ab,kw,kf,ot,rn,dq. [CAMRELIZUMAB TERMS] | 4463 |
| 193 | durvalumab/ or (1428935-60-7 or 28x28x9okv or anti-b7h1-monoclonal-antibody or durvalumab$2 or durvalumabum$2 or imfinzi$2 or l01xc28 or medi4736 or medi-4736).tw,kw,kf,ot,rn,dq. [DURVALUMAB TERMS] | 14684 |
| 194 | ipilimumab/ or (ipilimumab$2 or bms-734016 or bms734016 or cs-1002 or cs1002 or ibi-310 or ibi310 or mdx-ctla-4 or mdx-010 or mdx-101 or mdx010 or mdx101 or strentarga$2 or yervoy$2 or 6T8C155666 or 477202-00-9).ti,ab,kw,kf,ot,rn,dq. [IPILIMUMAB TERMS] | 34719 |
| 195 | nivolumab/ or (nivolumab$2 or bms-936558 or bms-986213 or bms-986298 or cmab819 or bms936558 or bms986213 or bms986298 or cmab-819 or mdx-1106 or mdx1106 or ono-4538 or ono4538 or opdivo$2 or opdualag$2 or 31YO63LBSN or 946414-94-4).ti,ab,kw,kf,ot,rn,dq. [NIVOLUMAB TERMS] | 55402 |
| 196 | pembrolizumab/ or (pembrolizumab$2 or keytruda$2 or lambrolizumab$2 or mk3475 or mk-1308a or mk-3475 or mk7684a or sch-900475 or sch900475 or "keylynk-010 component" or DPT0O3T46P or 1422183-02-5 or 1374853-91-4).ti,ab,kw,kf,ot,rn,dq. [PEMBROLIZUMAB TERMS] | 54538 |
| 197 | sintilimab/ or (2072873-06-2 or 8fu7fq8upk or ibi308 or ibi-308 or sintilimab$2 or tyvyt$2 or who-10801).tw,kw,kf,ot,rn,dq. [SINTILIMAB TERMS] | 3049 |
| 198 | toripalimab/ or (1924598-82-2 or 8jxn261vva or js001 or js-001 or tab001 or tab-001 or teripalimab$2 or toripalimab$2 or treipril$2 or treprizumab$2 or tripleitriumab$2 or triprizumab$2 or tuoyi$2 or who-10820).tw,kw,kf,ot,rn,dq. [TORIPALIMAB TERMS] | 2186 |
| 199 | retifanlimab/ or (2079108-44-2 or 2226345-85-1 or 2y3t5if01z or aex1188 or aex-1188 or incmga00012 or incmga-00012 or incmga0012 or incmga-0012 or mga012 or mga-012 or retifanlimab$2 or zynyz$2).tw,kw,kf,ot,rn,dq. [RETIFANLIMAB TERMS] | 239 |
| 200 | envafolimab/ or (2102192-68-5 or anti-pd-l1-monoclonal-antibody-kn035 or asc22 or asc-22 or envafolimab$2 or es1m06m6qh or kn035 or kn-035).tw,kw,kf,ot,rn,dq. [ENVAFOLIMAB TERMS] | 200 |
| 201 | tebotelimab/ or (2245725-04-4 or l62556gpxb or mgd013 or mgd-013 or tebotelimab$2).tw,kw,kf,ot,rn,dq. [TEBOTELIMAB TERMS] | 99 |
| 202 | cadonilimab/ or (2394841-59-7 or 6fyg1ds4nw or ak104 or ak-104 or cadonilimab$2 or who-11581).tw,kw,kf,ot,rn,dq. [CADONILIMAB TERMS] | 155 |
| 203 | serplulimab/ or (2231029-82-4 or hlx10 or hlx-10 or s3gqz2k36v or serplulimab$2).tw,kw,kf,ot,rn,dq. [SERPLULIMAB TERMS] | 186 |
| 204 | sugemalimab/ or (2256084-03-2 or 90iqr2i6tr or cs1001 or cs-1001 or sugemalimab$2 or wbp315 or wbp-315 or wbp3155 or wbp-3155).tw,kw,kf,ot,rn,dq. [SUGEMALIMAB TERMS] | 199 |
| 205 | zolbetuximab/ or (1496553-00-4 or claudiximab$2 or imab362 or imab-362 or tf5mpq8wgy or zolbetuximab$2).tw,kw,kf,ot,rn,dq. [ZOLBETUXIMAB TERMS] | 317 |
| 206 | bemarituzumab/ or (1952272-74-0 or bemarituzumab$2 or fpa144 or fpa-144 or rjw23bq0kw).tw,kw,kf,ot,rn,dq. [BEMARITUZUMAB TERMS] | 129 |
| 207 | cetuximab/ or (205923-56-4 or abp494 or abp-494 or c225 or c-225 or c225-03 or c-22503 or c-225-03 or cdp1 or cdp-1 or cetuximab$2 or cetuximabum$2 or ch225 or ch-225 or chimeric-anti-egfr-monoclonal-antibody or cmab009 or cmab-009 or ctp15 or ct-p15 or dtxsid0040830 or erbitux$2 or hsdb-7454 or imc225 or imc-225 or imcc225 or imcc-225 or imc-c225 or kl140 or kl-140 or l01xc06 or ly2939777 or ly-2939777 or mab-c225 or moab-c225 or nsc714692 or pqx0d8j21j or sti001 or sti-001).tw,kw,kf,ot,rn,dq. [CETUXIMAB TERMS] | 48550 |
| 208 | onartuzumab/ or (1133766-06-9 or metmab$2 or metma-b or ms1j9720wc or onartuzumab$2 or pro143966 or pro-143966 or pro-143996 or pro143996 or ro5490258 or ro-5490258).tw,kw,kf,ot,rn,dq. [ONARTUZUMAB TERMS] | 776 |
| 209 | rilotumumab/ or (51wew898ij or 872514-65-3 or amg102 or amg-102 or rilotumumab$2).tw,kw,kf,ot,rn,dq. [RILOTUMUMAB TERMS] | 725 |
| 210 | andecaliximab/ or (1518996-49-0 or 571045eim4 or andecaliximab$2 or gs5745 or gs-5745).tw,kw,kf,ot,rn,dq. [ANDECALIXIMAB TERMS] | 188 |
| 211 | pazopanib/ or (444731-52-6 or 635702-64-6 or 790713-33-6 or 7rn5dr86ck or a19406 or a839572 or ab01273967-01 or ab01273967-02 or ab01273967-05 or ab01273967-06 or ac-8522 or akos005145819 or am20090659 or ar-270-43507999 or armala$2 or as-11066 or bcp01839 or bcp9001053 or bcpp000129 or bd164238 or bdbm26474 or brd-k74514084-003-02-7 or ccg-265010 or chebi-71219 or chembl477772 or cid-10113978 or cs-0269 or db06589 or dtxcid1028659 or dtxsid8048733 or en300-57325 or ex-a1241 or ft-0659928 or ft-0684794 or gtpl5698 or gw780604 or gw-780604 or gw-78603 or gw786034 or gw7-86034 or gw-786034 or gw786034b or gw-786034b or gw786034x or gw-786034x or hms3244c21 or hms3244c22 or hms3244d21 or hms3656l14 or hms3745g05 or hsdb-8210 or hy-10208 or indazolylpyrimidine-13 or jmc514632-compound-13 or kinome-3790 or mfcd11616589 or ncgc00188865-01 or ncgc00188865-02 or ncgc00188865-03 or ncgc00188865-10 or nsc752782 or nsc-752782 or nsc800839 or nsc-800839 or p-6706 or pazopanib$2 or pazopanibum$2 or q-101400 or q7157043 or s3012 or sb17290 or sb710468 or sb-710468 or sb710468a or sb-710468a or schembl588608 or sw218082-3 or tox21-113174 or tox21-113174-1 or votrient$2 or z1541638525).tw,kw,kf,ot,rn,dq. [PAZOPANIB TERMS] | 14624 |
| 212 | rivoceranib/ or (1218779-75-9 or 5s371k6132 or 811803-05-1 or ab01274807-01 or ab01274807-02 or ac-27461 or akos024464453 or amy21302 or apatinib$2 or ba175030 or bcp02840 or c76598 or ccg-268625 or chembl3186534 or cs-0003200 or d11288 or db14765 or ds-7455 or dtxsid601024366 or ex-a1794 or gtpl7648 or hy-13342a or mfcd21648511 or ncgc00249393-01 or ncgc00249393-08 or nsc772886 or nsc-772886 or nsc799333 or nsc-799333 or q27262801 or rivoceranib$2 or s5248 or sb16590 or schembl1814966 or yn968d1 or yn-968d1).tw,kw,kf,ot,rn,dq. [RIVOCERANIB/APATINIB TERMS] | 4983 |
| 213 | exp cancer chemotherapy/ or (chemotherap$ or chemo-therap$ or carcinochemotherap$ or chemoradiotherap$ or chemoradiation? or radiochemotherap$ or carcino-chemotherap$ or chemo-radiotherap$ or chemo-radiation? or radio-chemotherap$).ti,ab,kw,kf. [CHEMOTHERAPY TERMS] | 1743405 |
| 214 | folinic acid/ or (leucovorin$ or 6-s-leucovorin or 6s-leucovorin or acide folinique or akos015961207 or bdbm50039121 or bpbio1-000766 or bspbio-000696 or bspbio-002218 or brd-a75919782-238-01-8 or calcium folinate or chebi-15640 or chembl1679 or chembl69905 or citrovoeum-factor or citrovorum-factor or d93089 or divk1c-000222 or dtxsid0048216 or einecs-200-361-6 or en300-27068710 or folinate folinic-acid-sf or folinic acid or formyltetrahydrofolate or fusilev$2 or gtpl4816 or gtpl6690 or hsdb-6544 or hy-17556 or idi1-000222 or kbio1-000222 or kbio2-001339 or kbio2-003907 or kbio2-006475 or kbio3-001438 or kbiogr-000461 or kbioss-001339 or lencovorin$2 or leucal$2 or leukovorin$2 or leukovoran$2 or leucovorin$2 or levoleucovorin$2 or levo-leucovorin$2 or mfcd00867488 or ninds-000222 or nsc3590 or prestwick0-000738 or prestwick1-000738 or prestwick2-000738 or prestwick3-000738 or q45435667 or q573i9dvlp or s5790 or schembl10068238 or schembl8349 or sd-204098 or s-leucovorin$2 or sleucovorin$2 or spectrum2-000116 or spectrum3-000479 or spectrum4-000031 or spectrum5-000910 or spectrum-000859 or spbio-000132 or spbio-002635 or sbi-0051427-p003 or welcovorin$2 or "formyltetrahydropteroylglutamic acid" or Q573I9DVLP or 58-05-9).ti,ab,kw,kf,ot,rn,dq. [LEUCOVORIN TERMS] | 68089 |
| 215 | carboplatin/ or (carboplatin$2 or blastocarb$2 or boplatex$2 or carboplat$ or carbosin$2 or carbotec$2 or carplan$2 or CBDCA or (platinum adj3 (cis or diamin? or cyclobutanedicarboxylat? or dicarboxylatediammine)) or Dicarboxylatoplatinum or diamminecyclobutanedicarboxylatoplatinum or cycloplatin$ or erbakar$2 or ercar$2 or ifacap$2 or jm8 or jm-8 or kemocarb$2 or nsc-241240 or nsc241240 or nsc-201345 or nsc201345 or oncocarbin$2 or paraplatin$ or Platinwas$2 or Ribocarbo$2 or Neocarbo$2 or Nealorin$2 or HSDB-6957 or BG3F62OND5 or 41575-94-4).ti,ab,kw,kf,ot,rn,dq. [CARBOPLATIN TERMS] | 129154 |
| 216 | exp paclitaxel/ or (paclitaxel$ or abraxane$2 or abraxus$2 or act02709 or act-02709 or acon1-002231 or anx-513 or anzatax$2 or apealea$2 or asotax$2 or bidd-pxr0046 or biotax$2 or bms-181339 or bms181339-01 or bms181339 or bms-181339-01 or bmy-45622 or bmy45622 or bspbio-000290 or capxol$2 or ccris-8143 or chembl428647 or chebi-45863 or coroflex-please$2 or coroxane$2 or cmap-000068 or cynviloq$2 or cypher-select$2 or dsstox-cid-3413 or dsstox-gsid-23413 or dsstox-rid-77016 or dhp107 or dhp-107 or dhp-208 or dhp208 or dts-301 or dts301 or ebetaxel$2 or empac$2 or endotag-1 or endotag1 or formoxol$2 or genaxol$2 or genetaxyl$2 or genexol$2 or gtpl2770 or hms2090d07 or hms2095o12 or hms2231a16 or hms3712o12 or hsdb-6839 or hunxol$2 or hy-b0015 or ifaxol$2 or ig-001 or ig001 or infinnium$2 or intaxel$2 or kbiogr-002509 or kbio2-002509 or kbio2-005077 or kbio2-007645 or kbio3-002987 or lep-etu$2 or lipopac$2 or liporaxel$2 or mbt-0206 or mbt0206 or medixel or mfcd00869953 or mitotax$2 or nanopac$2 or nanotax$2 or nanotaxel$2 or ncgc00164367-01 or nk-105 or nk105 or nsc-125973 or nsc-673089 or nsc125973 or nsc673089 or nscc-125973 or nova-12005 or oas-pac-100 or oaspac100 or oncogel$2 or onxal$2 or onxol$2 or p-ssmm-vip$2 or paclical$2 or pacitaxel$2 or paclical$2 or padexol$2 or pacligel$2 or paclitaxel$2 or pacliex$2 or paxceed$2 or paxene$2 or paxoral$2 or paxus$2 or pazenir$2 or plaxicel$2 or praxel$2 or qw-8184 or schembl3976 or sb-05 or sb05 or sdp-013 or sindaxel$2 or smr000857385 or sr-01000075350 or taycovit$2 or taxalbin$2 or taxane$ or taxocris$2 or taxol$2 or taxus$2 or tocosol$2 or xorane$2 or yewtaxan$2 or zinc96006020 or zisu$2 or P88XT4IS4D or 33069-62-4).ti,ab,kw,kf,ot,rn,dq. [PACLITAXEL TERMS] | 230999 |
| 217 | docetaxel/ or (114915-20-7 or 114977-28-5 or 15h5577cqd or 699121phca or ab01273941-01 or ab01273941-02 or ac-383 or akos015960718 or akos024457953 or amy4356 or anx-514 or axtere$2 or bd164373 or bdbm36351 or bind014 or bind-014 or brd-k30577245-001-04-3 or brd-k30577245-341-01-9 or bs102 or bs-102 or chebi-4672 or chembl92 or cid148124 or ckd-810 or crlx301 or crlx-301 or cs-1144 or d07866 or d4102 or daxotel$2 or db01248 or dexotel$2 or docecad$2 or docefrez$2 or docetaxel$2 or docetaxelum$2 or docetaxol$2 or docetaxolum$2 or dtxcid8020464 or dtxsid0040464 or emdoc$2 or en300-123047 or ex-a1206 or gtpl6809 or hms2089k08 or hsdb-6965 or hy-b0011 or ks-1452 or l01cd02 or lit976 or lit-976 or mfcd00871399 or ncgc00181306-01 or ncgc00181306-02 or ncgc00181306-04 or ncgc00242509-01 or nsc628503 or nsc-628503 or nsc-759850 or oncodocel$2 or q-100074 or q420436 or rp56976 or rp-56976 or schembl4419 or sdp-014 or sid-530 or sr-01000003023 or sr-01000003023-5 or syp-0704a or taxanit$2 or taxespira$2 or taxoel$2 or taxoltere-metro or taxotel$2 or taxoter$2 or taxotere$2 or texot$2 or tox21-112781 or tox21-113088 or txl$2 or w-60384 or xrp6976 or xrp-6976 or xrp-6976l or z1546621742).tw,kw,kf,ot,rn,dq. [DOCETAXEL TERMS] | 107752 |
| 218 | cisplatin/ or (cisplatin$ or platinum$ or cismaplat$2 or (cis adj3 ($platinum or platinous)) or cis-platinum or cis-Platin or dichloroplatinum or diaminodichloroplatinum or diamminedichloroplatinum or dichlorodiammineplatinum or AI3-62048 or abiplatin or biocisplatinum or biocysplatinum or blastolem$2 or briplatin$2 or cddp-ti or cis-ddp or cisPt$ or CACP or CCRIS-221 or CDDP or DDPt or CP-Ethypharm or CPDC or CPDD or CPPD or (DDP and antitumor) or cisplatyl$2 or citoplatino$2 or cytoplatin$2 or cytosplat$2 or docistin$2 or elvecis$2 or kemoplat$2 or Fauldiscipla$2 or IA-call or LiPlaCis$2 or lederplatin$2 or lipoplatin$2 or "liposomal cisplatin" or mpi-5010 or mpi5010 or neoplatin$2 or niyaplat$2 or nk-801 or noveldexis$2 or nsc-119875 or nci-c55776 or platamine$2 or platamine-rtu or platiblastin$2 or platidiam$2 or platimine$2 or platinex$2 or platinil$2 or platino$2 or platinol$2 or platinolaq$2 or platinol-aq$2 or platinoxan$2 or platiran$2 or platistil$2 or platistin$2 or platosin$2 or "pronto platamine" or "Peyrone's chloride" or randa$2 or romcis$2 or sicatem$2 or spi-077 or tr-170 or tecnoplatin$2 or Q20Q21Q62J or 15663-27-1 or 26035-31-4 or 96081-74-2).ti,ab,kw,kf,ot,rn,dq. [CISPLATIN TERMS] | 462622 |
| 219 | oxaliplatin/ or (oxaliplatin$2 or (oxalat$ adj3 platin$) or axiplatin$2 or bendaplatin$2 or crisapla$2 or croloxat$2 or dacotin$2 or dacplat$2 or ebeoxal$2 or elatofen$2 or eloxatin$ or elplat$2 or euroxaliplatin$2 or geneplatin$2 or gessedil$2 or heloxatin$2 or lipoxal$2 or mbp-426 or mbp426 or medoxa$2 or oksaliplatin$ or oplat$2 or oxalatoplatin$ or oxalatplatin$2 or oxali$2 or oxalip$2 or oxaliplan$2 or oxaliprol$2 or oxaliquid$2 or oxalisan$2 or oxalisin$2 or oxalizor$2 or oxaltic$2 or oxaltin$2 or oxamed$2 or oxaplamyl$2 or oxaviatin$2 or platox$2 or plaxitin$2 or rectoxal$2 or riboxatin$2 or rp-54780 or rp54780 or sinoxal$2 or sr-96669 or sr96669 or transplastin$2 or velminox$2 or xaliplat$2 or xoplan$2 or L-OHP-Cpd or 1-OHP or ACT-078 or ACT078 or CCRIS-9143 or NSC-266046 or 04ZR38536J or 61825-94-3).ti,ab,kw,kf,ot,rn,dq. [OXALIPLATIN TERMS] | 91248 |
| 220 | capecitabine/ or (capecitabin$ or apecitab$2 or atubri$2 or bc164277 or bcpp000300 or bxeliri$2 or bs-1000 or cacit$2 or capcel$2 or capebina$2 or capecite$2 or capegard$2 or capezam$2 or capicet$2 or capiri$2 or capiibine$2 or captabin$2 or capnat$2 or capoda$2 or capostat$2 or capsy$2 or capxcel$2 or caxeta$2 or ccg-264841 or ccx-340 or cpecitabine$2 or cs-0768 or d01223 or db01101 or dsstox-cid-26451 or dsstox-gsid-46451 or dsstox-rid-81625 or dtxsid3046451 or ecansya$2 or ex-a835 or gtpl6799 or hsdb-7656 or hy-b0016 or j-700154 or k007 or m0297 or mfcd00930626 or mls003915642 or mls004774137 or ncgc00164569-01 or ncgc00164569-02 or ncgc00164569-05 or nsc-759853 or paxon$2 or q-200788 or q420207 or r-340 or rg-340 or r340 or rg340 or ro-09-1978 or ro-091978 or ro-09-1978 or ro-09-1978-000 or ro091978 or s1156 or s-1156 or sr-01000931255 or tox21-112198 or x-abine$2 or x-tabin$2 or xabine$2 or xecap$2 or xeliri$2 or xelocel$2 or xeloda$2 or xelox$2 or z1501480421 or zinc3806413 or 6804dj8z9u or 154361-50-9 or 958887-39-3).ti,ab,kw,kf,ot,rn,dq. [CAPECITABINE TERMS] | 56604 |
| 221 | (platinum adj1 (fluoropyrimidine or fluoro-pyrimidine) adj3 (doublet? or combin$ or chemotherap$ or chemo-therap$ or ((first or front) adj1 line?) or 1-LOT or 1L or therap$ or regimen? or expos$)).ti,ab,kw,kf,ot,rn,dq. [PLATINUM-FLUOROPYRIMIDINE DOUBLET TERMS] | 254 |
| 222 | fluoropyrimidine/ or fluoropyrimidine derivative/ or (5-fluoropyrimidin$ or 5-fluoro-pyrimidine or pyrimidine-5-fluoro or (fluorinated adj1 pyrimidine) or a9048 or ac-453 or akos006346044 or am86123 or "bb 0260992" or c4h3fn2 or db-007051 or dtxsid80217851 or en300-6966105 or f14737 or ft-0601423 or mfcd06658278 or q42859845 or w-203496 or zinc1845840 or 675f218 or L36X4TD47C or 675-21-8).ti,ab,kw,kf,ot,rn,dq. [FLUOROPYRIMIDINE TERMS] | 8673 |
| 223 | fluorouracil/ or fluorouracil derivative/ or (fluorouracil$ or fluroblastin$ or 1upf or 5-Faracil or 5-Fluoracil or 5-Fluoracyl or 5-fluoro-uracil or 5-fluoro-uracil or 5-Fluoroblastin or 5-fluorouacil or 5-Ftouracyl or 5-FU or 5FU or 5F-uracil or Adrucil$2 or AI3-25297 or Arumel$2 or BSPBio-002048 or Cancersil$2 or Carac$2 or Carzonal$2 or CHEBI-46345 or CHEMBL185 or Cinco-FU or CCRIS-2582 or DSSTox-CID-634 or DSSTox-GSID-20634 or DSSTox-RID-75705 or Efudex$2 or Efudix$2 or Efurix$2 or EINECS-200-085-6 or Effluderm$2 or Fluoroblastin$2 or Fluoro-Uracil$2 or Fluoro-uracile$2 or Fluoro-uracilo$2 or Fluoroplex$2 or Fluorouracile$2 or Fluorouracilo$2 or Fluorouracilum$2 or Fluorouracilum$2 or Fluracil$2 or Fluracilum$2 or Fluri$2 or Fluril$2 or Fluuro-Uracil$2 or Fluorouracilo$2 or Fluroblastin$2 or Fluro-Uracil$2 or Ftoruracil$2 or GTPL4789 or HSDB 3228 or IDI1-000054 or Kecimeton$2 or KBio1-000054 or KBio2-001321 or KBio2-003889 or KBio2-006457 or KBioGR-001253 or KBioSS-001321 or Lopac-F-6627 or Lopac0-000536 or MFCD00006018 or MLS000069498 or MLS002415705 or NCGC00015442-01 or NCGC00015442-02 or NCGC00015442-03 or NCGC00015442-04 or NCGC00015442-05 or NCGC00015442-06 or NCGC00015442-07 or NCGC00015442-08 or NCGC00015442-09 or NCGC00015442-10 or NCGC00015442-11 or NCGC00015442-12 or NCGC00015442-15 or NCGC00015442-16 or NSC-19893 or NSC757036 or NSC816997 or Phtoruracil$2 or Pharmakon1600-01500305 or Phthoruracil$2 or Queroplex$2 or Ro-2-9757 or S1209 or 191047-64-0 or 191047-65-1 or 191115-88-5 or U3P01618RT or 51-21-8).ti,ab,kw,kf,ot,rn,dq. [FLUOROURACIL TERMS] | 250449 |
| 224 | irinotecan/ or (irinotecan$ or ab00698464-07 or ab00698464-09 or ab00698464-10 or ab00698464-11 or ab00698464-12 or ab00698464-13 or ab00698464-14 or ac-7469 or akos015894969 or amy4227 or as-14323 or bdbm50128267 or bcp02860 or bcp9000793 or biotecan$2 or brd-k08547377-003-02-4 or campto$2 or camptosar$2 or chebi-80630 or chembl481 or cs-1138 or cpt-11 or cpt11 or d08086 or db00762 or dq2805 or en300-708800 or gtpl6823 or hsdb-7607 or ihl-305 or ihl305 or irinophore-c$2 or irinotel$2 or mfcd00866307 or ncgc00178697-02 or ncgc00178697-05 or nsc-728073 or nsc728073 or nk012-compound or q412197 or s1198 or schembl4034 or sn38 or sn-38 or sn-38-11 or sn3811 or topotecin$2 or u-101440e or u101440e or zinc1612996 or "7673326042" or 100286-90-6 or 97682-44-5).ti,ab,kw,kf,ot,rn,dq. [IRINOTECAN TERMS] | 68902 |
| 225 | "gimeracil plus oteracil potassium plus tegafur"/ or (teysuno$2 or (tegafur adj4 gimeracil adj4 oteracil) or ((S-1 or S1) adj3 combination) or TS-1-cpd or S-1-cpd or TS-1 or TS1 or BMS247616 or BMS-247616 or S1-tegafur-oxonate or S1-fluoropyrimidine-oxoonate).ti,ab,kw,kf,ot,rn,dq. [S1 COMBINATION TERMS] | 14745 |
| 226 | tegafur/ or (1189456-27-6 or 1548r74nsz or 17902-23-7 or 82294-77-7 or a812417 or ab00572620-15 or ac-2112 or akos000121279 or as-13528 or atillon$2 or bcp22714 or bp-58663 or brn-0525766 or c8h9fn2o3 or ccg-100959 or ccg-50110 or ccris-2762 or chebi-32188 or chembl20883 or citofur$2 or coparogin$2 or cs-1128 or d01244 or db09256 or dtxsid001009966 or einecs-241-846-2 or en300-21668 or exonal$2 or f-5-fu or fental$2 or florafur$2 or fluorafur$2 or fluorofur$2 or franrose$2 or franroze$2 or ft-0653732 or ft-0654170 or ft-0674829 or ft-0693965 or ft207 or ft-207 or ftorafur$2 or fulaid$2 or fulfeel$2 or furafluor$2 or furflucil$2 or furofutran$2 or futraful$2 or gtpl10513 or hms1665i05 or hms2051b15 or hms2090k04 or hms2232e05 or hms3371h21 or hms3393b15 or hms3654p13 or hms3715d14 or hy-17400 or lamar$2 or lifril$2 or mfcd00012351 or mjf12264 or mjf-12264 or mls000069497 or mls000759414 or mls001076521 or mls001424119 or nc00209 or ncgc00159418-02 or ncgc00159418-04 or ncgc00159418-05 or neberk$2 or nitobanil$2 or nsc148958 or nsc-148958 or opera-id-1726 or phthorafur$2 or q-201784 or q413370 or racemic-ftorafur or riol$2 or schembl4552 or sfsp$2 or sf-sp or sinoflurol$2 or smr000059106 or sr-01000639511 or sr-01000639511-1 or sr-01000639511-4 or sunfral$2 or sunfural$2 or tefsiel-c or tegaful$2 or tegafur$2 or tegafurum$2 or ts-1 or uftoral$2 or upcmld-dp063 or utefos$2 or z104508106).tw,kw,kf,ot,rn,dq. [TEGAFUR TERMS] | 21633 |
| 227 | ramucirumab/ or (1121b or 947687-13-0 or 947687-13-0 or a168 or a-168 or cyramza$2 or d99yvk4l0x or hlx12 or hlx-12 or hsdb-8314 or imc1121b or imc1121-b or imc-1121b or imc-1121-b or l01xc21 or ly3009806 or ly-3009806 or nsc-749128 or pbp2001 or pbp-2001 or ramucirumab$2 or ramucirumabum$2 or ro7234952 or ro-7234952).ti,ab,kw,kf,ot,rn,dq. [RAMUCIRUMAB TERMS] | 8951 |
| 228 | panitumumab/ or (339177-26-3 or 6a901e312a or abenix$2 or abx-egf or amg954 or amg-954 or e7-6-3 or l01xc08 or moab-abx-egf or moab-e7-6-3 or monoclonal-antibody-abx-egf or monoclonal-antibody-e7-6-3 or nsc-742319 or panitumab$2 or panitumumab$2 or panitumumabum$2 or panitunumab$2 or vectibex$2 or vectibix$2).ti,ab,kw,kf,ot,rn,dq. [PANITUMUMAB TERMS] | 13491 |
| 229 | nimotuzumab/ or (6ns400bxkh or 780758-10-3 or 828933-51-3 or biomab-egfr or diacim$2 or h-r3 or nimotuzumab$2 or osag-101 or radiotheracim$2 or theracim$2 or theraloc$2).ti,ab,kw,kf,ot,rn,dq. [NIMOTUZUMAB TERMS] | 2521 |
| 230 | matuzumab/ or (339186-68-4 or emd7200 or emd-7200 or emd72000 or emd-72000 or kgaa$2 or matuzumab$2 or merck-kgaa or mg4m3qb242).ti,ab,kw,kf,ot,rn,dq. [MATUZUMAB TERMS] | 51357 |
| 231 | bevacizumab/ or (12-igg1 or 1438851-35-4 or 216974-75-3 or 2s9zzm9q9v or abevmy$2 or abp215 or abp-215 or ainex$2 or altuzan$2 or alymsys$2 or ankeda$2 or anti-vegf or askb1202 or ask-b1202 or avastin$2 or avegra$2 or aybintio$2 or ba1101 or ba-1101 or bambevi$2 or bat1706 or bat-1706 or bcd021 or bcd-021 or bevacizumab$2 or bevacizumabum$2 or bevagen$2 or bevatas$2 or bevax$2 or bevz92 or bevz-92 or bi695502 or bi-695502 or bow030 or bow-030 or boyounuo$2 or bp01 or bp-01 or bp102 or bp-102 or bryxta$2 or bs503a or bs-503a or bxt2316 or bxt-2316 or byvasda$2 or cbt124 or cbt-124 or chs305 or chs-305 or chs5217 or chs-5217 or cizumab$2 or ctp16 or ct-p16 or equidacent$2 or fkb238 or fkb-238 or gb222 or gb-222 or gbs004 or gbs-004 or hanbeitai$2 or hd204 or hd-204 or hlx04 or hlx-04 or hot1010 or hot-1010 or hsdb-8080 or ibi305 or ibi-305 or idb0072 or idb-0072 or intp24 or intp-24 or ipique$2 or jhl1149 or jhl-1149 or js501 or js-501 or jy028 or jy-028 or krabeva$2 or kyomarc$2 or l01xc07 or lextemy$2 or "lumiere-(drug)" or ly01008 or ly-01008 or mabionvegf$2 or mb02 or mb-02 or mil60 or mil-60 or mvasi$2 or myl14020 or myl-14020 or myl1402o or myl-1402o or nsc704865 or nsc-704865 or onbevzi$2 or ons1045 or ons-1045 or ons5010 or ons-5010 or oyavas$2 or pf06439535 or pf-06439535 or pf6439535 or pf-6439535 or pmc901 or pmc-901 or pobevcy$2 or pro169 or pro-169 or pusintin$2 or ql1101 or ql-1101 or r435 or r-435 or rg435 or rg-435 or rhumab$2 or ro4876646 or ro-4876646 or rph001 or rph-001 or rtpr023 or r-tpr-023 or sb8 or sb-8 or sct501 or sct-501 or sct510 or sct-510 or sibp04 or sibp-04 or stc103 or stc-103 or stivant$2 or tab008 or tab-008 or tab014 or tab-014 or tot102 or tot-102 or trs003 or trs-003 or tx16 or tx-16 or vegzelma$2 or versavo$2 or zirabev$2 or zrc113 or zrc-113 or zybev$2).ti,ab,kw,kf,ot,rn,dq. [BEVACIZUMAB TERMS] | 128013 |
| 232 | dostarlimab/ or (2022215-59-2 or anb011 or anb-011 or dostarlimab$2 or gsk4057190 or gsk-4057190 or jemperli$2 or p0gvq9a4s5 or tsr042 or tsr-042 or wbp285 or wbp-285).ti,ab,kw,kf,ot,rn,dq. [DOSTARLIMAB TERMS] | 925 |
| 233 | (chembl5095383 or retlirafusp-alfa or shr1701 or shr-1701).ti,ab,kw,kf,ot,rn,dq. [SHR-1701 TERMS] | 56 |
| 234 | domvanalimab/ or (2368219-35-4 or 45x7ou8c4j or ab154 or ab-154 or domvanalimab$2 or who-11559).ti,ab,kw,kf,ot,rn,dq. [DOMVANALIMAB TERMS] | 101 |
| 235 | zimberelimab/ or (2259860-24-5 or ab122 or ab-122 or gls010 or gls-010 or gs0122 or gs-0122 or wbp3055 or wbp-3055 or who-11413 or zbl7o904il or zimberelimab$2).ti,ab,kw,kf,ot,rn,dq. [ZIMBERELIMAB TERMS] | 225 |
| 236 | lapatinib/ or (0vua21238f or 1092929-10-6 or 1210608-87-9 or 1xkk or 231277-92-2 or 388082-78-8 or 437755-78-7 or 913989-15-8 or a25184 or ab01273965-01 or ab01273965-02 or ab01273965-03 or ab01273965-04 or ab01273965-05 or ac-1314 or akos005145766 or am20090641 or as-14065 or bc164610 or bcp01874 or bcp9000837 or bcp9000838 or bcpp000188 or bcpp000189 or bdbm5445 or brd-k19687926-001-01-7 or brd-k19687926-379-02-5 or c29h26clfn4o4s or ccg-270133 or chebi-49603 or chembl554 or cid-208908 or d08108 or db01259 or dtxcid5026675 or dtxsid7046675 or en300-117254 or ex-a402 or fmm$2 or ft-0659650 or gsk572016 or gsk-572016 or gtpl5692 or gw2016 or gw-2016 or gw282974x or gw-282974x or gw572016 or gw-572016 or gw572016f or gw-572016f or gw-572016x or hms2089h10 or hms3244n06 or hms3244n10 or hms3244n14 or hms3744k11 or hsdb-8209 or hy-50898 or kinome-3684 or kinome-3685 or l0360 or lapatinib$2 or mfcd09264194 or ncgc00167507-01 or ncgc00167507-02 or ncgc00167507-03 or ncgc00167507-04 or ncgc00167507-09 or ns00003012 or nsc745750 or nsc-745750 or nsc800780 or nsc-800780 or q-101353 or q420323 or sb16918 or schembl8100 or sr-05000001472-1 or sw199101-5 or tox21-112505 or tykerb$2 or tyverb$2).ti,ab,kw,kf,ot,rn,dq. [LAPATINIB TERMS] | 21624 |
| 237 | (857890-39-2 or a825653 or ac-25047 or aiv007 or aiv-007 or akos025401742 or amy9240 or as-16203 or bcp01799 or bcp9000633 or bcpp000247 or bdbm50331094 or bl164616 or c21h19cln4o4 or ccg-264842 or chebi-85994 or chembl1289601 or cs-0109 or d09919 or db09078 or dtxcid50117096 or dtxsid50194605 or e7080 or e-7080 or ee083865g2 or en300-7418350 or er203492-00 or er-203492-00 or ex-a249 or ft-0700727 or gtpl7426 or hms3244a07 or hms3244a08 or hms3244b07 or hms3654a14 or hy-10981 or j-513372 or kisplyx$2 or l01xe29 or lenvatinib$2 or lenvatinibum$2 or lenvima$2 or lev$2 or mfcd16038644 or mk7902 or mk-7902 or mls006011239 or ncgc00263198-01 or ncgc00263198-04 or ncgc00263198-07 or ns00069283 or nsc755980 or nsc-755980 or nsc800781 or nsc-800781 or q6523413 or ro7071618 or ro-7071618 or s1164 or sb16580 or schembl864638 or smr004702999 or sw219259-1 or z2235801899).ti,ab,kw,kf,ot,hw,rn,nm. [LENVATINIB TERMS] | 8193324 |
| 238 | lenvatinib/ or (857890-39-2 or a825653 or ac-25047 or aiv007 or aiv-007 or akos025401742 or amy9240 or as-16203 or bcp01799 or bcp9000633 or bcpp000247 or bdbm50331094 or bl164616 or c21h19cln4o4 or ccg-264842 or chebi-85994 or chembl1289601 or cs-0109 or d09919 or db09078 or dtxcid50117096 or dtxsid50194605 or e7080 or e-7080 or ee083865g2 or en300-7418350 or er203492-00 or er-203492-00 or ex-a249 or ft-0700727 or gtpl7426 or hms3244a07 or hms3244a08 or hms3244b07 or hms3654a14 or hy-10981 or j-513372 or kisplyx$2 or l01xe29 or lenvatinib$2 or lenvatinibum$2 or lenvima$2 or lev$2 or mfcd16038644 or mk7902 or mk-7902 or mls006011239 or ncgc00263198-01 or ncgc00263198-04 or ncgc00263198-07 or ns00069283 or nsc755980 or nsc-755980 or nsc800781 or nsc-800781 or q6523413 or ro7071618 or ro-7071618 or s1164 or sb16580 or schembl864638 or smr004702999 or sw219259-1 or z2235801899).ti,ab,kw,kf,ot,rn,dq. [LENVATINIB TERMS] | 6298339 |
| 239 | ipatasertib/ or (0rf or 1001264-89-6 or 524y3ib4hq or ac-28420 or akos025396463 or as-17027 or bcp0726000195 or bcp9000712 or bdbm50398379 or ccg-269312 or chebi-95089 or chembl2177390 or cs-0975 or d10641 or db11743 or dtxsid101025595 or ex-a2077 or gdc0068 or gdc-0068 or gdc0068-di-hcl or gdc-0068-di-hcl or gtpl7887 or hy-15186 or ipatasertib$2 or mfcd22124514 or ncgc00346714-01 or ns00072927 or nsc767898 or nsc-767898 or nsc781451 or nsc-781451 or nsc800986 or nsc-800986 or nsc832484 or nsc-832484 or q27078088 or rg7440 or rg-7440 or rg-7440-di-hcl or s2808 or schembl191659).ti,ab,kw,kf,ot,rn,dq. [IPATASERTIB TERMS] | 1189 |
| 240 | or/186-226 [INTERVENTION & COMPARATORS & CHEMO TERMS] | 3121194 |
| 241 | Randomized controlled trial/ or Controlled clinical study/ or randomization/ or intermethod comparison/ or double blind procedure/ or human experiment/ or (compare or compared or comparison or trial).ti. or ((evaluated or evaluate or evaluating or assessed or assess) and (compare or compared or comparing or comparison)).ab. or (random$ or placebo or (open adj label) or ((double or single or doubly or singly) adj (blind or blinded or blindly)) or parallel group$1 or (crossover or cross over) or ((assign$ or match or matched or allocation) adj5 (alternate or group$1 or intervention$1 or patient$1 or subject$1 or participant$1)) or (assigned or allocated) or (controlled adj7 (study or design or trial)) or (volunteer or volunteers)).ti,ab. | 12173464 |
| 242 | (Cross-sectional study/ not (randomized controlled trial/ or controlled clinical study/ or controlled study/ or randomi?ed controlled.ti,ab. or control group$1.ti,ab.)) or ((((case adj control$) and random$) not randomi?ed controlled) or (nonrandom$ not random$) or "Random field$" or (random cluster adj3 sampl$)).ti,ab. or (Systematic review not (trial or study)).ti. or ((review.ab. and review.pt.) not trial.ti.) or ("we searched".ab. and (review.ti. or review.pt.)) or ("update review" or (databases adj4 searched)).ab. or ((rat or rats or mouse or mice or swine or porcine or murine or sheep or lambs or pigs or piglets or rabbit or rabbits or cat or cats or dog or dogs or cattle or bovine or monkey or monkeys or trout or marmoset$1).ti. and animal experiment/) or (Animal experiment/ not (human experiment/ or human/)) | 6389733 |
| 243 | 241 not 242 [RCTs – Embase sensitive Filter – Cochrane HSSS, 2019] | 11058344 |
| 244 | 185 and 240 and 243 | 12106 |
| 245 | (exp adolescent/ or exp child/ or exp fetus/) not (exp adult/ and (exp adolescent/ or exp child/ or exp fetus/)) [CHILDREN <18 REMOVE] | 4551042 |
| 246 | (exp animal/ or exp animal experimentation/ or exp animal model/ or exp animal experiment/ or nonhuman/ or exp vertebrate/) not (exp human/ or exp human experimentation/ or exp human experiment/) [ANIMAL STUDIES ONLY - REMOVE - EMBASE] | 12571890 |
| 247 | (editorial or note or short survey or tombstone).pt. or (letter.pt. not randomized controlled trial/) [OPINION PIECES REMOVE - Embase] | 5397947 |
| 248 | conference abstract.pt. [CONFERENCE ABSTRACTS] | 5047017 |
| 249 | 244 not (245 or 246 or 247) [CHILD <19, ANIMAL STUDIES and OPINION PUBLICATIONS - REMOVED - Embase] | 11981 |
| 250 | 248 and 249 [CONFERENCE ABSTRACTS ONLY] | 2482 |
| 251 | limit 250 to yr="2022 -Current" | 385 |
| 252 | 249 not 248 [CONFERENCE ABSTRACTS REMOVED] | 9499 |
| 253 | 251 or 252 [LAST 2 YRS OF ABSTRACTS RETAINED - Embase] | 9884 |
| 254 | 253 use oemezd [Embase results] | 4604 |
| 255 | Stomach Neoplasms/ or (Esophageal Neoplasms/ and exp Esophagogastric Junction/) | 132993 |
| 256 | Neoplasm Metastasis/ or Neoplasm Recurrence, Local/ | 503214 |
| 257 | ((((stomach? or gastric$ or cardia or cardiac or antrum? or antral$ or fundus$ or pyloric$ or pylorus$ or ventricul$ or linitis plastica or leather-bottle or ((stomach? or gastric$) and (GC or GEJ))) adj3 (neoplas$ or cancer$ or tumo?r$ or carcinoma$ or malignan$ or oncolog$ or adenocancer$ or adeno-cancer$ or adenoma$ or adenocarcinoma$ or adeno-carcinoma$ or blastoma$ or carcinosarcoma$ or carcino-sarcoma$ or adenoacanthoma$ or adeno-acanthoma$ or epithelioma$ or melanoma$ or mesenchymoma$ or sarcoma$ or thymoma$ or granuloma$ or choriocarcinoma$ or cancerogenes?s or carcinoid$)) or ((stomach? or gastric$) adj3 SCC) or ((esophag$ or oesophag$ or esophagogastric$ or esophago-gastric$ or oesophagogastric$ or oesophago-gastric$ or gastroesophageal$ or gastro-esophageal$ or gastrooesophageal$ or gastro-oesophageal$ or cardio-esophageal$ or cardio-oesophageal$ or cardioesophageal$ or cardiooesophageal$ or EG or GE) adj3 (junction$ or sphincter$) adj3 (neoplas$ or cancer$ or tumo?r$ or carcinoma$ or malignan$ or oncolog$ or adenocancer$ or adeno-cancer$ or adenoma$ or adenocarcinoma$ or adeno-carcinoma$ or blastoma$ or carcinosarcoma$ or carcino-sarcoma$ or adenoacanthoma$ or adeno-acanthoma$ or epithelioma$ or melanoma$ or mesenchymoma$ or sarcoma$ or thymoma$ or granuloma$ or choriocarcinoma$ or cancerogenes?s or carcinoid$))) adj4 ((meta adj sta$) or metastas$ or metastatic$ or recur$ or secondar$ or relaps$ or advance$ or inoperab$ or disseminat$ or spread or migration or lethal$ or incurable or noncurable or non-curable or uncurable or progressive or terminal or invasive$ or aggressive$ or (late? adj2 stage$) or ((stage? or grade? or type?) adj2 (3a$ or 3b$ or 3c$ or III$ or 4a$ or 4b$ or IV$)) or "stage 3" or "stage 4" or met or mets or N1? or N2? or N3? or pN1? or pN2? or pN3?)).ti,ab,kw. [Metastatic GC/GEJ TERMS] | 71583 |
| 258 | (255 and 256) or 257 [GC-GEJ TERMS] | 76784 |
| 259 | (tislelizumab$2 or tirelizumab$2 or bgb-a317 or bgba317 or bgn-1 or bgn1 or jhl-2108 or jhl2108 or vdt-482 or vdt482 or 1858168-59-8 or 0kvo411b3n).ti,ab,kw. [TISLELIZUMAB TERMS] | 1319 |
| 260 | Immune Checkpoint Inhibitors/ or ((Programmed Cell Death 1 Receptor/ or Programmed Cell Death 1 Ligand 2 Protein/) and (inhibit$ or block?).ti,ab,kw,kf.) or ((immune$ adj3 checkpoint? adj3 (inhibit$ or block?)) or (((programmed adj3 death) or PD-1 or PD-1-PD-L1 or PDCD1) adj3 (ligand? or inhibit$ or block?)) or ((B7-H1 or B7H1 or "B7 homolog 1" or CD274 or CD273 or PDCD1LG1 or PDCD1LG2) adj3 (antigen? or protein?)) or ((Cytotoxic-T-Lymphocyte-Associated Protein-4 Inhibitor? or CTLA-4) adj3 (inhibit$ or block?)) or (ICI? and "Immun$ Checkpoint") or BMS-1 or EX-A947 or HY-19991 or J-690233 or MFCD28978741 or s7911 or D000082082 or SCHEMBL16555159 or ZINC230477930 or 1675201-83-8).ti,ab,kw. [IMMUNE CHECKPOINT PROTEINS TERMS] | 136277 |
| 261 | Immunotherapy/ or Radioimmunotherapy/ or Antibodies, Monoclonal/ or (immunotherap$ or immuno-therap$ or (((biologic$ adj3 response? adj3 modifier?) or BRM or immunogenic$ or immunologic$ or immuno-genic$ or immuno-logic$ or radioimmunotherapy$ or radio-immunotherap$ or ((monoclonal$ or clonal$ or hybridoma$) adj2 antibod$)) adj3 (therap$ or intervention? or treat$))).ti,ab,kw. [IMMUNOTHERAPY TERMS] | 894311 |
| 262 | Molecular Targeted Therapy/ or ((molecular$ or neoplas$ or cancer$ or tumo?r$ or carcinoma$) adj3 target$ adj3 therap$).ti,ab,kw. [TARGETED THERAPY TERMS] | 187317 |
| 263 | (atezolizumab$2 or anti-PDL1 or MPDL-3280A or MPDL3280A or RG-7446 or RG7446 or ro-5541267 or ro5541267 or Tecentriq$2 or Tecntriq$2 or 1380723-44-3 or 0INE2SFD9E or 52CMI0WC3Y).ti,ab,kw. [ATEZOLIZUMAB TERMS] | 12948 |
| 264 | (avelumab$2 or bavencio$2 or msb-0010682 or msb-0010718c or msb0010682 or msb0010718c or msb-10682 or msb-10718c or msb10682 or msb10718c or pf-06834635 or pf-6834635 or pf06834635 or pf6834635 or KXG2PJ551I or 1537032-82-8).ti,ab,kw. [AVELUMAB TERMS] | 3518 |
| 265 | (camrelizumab$2 or "anti-pd-1 monoclonal antibody" or shr-1210 or shr1210 or carilizumab$2 or carrelizumab$2 or 73096E137E or 1798286-48-2).ti,ab,kw. [CAMRELIZUMAB TERMS] | 2922 |
| 266 | (1428935-60-7 or 28x28x9okv or anti-b7h1-monoclonal-antibody or durvalumab$2 or durvalumabum$2 or imfinzi$2 or l01xc28 or medi4736 or medi-4736).ti,ab,kw. [DURVALUMAB TERMS] | 7170 |
| 267 | Ipilimumab/ or (ipilimumab$2 or bms-734016 or bms734016 or cs-1002 or cs1002 or ibi-310 or ibi310 or mdx-ctla-4 or mdx-010 or mdx-101 or mdx010 or mdx101 or strentarga$2 or yervoy$2 or 6T8C155666 or 477202-00-9).ti,ab,kw. [IPILIMUMAB TERMS] | 34671 |
| 268 | Nivolumab/ or (nivolumab$2 or bms-936558 or bms-986213 or bms-986298 or cmab819 or bms936558 or bms986213 or bms986298 or cmab-819 or mdx-1106 or mdx1106 or ono-4538 or ono4538 or opdivo$2 or opdualag$2 or 31YO63LBSN or 946414-94-4).ti,ab,kw. [NIVOLUMAB TERMS] | 55238 |
| 269 | (pembrolizumab$2 or keytruda$2 or lambrolizumab$2 or mk3475 or mk-1308a or mk-3475 or mk7684a or sch-900475 or sch900475 or "keylynk-010 component" or DPT0O3T46P or 1422183-02-5 or 1374853-91-4).ti,ab,kw. [PEMBROLIZUMAB TERMS] | 32744 |
| 270 | (2072873-06-2 or 8fu7fq8upk or ibi308 or ibi-308 or sintilimab$2 or tyvyt$2 or who-10801).ti,ab,kw. [SINTILIMAB TERMS] | 1605 |
| 271 | (1924598-82-2 or 8jxn261vva or js001 or js-001 or tab001 or tab-001 or teripalimab$2 or toripalimab$2 or treipril$2 or treprizumab$2 or tripleitriumab$2 or triprizumab$2 or tuoyi$2 or who-10820).ti,ab,kw. [TORIPALIMAB TERMS] | 1078 |
| 272 | (2079108-44-2 or 2226345-85-1 or 2y3t5if01z or aex1188 or aex-1188 or incmga00012 or incmga-00012 or incmga0012 or incmga-0012 or mga012 or mga-012 or retifanlimab$2 or zynyz$2).ti,ab,kw. [RETIFANLIMAB TERMS] | 120 |
| 273 | (2245725-04-4 or l62556gpxb or mgd013 or mgd-013 or tebotelimab$2).ti,ab,kw. [TEBOTELIMAB TERMS] | 36 |
| 274 | (2394841-59-7 or 6fyg1ds4nw or ak104 or ak-104 or cadonilimab$2 or who-11581).ti,ab,kw. [CADONILIMAB TERMS] | 86 |
| 275 | (2231029-82-4 or hlx10 or hlx-10 or s3gqz2k36v or serplulimab$2).ti,ab,kw. [SERPLULIMAB TERMS] | 132 |
| 276 | (2256084-03-2 or 90iqr2i6tr or cs1001 or cs-1001 or sugemalimab$2 or wbp315 or wbp-315 or wbp3155 or wbp-3155).ti,ab,kw. [SUGEMALIMAB TERMS] | 127 |
| 277 | (1496553-00-4 or claudiximab$2 or imab362 or imab-362 or tf5mpq8wgy or zolbetuximab$2).ti,ab,kw. [ZOLBETUXIMAB TERMS] | 254 |
| 278 | (1952272-74-0 or bemarituzumab$2 or fpa144 or fpa-144 or rjw23bq0kw).ti,ab,kw. [BEMARITUZUMAB TERMS] | 84 |
| 279 | Cetuximab/ or (205923-56-4 or abp494 or abp-494 or c225 or c-225 or c225-03 or c-22503 or c-225-03 or cdp1 or cdp-1 or cetuximab$2 or cetuximabum$2 or ch225 or ch-225 or chimeric-anti-egfr-monoclonal-antibody or cmab009 or cmab-009 or ctp15 or ct-p15 or dtxsid0040830 or erbitux$2 or hsdb-7454 or imc225 or imc-225 or imcc225 or imcc-225 or imc-c225 or kl140 or kl-140 or l01xc06 or ly2939777 or ly-2939777 or mab-c225 or moab-c225 or nsc714692 or pqx0d8j21j or sti001 or sti-001).ti,ab,kw. [CETUXIMAB TERMS] | 48474 |
| 280 | (1133766-06-9 or metmab$2 or metma-b or ms1j9720wc or onartuzumab$2 or pro143966 or pro-143966 or pro-143996 or pro143996 or ro5490258 or ro-5490258).ti,ab,kw. [ONARTUZUMAB TERMS] | 347 |
| 281 | (51wew898ij or 872514-65-3 or amg102 or amg-102 or rilotumumab$2).ti,ab,kw. [RILOTUMUMAB TERMS] | 259 |
| 282 | (1518996-49-0 or 571045eim4 or andecaliximab$2 or gs5745 or gs-5745).ti,ab,kw. [ANDECALIXIMAB TERMS] | 135 |
| 283 | (444731-52-6 or 635702-64-6 or 790713-33-6 or 7rn5dr86ck or a19406 or a839572 or ab01273967-01 or ab01273967-02 or ab01273967-05 or ab01273967-06 or ac-8522 or akos005145819 or am20090659 or ar-270-43507999 or armala$2 or as-11066 or bcp01839 or bcp9001053 or bcpp000129 or bd164238 or bdbm26474 or brd-k74514084-003-02-7 or ccg-265010 or chebi-71219 or chembl477772 or cid-10113978 or cs-0269 or db06589 or dtxcid1028659 or dtxsid8048733 or en300-57325 or ex-a1241 or ft-0659928 or ft-0684794 or gtpl5698 or gw780604 or gw-780604 or gw-78603 or gw786034 or gw7-86034 or gw-786034 or gw786034b or gw-786034b or gw786034x or gw-786034x or hms3244c21 or hms3244c22 or hms3244d21 or hms3656l14 or hms3745g05 or hsdb-8210 or hy-10208 or indazolylpyrimidine-13 or jmc514632-compound-13 or kinome-3790 or mfcd11616589 or ncgc00188865-01 or ncgc00188865-02 or ncgc00188865-03 or ncgc00188865-10 or nsc752782 or nsc-752782 or nsc800839 or nsc-800839 or p-6706 or pazopanib$2 or pazopanibum$2 or q-101400 or q7157043 or s3012 or sb17290 or sb710468 or sb-710468 or sb710468a or sb-710468a or schembl588608 or sw218082-3 or tox21-113174 or tox21-113174-1 or votrient$2 or z1541638525).ti,ab,kw. [PAZOPANIB TERMS] | 7544 |
| 284 | (1218779-75-9 or 5s371k6132 or 811803-05-1 or ab01274807-01 or ab01274807-02 or ac-27461 or akos024464453 or amy21302 or apatinib$2 or ba175030 or bcp02840 or c76598 or ccg-268625 or chembl3186534 or cs-0003200 or d11288 or db14765 or ds-7455 or dtxsid601024366 or ex-a1794 or gtpl7648 or hy-13342a or mfcd21648511 or ncgc00249393-01 or ncgc00249393-08 or nsc772886 or nsc-772886 or nsc799333 or nsc-799333 or q27262801 or rivoceranib$2 or s5248 or sb16590 or schembl1814966 or yn968d1 or yn-968d1).ti,ab,kw. [RIVOCERANIB/APATINIB TERMS] | 3781 |
| 285 | Induction Chemotherapy/ or Consolidation Chemotherapy/ or Maintenance Chemotherapy/ or Antineoplastic Combined Chemotherapy Protocols/ or exp Chemotherapy, Adjuvant/ or Chemoradiotherapy/ or (chemotherap$ or chemo-therap$ or carcinochemotherap$ or chemoradiotherap$ or chemoradiation? or radiochemotherap$ or carcino-chemotherap$ or chemo-radiotherap$ or chemo-radiation? or radio-chemotherap$).ti,ab,kw. [CHEMOTHERAPY TERMS] | 1837236 |
| 286 | exp Leucovorin/ or (leucovorin$ or 6-s-leucovorin or 6s-leucovorin or acide folinique or akos015961207 or bdbm50039121 or bpbio1-000766 or bspbio-000696 or bspbio-002218 or brd-a75919782-238-01-8 or calcium folinate or chebi-15640 or chembl1679 or chembl69905 or citrovoeum-factor or citrovorum-factor or d93089 or divk1c-000222 or dtxsid0048216 or einecs-200-361-6 or en300-27068710 or folinate folinic-acid-sf or folinic acid or formyltetrahydrofolate or fusilev$2 or gtpl4816 or gtpl6690 or hsdb-6544 or hy-17556 or idi1-000222 or kbio1-000222 or kbio2-001339 or kbio2-003907 or kbio2-006475 or kbio3-001438 or kbiogr-000461 or kbioss-001339 or lencovorin$2 or leucal$2 or leukovorin$2 or leukovoran$2 or leucovorin$2 or levoleucovorin$2 or levo-leucovorin$2 or mfcd00867488 or ninds-000222 or nsc3590 or prestwick0-000738 or prestwick1-000738 or prestwick2-000738 or prestwick3-000738 or q45435667 or q573i9dvlp or s5790 or schembl10068238 or schembl8349 or sd-204098 or s-leucovorin$2 or sleucovorin$2 or spectrum2-000116 or spectrum3-000479 or spectrum4-000031 or spectrum5-000910 or spectrum-000859 or spbio-000132 or spbio-002635 or sbi-0051427-p003 or welcovorin$2 or "formyltetrahydropteroylglutamic acid" or Q573I9DVLP or 58-05-9).ti,ab,kw. [LEUCOVORIN TERMS] | 67520 |
| 287 | Carboplatin/ or (Carboplatin$2 or blastocarb$2 or boplatex$2 or carboplat$ or carbosin$2 or carbotec$2 or carplan$2 or CBDCA or (platinum adj3 (cis or diamin? or cyclobutanedicarboxylat? or dicarboxylatediammine)) or Dicarboxylatoplatinum or diamminecyclobutanedicarboxylatoplatinum or cycloplatin$ or erbakar$2 or ercar$2 or ifacap$2 or jm8 or jm-8 or kemocarb$2 or nsc-241240 or nsc241240 or nsc-201345 or nsc201345 or oncocarbin$2 or paraplatin$ or Platinwas$2 or Ribocarbo$2 or Neocarbo$2 or Nealorin$2 or HSDB-6957 or BG3F62OND5 or 41575-94-4).ti,ab,kw. [CARBOPLATIN TERMS] | 128900 |
| 288 | exp Paclitaxel/ or (paclitaxel$ or abraxane$2 or abraxus$2 or act02709 or act-02709 or acon1-002231 or anx-513 or anzatax$2 or apealea$2 or asotax$2 or bidd-pxr0046 or biotax$2 or bms-181339 or bms181339-01 or bms181339 or bms-181339-01 or bmy-45622 or bmy45622 or bspbio-000290 or capxol$2 or ccris-8143 or chembl428647 or chebi-45863 or coroflex-please$2 or coroxane$2 or cmap-000068 or cynviloq$2 or cypher-select$2 or dsstox-cid-3413 or dsstox-gsid-23413 or dsstox-rid-77016 or dhp107 or dhp-107 or dhp-208 or dhp208 or dts-301 or dts301 or ebetaxel$2 or empac$2 or endotag-1 or endotag1 or formoxol$2 or genaxol$2 or genetaxyl$2 or genexol$2 or gtpl2770 or hms2090d07 or hms2095o12 or hms2231a16 or hms3712o12 or hsdb-6839 or hunxol$2 or hy-b0015 or ifaxol$2 or ig-001 or ig001 or infinnium$2 or intaxel$2 or kbiogr-002509 or kbio2-002509 or kbio2-005077 or kbio2-007645 or kbio3-002987 or lep-etu$2 or lipopac$2 or liporaxel$2 or mbt-0206 or mbt0206 or medixel or mfcd00869953 or mitotax$2 or nanopac$2 or nanotax$2 or nanotaxel$2 or ncgc00164367-01 or nk-105 or nk105 or nsc-125973 or nsc-673089 or nsc125973 or nsc673089 or nscc-125973 or nova-12005 or oas-pac-100 or oaspac100 or oncogel$2 or onxal$2 or onxol$2 or p-ssmm-vip$2 or paclical$2 or pacitaxel$2 or paclical$2 or padexol$2 or pacligel$2 or paclitaxel$2 or pacliex$2 or paxceed$2 or paxene$2 or paxoral$2 or paxus$2 or pazenir$2 or plaxicel$2 or praxel$2 or qw-8184 or schembl3976 or sb-05 or sb05 or sdp-013 or sindaxel$2 or smr000857385 or sr-01000075350 or taycovit$2 or taxalbin$2 or taxane$ or taxocris$2 or taxol$2 or taxus$2 or tocosol$2 or xorane$2 or yewtaxan$2 or zinc96006020 or zisu$2 or P88XT4IS4D or 33069-62-4).ti,ab,kw. [PACLITAXEL TERMS] | 230272 |
| 289 | Docetaxel/ or (114915-20-7 or 114977-28-5 or 15h5577cqd or 699121phca or ab01273941-01 or ab01273941-02 or ac-383 or akos015960718 or akos024457953 or amy4356 or anx-514 or axtere$2 or bd164373 or bdbm36351 or bind014 or bind-014 or brd-k30577245-001-04-3 or brd-k30577245-341-01-9 or bs102 or bs-102 or chebi-4672 or chembl92 or cid148124 or ckd-810 or crlx301 or crlx-301 or cs-1144 or d07866 or d4102 or daxotel$2 or db01248 or dexotel$2 or docecad$2 or docefrez$2 or docetaxel$2 or docetaxelum$2 or docetaxol$2 or docetaxolum$2 or dtxcid8020464 or dtxsid0040464 or emdoc$2 or en300-123047 or ex-a1206 or gtpl6809 or hms2089k08 or hsdb-6965 or hy-b0011 or ks-1452 or l01cd02 or lit976 or lit-976 or mfcd00871399 or ncgc00181306-01 or ncgc00181306-02 or ncgc00181306-04 or ncgc00242509-01 or nsc628503 or nsc-628503 or nsc-759850 or oncodocel$2 or q-100074 or q420436 or rp56976 or rp-56976 or schembl4419 or sdp-014 or sid-530 or sr-01000003023 or sr-01000003023-5 or syp-0704a or taxanit$2 or taxespira$2 or taxoel$2 or taxoltere-metro or taxotel$2 or taxoter$2 or taxotere$2 or texot$2 or tox21-112781 or tox21-113088 or txl$2 or w-60384 or xrp6976 or xrp-6976 or xrp-6976l or z1546621742).ti,ab,kw. [DOCETAXEL TERMS] | 107602 |
| 290 | Cisplatin/ or (Cisplatin$ or platinum$ or Cismaplat$2 or (cis adj3 ($platinum or platinous)) or cis-platinum or cis-Platin or dichloroplatinum or diaminodichloroplatinum or diamminedichloroplatinum or dichlorodiammineplatinum or AI3-62048 or abiplatin or biocisplatinum or biocysplatinum or blastolem$2 or briplatin$2 or cddp-ti or cis-ddp or cisPt$ or CACP or CCRIS-221 or CDDP or DDPt or CP-Ethypharm or CPDC or CPDD or CPPD or (DDP and antitumor) or cisplatyl$2 or citoplatino$2 or cytoplatin$2 or cytosplat$2 or docistin$2 or elvecis$2 or kemoplat$2 or Fauldiscipla$2 or IA-call or LiPlaCis$2 or lederplatin$2 or lipoplatin$2 or "liposomal cisplatin" or mpi-5010 or mpi5010 or neoplatin$2 or niyaplat$2 or nk-801 or noveldexis$2 or nsc-119875 or nci-c55776 or platamine$2 or platamine-rtu or platiblastin$2 or platidiam$2 or platimine$2 or platinex$2 or platinil$2 or platino$2 or platinol$2 or platinolaq$2 or platinol-aq$2 or platinoxan$2 or platiran$2 or platistil$2 or platistin$2 or platosin$2 or "pronto platamine" or "Peyrone's chloride" or randa$2 or romcis$2 or sicatem$2 or spi-077 or tr-170 or tecnoplatin$2 or Q20Q21Q62J or 15663-27-1 or 26035-31-4 or 96081-74-2).ti,ab,kw. [CISPLATIN TERMS] | 448473 |
| 291 | Oxaliplatin/ or (oxaliplatin$2 or (Oxalat$ adj3 platin$) or axiplatin$2 or bendaplatin$2 or crisapla$2 or croloxat$2 or dacotin$2 or dacplat$2 or ebeoxal$2 or elatofen$2 or eloxatin$ or elplat$2 or euroxaliplatin$2 or geneplatin$2 or gessedil$2 or heloxatin$2 or lipoxal$2 or mbp-426 or mbp426 or medoxa$2 or oksaliplatin$ or oplat$2 or oxalatoplatin$ or oxalatplatin$2 or oxali$2 or oxalip$2 or oxaliplan$2 or oxaliprol$2 or oxaliquid$2 or oxalisan$2 or oxalisin$2 or oxalizor$2 or oxaltic$2 or oxaltin$2 or oxamed$2 or oxaplamyl$2 or oxaviatin$2 or platox$2 or plaxitin$2 or rectoxal$2 or riboxatin$2 or rp-54780 or rp54780 or sinoxal$2 or sr-96669 or sr96669 or transplastin$2 or velminox$2 or xaliplat$2 or xoplan$2 or L-OHP-Cpd or 1-OHP or ACT-078 or ACT078 or CCRIS-9143 or NSC-266046 or 04ZR38536J or 61825-94-3).ti,ab,kw. [OXALIPLATIN TERMS] | 90494 |
| 292 | Capecitabine/ or (capecitabin$ or apecitab$2 or atubri$2 or bc164277 or bcpp000300 or bxeliri$2 or bs-1000 or cacit$2 or capcel$2 or capebina$2 or capecite$2 or capegard$2 or capezam$2 or capicet$2 or capiri$2 or capiibine$2 or captabin$2 or capnat$2 or capoda$2 or capostat$2 or capsy$2 or capxcel$2 or caxeta$2 or ccg-264841 or ccx-340 or cpecitabine$2 or cs-0768 or d01223 or db01101 or dsstox-cid-26451 or dsstox-gsid-46451 or dsstox-rid-81625 or dtxsid3046451 or ecansya$2 or ex-a835 or gtpl6799 or hsdb-7656 or hy-b0016 or j-700154 or k007 or m0297 or mfcd00930626 or mls003915642 or mls004774137 or ncgc00164569-01 or ncgc00164569-02 or ncgc00164569-05 or nsc-759853 or paxon$2 or q-200788 or q420207 or r-340 or rg-340 or r340 or rg340 or ro-09-1978 or ro-091978 or ro-09-1978 or ro-09-1978-000 or ro091978 or s1156 or s-1156 or sr-01000931255 or tox21-112198 or x-abine$2 or x-tabin$2 or xabine$2 or xecap$2 or xeliri$2 or xelocel$2 or xeloda$2 or xelox$2 or z1501480421 or zinc3806413 or 6804dj8z9u or 154361-50-9 or 958887-39-3).ti,ab,kw. [CAPECITABINE TERMS] | 56468 |
| 293 | (platinum adj1 (fluoropyrimidine or fluoro-pyrimidine) adj3 (doublet? or combin$ or chemotherap$ or chemo-therap$ or ((first or front) adj1 line?) or 1-LOT or 1L or therap$ or regimen? or expos$)).ti,ab,kw. [PLATINUM-FLUOROPYRIMIDINE DOUBLET TERMS] | 253 |
| 294 | (5-fluoropyrimidin$ or 5-fluoro-pyrimidine or pyrimidine-5-fluoro or (fluorinated adj1 pyrimidine) or a9048 or ac-453 or akos006346044 or am86123 or "bb 0260992" or c4h3fn2 or db-007051 or dtxsid80217851 or en300-6966105 or f14737 or ft-0601423 or mfcd06658278 or q42859845 or w-203496 or zinc1845840 or 675f218 or L36X4TD47C or 675-21-8).ti,ab,kw,kf,ot,hw,rn,nm. [FLUOROPYRIMIDINE TERMS] | 5169 |
| 295 | Fluorouracil/ or (fluorouracil$ or fluroblastin$ or 1upf or 5-Faracil or 5-Fluoracil or 5-Fluoracyl or 5-fluoro-uracil or 5-fluoro-uracil or 5-Fluoroblastin or 5-fluorouacil or 5-Ftouracyl or 5-FU or 5FU or 5F-uracil or Adrucil$2 or AI3-25297 or Arumel$2 or BSPBio-002048 or Cancersil$2 or Carac$2 or Carzonal$2 or CHEBI-46345 or CHEMBL185 or Cinco-FU or CCRIS-2582 or DSSTox-CID-634 or DSSTox-GSID-20634 or DSSTox-RID-75705 or Efudex$2 or Efudix$2 or Efurix$2 or EINECS-200-085-6 or Effluderm$2 or Fluoroblastin$2 or Fluoro-Uracil$2 or Fluoro-uracile$2 or Fluoro-uracilo$2 or Fluoroplex$2 or Fluorouracile$2 or Fluorouracilo$2 or Fluorouracilum$2 or Fluorouracilum$2 or Fluracil$2 or Fluracilum$2 or Fluri$2 or Fluril$2 or Fluuro-Uracil$2 or Fluorouracilo$2 or Fluroblastin$2 or Fluro-Uracil$2 or Ftoruracil$2 or GTPL4789 or HSDB 3228 or IDI1-000054 or Kecimeton$2 or KBio1-000054 or KBio2-001321 or KBio2-003889 or KBio2-006457 or KBioGR-001253 or KBioSS-001321 or Lopac-F-6627 or Lopac0-000536 or MFCD00006018 or MLS000069498 or MLS002415705 or NCGC00015442-01 or NCGC00015442-02 or NCGC00015442-03 or NCGC00015442-04 or NCGC00015442-05 or NCGC00015442-06 or NCGC00015442-07 or NCGC00015442-08 or NCGC00015442-09 or NCGC00015442-10 or NCGC00015442-11 or NCGC00015442-12 or NCGC00015442-15 or NCGC00015442-16 or NSC-19893 or NSC757036 or NSC816997 or Phtoruracil$2 or Pharmakon1600-01500305 or Phthoruracil$2 or Queroplex$2 or Ro-2-9757 or S1209 or 191047-64-0 or 191047-65-1 or 191115-88-5 or U3P01618RT or 51-21-8).ti,ab,kw. [FLUOROURACIL TERMS] | 249537 |
| 296 | Irinotecan/ or (irinotecan$ or ab00698464-07 or ab00698464-09 or ab00698464-10 or ab00698464-11 or ab00698464-12 or ab00698464-13 or ab00698464-14 or ac-7469 or akos015894969 or amy4227 or as-14323 or bdbm50128267 or bcp02860 or bcp9000793 or biotecan$2 or brd-k08547377-003-02-4 or campto$2 or camptosar$2 or chebi-80630 or chembl481 or cs-1138 or cpt-11 or cpt11 or d08086 or db00762 or dq2805 or en300-708800 or gtpl6823 or hsdb-7607 or ihl-305 or ihl305 or irinophore-c$2 or irinotel$2 or mfcd00866307 or ncgc00178697-02 or ncgc00178697-05 or nsc-728073 or nsc728073 or nk012-compound or q412197 or s1198 or schembl4034 or sn38 or sn-38 or sn-38-11 or sn3811 or topotecin$2 or u-101440e or u101440e or zinc1612996 or "7673326042" or 100286-90-6 or 97682-44-5).ti,ab,kw. [IRINOTECAN TERMS] | 68609 |
| 297 | (teysuno$2 or (tegafur adj4 gimeracil adj4 oteracil) or ((S-1 or S1) adj3 combination) or TS-1-cpd or S-1-cpd or TS-1 or TS1 or BMS247616 or BMS-247616 or S1-tegafur-oxonate or S1-fluoropyrimidine-oxoonate).ti,ab,kw. [S1 COMBINATION TERMS] | 7916 |
| 298 | Tegafur/ or (1189456-27-6 or 1548r74nsz or 17902-23-7 or 82294-77-7 or a812417 or ab00572620-15 or ac-2112 or akos000121279 or as-13528 or atillon$2 or bcp22714 or bp-58663 or brn-0525766 or c8h9fn2o3 or ccg-100959 or ccg-50110 or ccris-2762 or chebi-32188 or chembl20883 or citofur$2 or coparogin$2 or cs-1128 or d01244 or db09256 or dtxsid001009966 or einecs-241-846-2 or en300-21668 or exonal$2 or f-5-fu or fental$2 or florafur$2 or fluorafur$2 or fluorofur$2 or franrose$2 or franroze$2 or ft-0653732 or ft-0654170 or ft-0674829 or ft-0693965 or ft207 or ft-207 or ftorafur$2 or fulaid$2 or fulfeel$2 or furafluor$2 or furflucil$2 or furofutran$2 or futraful$2 or gtpl10513 or hms1665i05 or hms2051b15 or hms2090k04 or hms2232e05 or hms3371h21 or hms3393b15 or hms3654p13 or hms3715d14 or hy-17400 or lamar$2 or lifril$2 or mfcd00012351 or mjf12264 or mjf-12264 or mls000069497 or mls000759414 or mls001076521 or mls001424119 or nc00209 or ncgc00159418-02 or ncgc00159418-04 or ncgc00159418-05 or neberk$2 or nitobanil$2 or nsc148958 or nsc-148958 or opera-id-1726 or phthorafur$2 or q-201784 or q413370 or racemic-ftorafur or riol$2 or schembl4552 or sfsp$2 or sf-sp or sinoflurol$2 or smr000059106 or sr-01000639511 or sr-01000639511-1 or sr-01000639511-4 or sunfral$2 or sunfural$2 or tefsiel-c or tegaful$2 or tegafur$2 or tegafurum$2 or ts-1 or uftoral$2 or upcmld-dp063 or utefos$2 or z104508106).ti,ab,kw. [TEGAFUR TERMS] | 21276 |
| 299 | Ramucirumab/ or (1121b or 947687-13-0 or 947687-13-0 or a168 or a-168 or cyramza$2 or d99yvk4l0x or hlx12 or hlx-12 or hsdb-8314 or imc1121b or imc1121-b or imc-1121b or imc-1121-b or l01xc21 or ly3009806 or ly-3009806 or nsc-749128 or pbp2001 or pbp-2001 or ramucirumab$2 or ramucirumabum$2 or ro7234952 or ro-7234952).ti,ab,kw. [RAMUCIRUMAB TERMS] | 8883 |
| 300 | Panitumumab/ or (339177-26-3 or 6a901e312a or abenix$2 or abx-egf or amg954 or amg-954 or e7-6-3 or l01xc08 or moab-abx-egf or moab-e7-6-3 or monoclonal-antibody-abx-egf or monoclonal-antibody-e7-6-3 or nsc-742319 or panitumab$2 or panitumumab$2 or panitumumabum$2 or panitunumab$2 or vectibex$2 or vectibix$2).ti,ab,kw. [PANITUMUMAB TERMS] | 13461 |
| 301 | (6ns400bxkh or 780758-10-3 or 828933-51-3 or biomab-egfr or diacim$2 or h-r3 or nimotuzumab$2 or osag-101 or radiotheracim$2 or theracim$2 or theraloc$2).ti,ab,kw. [NIMOTUZUMAB TERMS] | 1470 |
| 302 | (339186-68-4 or emd7200 or emd-7200 or emd72000 or emd-72000 or kgaa$2 or matuzumab$2 or merck-kgaa or mg4m3qb242).ti,ab,kw. [MATUZUMAB TERMS] | 50610 |
| 303 | Bevacizumab/ or (12-igg1 or 1438851-35-4 or 216974-75-3 or 2s9zzm9q9v or abevmy$2 or abp215 or abp-215 or ainex$2 or altuzan$2 or alymsys$2 or ankeda$2 or anti-vegf or askb1202 or ask-b1202 or avastin$2 or avegra$2 or aybintio$2 or ba1101 or ba-1101 or bambevi$2 or bat1706 or bat-1706 or bcd021 or bcd-021 or bevacizumab$2 or bevacizumabum$2 or bevagen$2 or bevatas$2 or bevax$2 or bevz92 or bevz-92 or bi695502 or bi-695502 or bow030 or bow-030 or boyounuo$2 or bp01 or bp-01 or bp102 or bp-102 or bryxta$2 or bs503a or bs-503a or bxt2316 or bxt-2316 or byvasda$2 or cbt124 or cbt-124 or chs305 or chs-305 or chs5217 or chs-5217 or cizumab$2 or ctp16 or ct-p16 or equidacent$2 or fkb238 or fkb-238 or gb222 or gb-222 or gbs004 or gbs-004 or hanbeitai$2 or hd204 or hd-204 or hlx04 or hlx-04 or hot1010 or hot-1010 or hsdb-8080 or ibi305 or ibi-305 or idb0072 or idb-0072 or intp24 or intp-24 or ipique$2 or jhl1149 or jhl-1149 or js501 or js-501 or jy028 or jy-028 or krabeva$2 or kyomarc$2 or l01xc07 or lextemy$2 or "lumiere-(drug)" or ly01008 or ly-01008 or mabionvegf$2 or mb02 or mb-02 or mil60 or mil-60 or mvasi$2 or myl14020 or myl-14020 or myl1402o or myl-1402o or nsc704865 or nsc-704865 or onbevzi$2 or ons1045 or ons-1045 or ons5010 or ons-5010 or oyavas$2 or pf06439535 or pf-06439535 or pf6439535 or pf-6439535 or pmc901 or pmc-901 or pobevcy$2 or pro169 or pro-169 or pusintin$2 or ql1101 or ql-1101 or r435 or r-435 or rg435 or rg-435 or rhumab$2 or ro4876646 or ro-4876646 or rph001 or rph-001 or rtpr023 or r-tpr-023 or sb8 or sb-8 or sct501 or sct-501 or sct510 or sct-510 or sibp04 or sibp-04 or stc103 or stc-103 or stivant$2 or tab008 or tab-008 or tab014 or tab-014 or tot102 or tot-102 or trs003 or trs-003 or tx16 or tx-16 or vegzelma$2 or versavo$2 or zirabev$2 or zrc113 or zrc-113 or zybev$2).ti,ab,kw. [BEVACIZUMAB TERMS] | 127493 |
| 304 | (2022215-59-2 or anb011 or anb-011 or dostarlimab$2 or gsk4057190 or gsk-4057190 or jemperli$2 or p0gvq9a4s5 or tsr042 or tsr-042 or wbp285 or wbp-285).ti,ab,kw. [DOSTARLIMAB TERMS] | 493 |
| 305 | (chembl5095383 or retlirafusp-alfa or shr1701 or shr-1701).ti,ab,kw,kf,ot,hw,rn,nm. [SHR-1701 TERMS] | 56 |
| 306 | (2368219-35-4 or 45x7ou8c4j or ab154 or ab-154 or domvanalimab$2 or who-11559).ti,ab,kw. [DOMVANALIMAB TERMS] | 49 |
| 307 | (2259860-24-5 or ab122 or ab-122 or gls010 or gls-010 or gs0122 or gs-0122 or wbp3055 or wbp-3055 or who-11413 or zbl7o904il or zimberelimab$2).ti,ab,kw. [ZIMBERELIMAB TERMS] | 126 |
| 308 | Lapatinib/ or (0vua21238f or 1092929-10-6 or 1210608-87-9 or 1xkk or 231277-92-2 or 388082-78-8 or 437755-78-7 or 913989-15-8 or a25184 or ab01273965-01 or ab01273965-02 or ab01273965-03 or ab01273965-04 or ab01273965-05 or ac-1314 or akos005145766 or am20090641 or as-14065 or bc164610 or bcp01874 or bcp9000837 or bcp9000838 or bcpp000188 or bcpp000189 or bdbm5445 or brd-k19687926-001-01-7 or brd-k19687926-379-02-5 or c29h26clfn4o4s or ccg-270133 or chebi-49603 or chembl554 or cid-208908 or d08108 or db01259 or dtxcid5026675 or dtxsid7046675 or en300-117254 or ex-a402 or fmm$2 or ft-0659650 or gsk572016 or gsk-572016 or gtpl5692 or gw2016 or gw-2016 or gw282974x or gw-282974x or gw572016 or gw-572016 or gw572016f or gw-572016f or gw-572016x or hms2089h10 or hms3244n06 or hms3244n10 or hms3244n14 or hms3744k11 or hsdb-8209 or hy-50898 or kinome-3684 or kinome-3685 or l0360 or lapatinib$2 or mfcd09264194 or ncgc00167507-01 or ncgc00167507-02 or ncgc00167507-03 or ncgc00167507-04 or ncgc00167507-09 or ns00003012 or nsc745750 or nsc-745750 or nsc800780 or nsc-800780 or q-101353 or q420323 or sb16918 or schembl8100 or sr-05000001472-1 or sw199101-5 or tox21-112505 or tykerb$2 or tyverb$2).ti,ab,kw. [LAPATINIB TERMS] | 21569 |
| 309 | (857890-39-2 or a825653 or ac-25047 or aiv007 or aiv-007 or akos025401742 or amy9240 or as-16203 or bcp01799 or bcp9000633 or bcpp000247 or bdbm50331094 or bl164616 or c21h19cln4o4 or ccg-264842 or chebi-85994 or chembl1289601 or cs-0109 or d09919 or db09078 or dtxcid50117096 or dtxsid50194605 or e7080 or e-7080 or ee083865g2 or en300-7418350 or er203492-00 or er-203492-00 or ex-a249 or ft-0700727 or gtpl7426 or hms3244a07 or hms3244a08 or hms3244b07 or hms3654a14 or hy-10981 or j-513372 or kisplyx$2 or l01xe29 or lenvatinib$2 or lenvatinibum$2 or lenvima$2 or lev$2 or mfcd16038644 or mk7902 or mk-7902 or mls006011239 or ncgc00263198-01 or ncgc00263198-04 or ncgc00263198-07 or ns00069283 or nsc755980 or nsc-755980 or nsc800781 or nsc-800781 or q6523413 or ro7071618 or ro-7071618 or s1164 or sb16580 or schembl864638 or smr004702999 or sw219259-1 or z2235801899).ti,ab,kw. [LENVATINIB TERMS] | 6247099 |
| 310 | (0rf or 1001264-89-6 or 524y3ib4hq or ac-28420 or akos025396463 or as-17027 or bcp0726000195 or bcp9000712 or bdbm50398379 or ccg-269312 or chebi-95089 or chembl2177390 or cs-0975 or d10641 or db11743 or dtxsid101025595 or ex-a2077 or gdc0068 or gdc-0068 or gdc0068-di-hcl or gdc-0068-di-hcl or gtpl7887 or hy-15186 or ipatasertib$2 or mfcd22124514 or ncgc00346714-01 or ns00072927 or nsc767898 or nsc-767898 or nsc781451 or nsc-781451 or nsc800986 or nsc-800986 or nsc832484 or nsc-832484 or q27078088 or rg7440 or rg-7440 or rg-7440-di-hcl or s2808 or schembl191659).ti,ab,kw. [IPATASERTIB TERMS] | 724 |
| 311 | or/259-298 [INTERVENTION & COMPARATORS & CHEMO TERMS] | 3228584 |
| 312 | 258 and 311 | 35786 |
| 313 | (Adolescent/ or exp Child/ or exp Infant/) not (exp Adult/ and (Adolescent/ or exp Child/ or exp Infant/)) [CHILDREN <19 REMOVE] | 4816395 |
| 314 | (editorial or note or comment or clinical trial protocol).pt. or (letter.pt. not randomized controlled trial/) [PROTOCOLS and OPINION PIECES REMOVE - CENTRAL] | 5802227 |
| 315 | 312 not (313 or 314) [PROTOCOLS and OPINION PIECES REMOVED - CENTRAL] | 34194 |
| 316 | Conference proceeding.pt. [CONFERENCE ABSTRACTS/PROCEEDINGS] | 233853 |
| 317 | 315 and 316 [CONFERENCE ABSTRACTS ONLY] | 718 |
| 318 | limit 317 to yr="2022 -Current" | 89 |
| 319 | 315 not 316 [CONFERENCE ABSTRACTS REMOVED] | 33476 |
| 320 | 318 or 319 [LAST 2 YRS OF ABSTRACTS RETAINED] | 33565 |
| 321 | 320 use cctr [CENTRAL results] | 1778 |
| 322 | ((((stomach? or gastric$ or cardia or cardiac or antrum? or antral$ or fundus$ or pyloric$ or pylorus$ or ventricul$ or linitis plastica or leather-bottle or ((stomach? or gastric$) and (GC or GEJ))) adj3 (neoplas$ or cancer$ or tumo?r$ or carcinoma$ or malignan$ or oncolog$ or adenocancer$ or adeno-cancer$ or adenoma$ or adenocarcinoma$ or adeno-carcinoma$ or blastoma$ or carcinosarcoma$ or carcino-sarcoma$ or adenoacanthoma$ or adeno-acanthoma$ or epithelioma$ or melanoma$ or mesenchymoma$ or sarcoma$ or thymoma$ or granuloma$ or choriocarcinoma$ or cancerogenes?s or carcinoid$)) or ((stomach? or gastric$) adj3 SCC) or ((esophag$ or oesophag$ or esophagogastric$ or esophago-gastric$ or oesophagogastric$ or oesophago-gastric$ or gastroesophageal$ or gastro-esophageal$ or gastrooesophageal$ or gastro-oesophageal$ or cardio-esophageal$ or cardio-oesophageal$ or cardioesophageal$ or cardiooesophageal$ or EG or GE) adj3 (junction$ or sphincter$) adj3 (neoplas$ or cancer$ or tumo?r$ or carcinoma$ or malignan$ or oncolog$ or adenocancer$ or adeno-cancer$ or adenoma$ or adenocarcinoma$ or adeno-carcinoma$ or blastoma$ or carcinosarcoma$ or carcino-sarcoma$ or adenoacanthoma$ or adeno-acanthoma$ or epithelioma$ or melanoma$ or mesenchymoma$ or sarcoma$ or thymoma$ or granuloma$ or choriocarcinoma$ or cancerogenes?s or carcinoid$))) adj4 ((meta adj sta$) or metastas$ or metastatic$ or recur$ or secondar$ or relaps$ or advance$ or inoperab$ or disseminat$ or spread or migration or lethal$ or incurable or noncurable or non-curable or uncurable or progressive or terminal or invasive$ or aggressive$ or (late? adj2 stage$) or ((stage? or grade? or type?) adj2 (3a$ or 3b$ or 3c$ or III$ or 4a$ or 4b$ or IV$)) or "stage 3" or "stage 4" or met or mets or N1? or N2? or N3? or pN1? or pN2? or pN3?)).ti,ab,kw. [Metastatic GC/GEJ TERMS] | 71583 |
| 323 | (tislelizumab$2 or tirelizumab$2 or bgb-a317 or bgba317 or bgn-1 or bgn1 or jhl-2108 or jhl2108 or vdt-482 or vdt482 or 1858168-59-8 or 0kvo411b3n).ti,ab,kw. [TISLELIZUMAB TERMS] | 1319 |
| 324 | ((immune$ adj3 checkpoint? adj3 (inhibit$ or block?)) or (((programmed adj3 death) or PD-1 or PD-1-PD-L1 or PDCD1) adj3 (ligand? or inhibit$ or block?)) or ((B7-H1 or B7H1 or "B7 homolog 1" or CD274 or CD273 or PDCD1LG1 or PDCD1LG2) adj3 (antigen? or protein?)) or ((Cytotoxic-T-Lymphocyte-Associated Protein-4 Inhibitor? or CTLA-4) adj3 (inhibit$ or block?)) or (ICI? and "Immun$ Checkpoint") or BMS-1 or EX-A947 or HY-19991 or J-690233 or MFCD28978741 or s7911 or D000082082 or SCHEMBL16555159 or ZINC230477930 or 1675201-83-8).ti,ab,kw. [IMMUNE CHECKPOINT PROTEINS TERMS] | 114376 |
| 325 | (immunotherap$ or immuno-therap$ or (((biologic$ adj3 response? adj3 modifier?) or BRM or immunogenic$ or immunologic$ or immuno-genic$ or immuno-logic$ or radioimmunotherapy$ or radio-immunotherap$ or ((monoclonal$ or clonal$ or hybridoma$) adj2 antibod$)) adj3 (therap$ or intervention? or treat$))).ti,ab,kw. [IMMUNOTHERAPY TERMS] | 435716 |
| 326 | ((molecular$ or neoplas$ or cancer$ or tumo?r$ or carcinoma$) adj3 (target$ adj3 therap$)).ti,ab,kw. [TARGETED THERAPY TERMS] | 111847 |
| 327 | (atezolizumab$2 or anti-PDL1 or MPDL-3280A or MPDL3280A or RG-7446 or RG7446 or ro-5541267 or ro5541267 or Tecentriq$2 or Tecntriq$2 or 1380723-44-3 or 0INE2SFD9E or 52CMI0WC3Y).ti,ab,kw. [ATEZOLIZUMAB TERMS] | 12948 |
| 328 | (avelumab$2 or bavencio$2 or msb-0010682 or msb-0010718c or msb0010682 or msb0010718c or msb-10682 or msb-10718c or msb10682 or msb10718c or pf-06834635 or pf-6834635 or pf06834635 or pf6834635 or KXG2PJ551I or 1537032-82-8).ti,ab,kw. [AVELUMAB TERMS] | 3518 |
| 329 | (camrelizumab$2 or "anti-pd-1 monoclonal antibody" or shr-1210 or shr1210 or carilizumab$2 or carrelizumab$2 or 73096E137E or 1798286-48-2).ti,ab,kw. [CAMRELIZUMAB TERMS] | 2922 |
| 330 | (1428935-60-7 or 28x28x9okv or anti-b7h1-monoclonal-antibody or durvalumab$2 or durvalumabum$2 or imfinzi$2 or l01xc28 or medi4736 or medi-4736).ti,ab,kw. [DURVALUMAB TERMS] | 7170 |
| 331 | (ipilimumab$2 or bms-734016 or bms734016 or cs-1002 or cs1002 or ibi-310 or ibi310 or mdx-ctla-4 or mdx-010 or mdx-101 or mdx010 or mdx101 or strentarga$2 or yervoy$2 or 6T8C155666 or 477202-00-9).ti,ab,kw. [IPILIMUMAB TERMS] | 18680 |
| 332 | (nivolumab$2 or bms-936558 or bms-986213 or bms-986298 or cmab819 or bms936558 or bms986213 or bms986298 or cmab-819 or mdx-1106 or mdx1106 or ono-4538 or ono4538 or opdivo$2 or opdualag$2 or 31YO63LBSN or 946414-94-4).ti,ab,kw. [NIVOLUMAB TERMS] | 33925 |
| 333 | (pembrolizumab$2 or keytruda$2 or lambrolizumab$2 or mk3475 or mk-1308a or mk-3475 or mk7684a or sch-900475 or sch900475 or "keylynk-010 component" or DPT0O3T46P or 1422183-02-5 or 1374853-91-4).ti,ab,kw. [PEMBROLIZUMAB TERMS] | 32744 |
| 334 | (2072873-06-2 or 8fu7fq8upk or ibi308 or ibi-308 or sintilimab$2 or tyvyt$2 or who-10801).ti,ab,kw. [SINTILIMAB TERMS] | 1605 |
| 335 | (1924598-82-2 or 8jxn261vva or js001 or js-001 or tab001 or tab-001 or teripalimab$2 or toripalimab$2 or treipril$2 or treprizumab$2 or tripleitriumab$2 or triprizumab$2 or tuoyi$2 or who-10820).ti,ab,kw. [TORIPALIMAB TERMS] | 1078 |
| 336 | (2079108-44-2 or 2226345-85-1 or 2y3t5if01z or aex1188 or aex-1188 or incmga00012 or incmga-00012 or incmga0012 or incmga-0012 or mga012 or mga-012 or retifanlimab$2 or zynyz$2).ti,ab,kw. [RETIFANLIMAB TERMS] | 120 |
| 337 | (2102192-68-5 or anti-pd-l1-monoclonal-antibody-kn035 or asc22 or asc-22 or envafolimab$2 or es1m06m6qh or kn035 or kn-035).ti,ab,kw. [ENVAFOLIMAB TERMS] | 104 |
| 338 | (2245725-04-4 or l62556gpxb or mgd013 or mgd-013 or tebotelimab$2).ti,ab,kw. [TEBOTELIMAB TERMS] | 36 |
| 339 | (2394841-59-7 or 6fyg1ds4nw or ak104 or ak-104 or cadonilimab$2 or who-11581).ti,ab,kw. [CADONILIMAB TERMS] | 86 |
| 340 | (2231029-82-4 or hlx10 or hlx-10 or s3gqz2k36v or serplulimab$2).ti,ab,kw. [SERPLULIMAB TERMS] | 132 |
| 341 | (2256084-03-2 or 90iqr2i6tr or cs1001 or cs-1001 or sugemalimab$2 or wbp315 or wbp-315 or wbp3155 or wbp-3155).ti,ab,kw. [SUGEMALIMAB TERMS] | 127 |
| 342 | (1496553-00-4 or claudiximab$2 or imab362 or imab-362 or tf5mpq8wgy or zolbetuximab$2).ti,ab,kw. [ZOLBETUXIMAB TERMS] | 254 |
| 343 | (1952272-74-0 or bemarituzumab$2 or fpa144 or fpa-144 or rjw23bq0kw).ti,ab,kw. [BEMARITUZUMAB TERMS] | 84 |
| 344 | (205923-56-4 or abp494 or abp-494 or c225 or c-225 or c225-03 or c-22503 or c-225-03 or cdp1 or cdp-1 or cetuximab$2 or cetuximabum$2 or ch225 or ch-225 or chimeric-anti-egfr-monoclonal-antibody or cmab009 or cmab-009 or ctp15 or ct-p15 or dtxsid0040830 or erbitux$2 or hsdb-7454 or imc225 or imc-225 or imcc225 or imcc-225 or imc-c225 or kl140 or kl-140 or l01xc06 or ly2939777 or ly-2939777 or mab-c225 or moab-c225 or nsc714692 or pqx0d8j21j or sti001 or sti-001).ti,ab,kw. [CETUXIMAB TERMS] | 27851 |
| 345 | (1133766-06-9 or metmab$2 or metma-b or ms1j9720wc or onartuzumab$2 or pro143966 or pro-143966 or pro-143996 or pro143996 or ro5490258 or ro-5490258).ti,ab,kw. [ONARTUZUMAB TERMS] | 347 |
| 346 | (51wew898ij or 872514-65-3 or amg102 or amg-102 or rilotumumab$2).ti,ab,kw. [RILOTUMUMAB TERMS] | 259 |
| 347 | (1518996-49-0 or 571045eim4 or andecaliximab$2 or gs5745 or gs-5745).ti,ab,kw. [ANDECALIXIMAB TERMS] | 135 |
| 348 | (444731-52-6 or 635702-64-6 or 790713-33-6 or 7rn5dr86ck or a19406 or a839572 or ab01273967-01 or ab01273967-02 or ab01273967-05 or ab01273967-06 or ac-8522 or akos005145819 or am20090659 or ar-270-43507999 or armala$2 or as-11066 or bcp01839 or bcp9001053 or bcpp000129 or bd164238 or bdbm26474 or brd-k74514084-003-02-7 or ccg-265010 or chebi-71219 or chembl477772 or cid-10113978 or cs-0269 or db06589 or dtxcid1028659 or dtxsid8048733 or en300-57325 or ex-a1241 or ft-0659928 or ft-0684794 or gtpl5698 or gw780604 or gw-780604 or gw-78603 or gw786034 or gw7-86034 or gw-786034 or gw786034b or gw-786034b or gw786034x or gw-786034x or hms3244c21 or hms3244c22 or hms3244d21 or hms3656l14 or hms3745g05 or hsdb-8210 or hy-10208 or indazolylpyrimidine-13 or jmc514632-compound-13 or kinome-3790 or mfcd11616589 or ncgc00188865-01 or ncgc00188865-02 or ncgc00188865-03 or ncgc00188865-10 or nsc752782 or nsc-752782 or nsc800839 or nsc-800839 or p-6706 or pazopanib$2 or pazopanibum$2 or q-101400 or q7157043 or s3012 or sb17290 or sb710468 or sb-710468 or sb710468a or sb-710468a or schembl588608 or sw218082-3 or tox21-113174 or tox21-113174-1 or votrient$2 or z1541638525).ti,ab,kw. [PAZOPANIB TERMS] | 7544 |
| 349 | (1218779-75-9 or 5s371k6132 or 811803-05-1 or ab01274807-01 or ab01274807-02 or ac-27461 or akos024464453 or amy21302 or apatinib$2 or ba175030 or bcp02840 or c76598 or ccg-268625 or chembl3186534 or cs-0003200 or d11288 or db14765 or ds-7455 or dtxsid601024366 or ex-a1794 or gtpl7648 or hy-13342a or mfcd21648511 or ncgc00249393-01 or ncgc00249393-08 or nsc772886 or nsc-772886 or nsc799333 or nsc-799333 or q27262801 or rivoceranib$2 or s5248 or sb16590 or schembl1814966 or yn968d1 or yn-968d1).ti,ab,kw. [RIVOCERANIB/APATINIB TERMS] | 3781 |
| 350 | (chemotherap$ or chemo-therap$ or carcinochemotherap$ or chemoradiotherap$ or chemoradiation? or radiochemotherap$ or carcino-chemotherap$ or chemo-radiotherap$ or chemo-radiation? or radio-chemotherap$).ti,ab,kw. [CHEMOTHERAPY TERMS] | 1491580 |
| 351 | (leucovorin$ or 6-s-leucovorin or 6s-leucovorin or acide folinique or akos015961207 or bdbm50039121 or bpbio1-000766 or bspbio-000696 or bspbio-002218 or brd-a75919782-238-01-8 or calcium folinate or chebi-15640 or chembl1679 or chembl69905 or citrovoeum-factor or citrovorum-factor or d93089 or divk1c-000222 or dtxsid0048216 or einecs-200-361-6 or en300-27068710 or folinate folinic-acid-sf or folinic acid or formyltetrahydrofolate or fusilev$2 or gtpl4816 or gtpl6690 or hsdb-6544 or hy-17556 or idi1-000222 or kbio1-000222 or kbio2-001339 or kbio2-003907 or kbio2-006475 or kbio3-001438 or kbiogr-000461 or kbioss-001339 or lencovorin$2 or leucal$2 or leukovorin$2 or leukovoran$2 or leucovorin$2 or levoleucovorin$2 or levo-leucovorin$2 or mfcd00867488 or ninds-000222 or nsc3590 or prestwick0-000738 or prestwick1-000738 or prestwick2-000738 or prestwick3-000738 or q45435667 or q573i9dvlp or s5790 or schembl10068238 or schembl8349 or sd-204098 or s-leucovorin$2 or sleucovorin$2 or spectrum2-000116 or spectrum3-000479 or spectrum4-000031 or spectrum5-000910 or spectrum-000859 or spbio-000132 or spbio-002635 or sbi-0051427-p003 or welcovorin$2 or "formyltetrahydropteroylglutamic acid" or Q573I9DVLP or 58-05-9).ti,ab,kw. [LEUCOVORIN TERMS] | 30362 |
| 352 | (Carboplatin$2 or blastocarb$2 or boplatex$2 or carboplat$ or carbosin$2 or carbotec$2 or carplan$2 or CBDCA or (platinum adj3 (cis or diamin? or cyclobutanedicarboxylat? or dicarboxylatediammine)) or Dicarboxylatoplatinum or diamminecyclobutanedicarboxylatoplatinum or cycloplatin$ or erbakar$2 or ercar$2 or ifacap$2 or jm8 or jm-8 or kemocarb$2 or nsc-241240 or nsc241240 or nsc-201345 or nsc201345 or oncocarbin$2 or paraplatin$ or Platinwas$2 or Ribocarbo$2 or Neocarbo$2 or Nealorin$2 or HSDB-6957 or BG3F62OND5 or 41575-94-4).ti,ab,kw. [CARBOPLATIN TERMS] | 67440 |
| 353 | (paclitaxel$ or abraxane$2 or abraxus$2 or act02709 or act-02709 or acon1-002231 or anx-513 or anzatax$2 or apealea$2 or asotax$2 or bidd-pxr0046 or biotax$2 or bms-181339 or bms181339-01 or bms181339 or bms-181339-01 or bmy-45622 or bmy45622 or bspbio-000290 or capxol$2 or ccris-8143 or chembl428647 or chebi-45863 or coroflex-please$2 or coroxane$2 or cmap-000068 or cynviloq$2 or cypher-select$2 or dsstox-cid-3413 or dsstox-gsid-23413 or dsstox-rid-77016 or dhp107 or dhp-107 or dhp-208 or dhp208 or dts-301 or dts301 or ebetaxel$2 or empac$2 or endotag-1 or endotag1 or formoxol$2 or genaxol$2 or genetaxyl$2 or genexol$2 or gtpl2770 or hms2090d07 or hms2095o12 or hms2231a16 or hms3712o12 or hsdb-6839 or hunxol$2 or hy-b0015 or ifaxol$2 or ig-001 or ig001 or infinnium$2 or intaxel$2 or kbiogr-002509 or kbio2-002509 or kbio2-005077 or kbio2-007645 or kbio3-002987 or lep-etu$2 or lipopac$2 or liporaxel$2 or mbt-0206 or mbt0206 or medixel or mfcd00869953 or mitotax$2 or nanopac$2 or nanotax$2 or nanotaxel$2 or ncgc00164367-01 or nk-105 or nk105 or nsc-125973 or nsc-673089 or nsc125973 or nsc673089 or nscc-125973 or nova-12005 or oas-pac-100 or oaspac100 or oncogel$2 or onxal$2 or onxol$2 or p-ssmm-vip$2 or paclical$2 or pacitaxel$2 or paclical$2 or padexol$2 or pacligel$2 or paclitaxel$2 or pacliex$2 or paxceed$2 or paxene$2 or paxoral$2 or paxus$2 or pazenir$2 or plaxicel$2 or praxel$2 or qw-8184 or schembl3976 or sb-05 or sb05 or sdp-013 or sindaxel$2 or smr000857385 or sr-01000075350 or taycovit$2 or taxalbin$2 or taxane$ or taxocris$2 or taxol$2 or taxus$2 or tocosol$2 or xorane$2 or yewtaxan$2 or zinc96006020 or zisu$2 or P88XT4IS4D or 33069-62-4).ti,ab,kw. [PACLITAXEL TERMS] | 150988 |
| 354 | (114915-20-7 or 114977-28-5 or 15h5577cqd or 699121phca or ab01273941-01 or ab01273941-02 or ac-383 or akos015960718 or akos024457953 or amy4356 or anx-514 or axtere$2 or bd164373 or bdbm36351 or bind014 or bind-014 or brd-k30577245-001-04-3 or brd-k30577245-341-01-9 or bs102 or bs-102 or chebi-4672 or chembl92 or cid148124 or ckd-810 or crlx301 or crlx-301 or cs-1144 or d07866 or d4102 or daxotel$2 or db01248 or dexotel$2 or docecad$2 or docefrez$2 or docetaxel$2 or docetaxelum$2 or docetaxol$2 or docetaxolum$2 or dtxcid8020464 or dtxsid0040464 or emdoc$2 or en300-123047 or ex-a1206 or gtpl6809 or hms2089k08 or hsdb-6965 or hy-b0011 or ks-1452 or l01cd02 or lit976 or lit-976 or mfcd00871399 or ncgc00181306-01 or ncgc00181306-02 or ncgc00181306-04 or ncgc00242509-01 or nsc628503 or nsc-628503 or nsc-759850 or oncodocel$2 or q-100074 or q420436 or rp56976 or rp-56976 or schembl4419 or sdp-014 or sid-530 or sr-01000003023 or sr-01000003023-5 or syp-0704a or taxanit$2 or taxespira$2 or taxoel$2 or taxoltere-metro or taxotel$2 or taxoter$2 or taxotere$2 or texot$2 or tox21-112781 or tox21-113088 or txl$2 or w-60384 or xrp6976 or xrp-6976 or xrp-6976l or z1546621742).ti,ab,kw. [DOCETAXEL TERMS] | 62503 |
| 355 | (Cisplatin$ or platinum$ or Cismaplat$2 or (cis adj3 ($platinum or platinous)) or cis-platinum or cis-Platin or dichloroplatinum or diaminodichloroplatinum or diamminedichloroplatinum or dichlorodiammineplatinum or AI3-62048 or abiplatin or biocisplatinum or biocysplatinum or blastolem$2 or briplatin$2 or cddp-ti or cis-ddp or cisPt$ or CACP or CCRIS-221 or CDDP or DDPt or CP-Ethypharm or CPDC or CPDD or CPPD or (DDP and antitumor) or cisplatyl$2 or citoplatino$2 or cytoplatin$2 or cytosplat$2 or docistin$2 or elvecis$2 or kemoplat$2 or Fauldiscipla$2 or IA-call or LiPlaCis$2 or lederplatin$2 or lipoplatin$2 or "liposomal cisplatin" or mpi-5010 or mpi5010 or neoplatin$2 or niyaplat$2 or nk-801 or noveldexis$2 or nsc-119875 or nci-c55776 or platamine$2 or platamine-rtu or platiblastin$2 or platidiam$2 or platimine$2 or platinex$2 or platinil$2 or platino$2 or platinol$2 or platinolaq$2 or platinol-aq$2 or platinoxan$2 or platiran$2 or platistil$2 or platistin$2 or platosin$2 or "pronto platamine" or "Peyrone's chloride" or randa$2 or romcis$2 or sicatem$2 or spi-077 or tr-170 or tecnoplatin$2 or Q20Q21Q62J or 15663-27-1 or 26035-31-4 or 96081-74-2).ti,ab,kw. [CISPLATIN TERMS] | 328620 |
| 356 | (oxaliplatin$2 or (Oxalat$ adj3 platin$) or axiplatin$2 or bendaplatin$2 or crisapla$2 or croloxat$2 or dacotin$2 or dacplat$2 or ebeoxal$2 or elatofen$2 or eloxatin$ or elplat$2 or euroxaliplatin$2 or geneplatin$2 or gessedil$2 or heloxatin$2 or lipoxal$2 or mbp-426 or mbp426 or medoxa$2 or oksaliplatin$ or oplat$2 or oxalatoplatin$ or oxalatplatin$2 or oxali$2 or oxalip$2 or oxaliplan$2 or oxaliprol$2 or oxaliquid$2 or oxalisan$2 or oxalisin$2 or oxalizor$2 or oxaltic$2 or oxaltin$2 or oxamed$2 or oxaplamyl$2 or oxaviatin$2 or platox$2 or plaxitin$2 or rectoxal$2 or riboxatin$2 or rp-54780 or rp54780 or sinoxal$2 or sr-96669 or sr96669 or transplastin$2 or velminox$2 or xaliplat$2 or xoplan$2 or L-OHP-Cpd or 1-OHP or ACT-078 or ACT078 or CCRIS-9143 or NSC-266046 or 04ZR38536J or 61825-94-3).ti,ab,kw. [OXALIPLATIN TERMS] | 56812 |
| 357 | (capecitabin$ or apecitab$2 or atubri$2 or bc164277 or bcpp000300 or bxeliri$2 or bs-1000 or cacit$2 or capcel$2 or capebina$2 or capecite$2 or capegard$2 or capezam$2 or capicet$2 or capiri$2 or capiibine$2 or captabin$2 or capnat$2 or capoda$2 or capostat$2 or capsy$2 or capxcel$2 or caxeta$2 or ccg-264841 or ccx-340 or cpecitabine$2 or cs-0768 or d01223 or db01101 or dsstox-cid-26451 or dsstox-gsid-46451 or dsstox-rid-81625 or dtxsid3046451 or ecansya$2 or ex-a835 or gtpl6799 or hsdb-7656 or hy-b0016 or j-700154 or k007 or m0297 or mfcd00930626 or mls003915642 or mls004774137 or ncgc00164569-01 or ncgc00164569-02 or ncgc00164569-05 or nsc-759853 or paxon$2 or q-200788 or q420207 or r-340 or rg-340 or r340 or rg340 or ro-09-1978 or ro-091978 or ro-09-1978 or ro-09-1978-000 or ro091978 or s1156 or s-1156 or sr-01000931255 or tox21-112198 or x-abine$2 or x-tabin$2 or xabine$2 or xecap$2 or xeliri$2 or xelocel$2 or xeloda$2 or xelox$2 or z1501480421 or zinc3806413 or 6804dj8z9u or 154361-50-9 or 958887-39-3).ti,ab,kw. [CAPECITABINE TERMS] | 31460 |
| 358 | (platinum adj1 (fluoropyrimidine or fluoro-pyrimidine) adj3 (doublet? or combin$ or chemotherap$ or chemo-therap$ or ((first or front) adj1 line?) or 1-LOT or 1L or therap$ or regimen? or expos$)).ti,ab,kw. [PLATINUM-FLUOROPYRIMIDINE DOUBLET TERMS] | 253 |
| 359 | (5-fluoropyrimidin$ or 5-fluoro-pyrimidine or pyrimidine-5-fluoro or (fluorinated adj1 pyrimidine) or a9048 or ac-453 or akos006346044 or am86123 or "bb 0260992" or c4h3fn2 or db-007051 or dtxsid80217851 or en300-6966105 or f14737 or ft-0601423 or mfcd06658278 or q42859845 or w-203496 or zinc1845840 or 675f218 or L36X4TD47C or 675-21-8).ti,ab,kw,kf,ot,hw,rn,nm. [FLUOROPYRIMIDINE TERMS] | 5169 |
| 360 | (fluorouracil$ or fluroblastin$ or 1upf or 5-Faracil or 5-Fluoracil or 5-Fluoracyl or 5-fluoro-uracil or 5-fluoro-uracil or 5-Fluoroblastin or 5-fluorouacil or 5-Ftouracyl or 5-FU or 5FU or 5F-uracil or Adrucil$2 or AI3-25297 or Arumel$2 or BSPBio-002048 or Cancersil$2 or Carac$2 or Carzonal$2 or CHEBI-46345 or CHEMBL185 or Cinco-FU or CCRIS-2582 or DSSTox-CID-634 or DSSTox-GSID-20634 or DSSTox-RID-75705 or Efudex$2 or Efudix$2 or Efurix$2 or EINECS-200-085-6 or Effluderm$2 or Fluoroblastin$2 or Fluoro-Uracil$2 or Fluoro-uracile$2 or Fluoro-uracilo$2 or Fluoroplex$2 or Fluorouracile$2 or Fluorouracilo$2 or Fluorouracilum$2 or Fluorouracilum$2 or Fluracil$2 or Fluracilum$2 or Fluri$2 or Fluril$2 or Fluuro-Uracil$2 or Fluorouracilo$2 or Fluroblastin$2 or Fluro-Uracil$2 or Ftoruracil$2 or GTPL4789 or HSDB 3228 or IDI1-000054 or Kecimeton$2 or KBio1-000054 or KBio2-001321 or KBio2-003889 or KBio2-006457 or KBioGR-001253 or KBioSS-001321 or Lopac-F-6627 or Lopac0-000536 or MFCD00006018 or MLS000069498 or MLS002415705 or NCGC00015442-01 or NCGC00015442-02 or NCGC00015442-03 or NCGC00015442-04 or NCGC00015442-05 or NCGC00015442-06 or NCGC00015442-07 or NCGC00015442-08 or NCGC00015442-09 or NCGC00015442-10 or NCGC00015442-11 or NCGC00015442-12 or NCGC00015442-15 or NCGC00015442-16 or NSC-19893 or NSC757036 or NSC816997 or Phtoruracil$2 or Pharmakon1600-01500305 or Phthoruracil$2 or Queroplex$2 or Ro-2-9757 or S1209 or 191047-64-0 or 191047-65-1 or 191115-88-5 or U3P01618RT or 51-21-8).ti,ab,kw. [FLUOROURACIL TERMS] | 133662 |
| 361 | (irinotecan$ or ab00698464-07 or ab00698464-09 or ab00698464-10 or ab00698464-11 or ab00698464-12 or ab00698464-13 or ab00698464-14 or ac-7469 or akos015894969 or amy4227 or as-14323 or bdbm50128267 or bcp02860 or bcp9000793 or biotecan$2 or brd-k08547377-003-02-4 or campto$2 or camptosar$2 or chebi-80630 or chembl481 or cs-1138 or cpt-11 or cpt11 or d08086 or db00762 or dq2805 or en300-708800 or gtpl6823 or hsdb-7607 or ihl-305 or ihl305 or irinophore-c$2 or irinotel$2 or mfcd00866307 or ncgc00178697-02 or ncgc00178697-05 or nsc-728073 or nsc728073 or nk012-compound or q412197 or s1198 or schembl4034 or sn38 or sn-38 or sn-38-11 or sn3811 or topotecin$2 or u-101440e or u101440e or zinc1612996 or "7673326042" or 100286-90-6 or 97682-44-5).ti,ab,kw. [IRINOTECAN TERMS] | 38353 |
| 362 | (teysuno$2 or (tegafur adj4 gimeracil adj4 oteracil) or ((S-1 or S1) adj3 combination) or TS-1-cpd or S-1-cpd or TS-1 or TS1 or BMS247616 or BMS-247616 or S1-tegafur-oxonate or S1-fluoropyrimidine-oxoonate).ti,ab,kw. [S1 COMBINATION TERMS] | 7916 |
| 363 | (1189456-27-6 or 1548r74nsz or 17902-23-7 or 82294-77-7 or a812417 or ab00572620-15 or ac-2112 or akos000121279 or as-13528 or atillon$2 or bcp22714 or bp-58663 or brn-0525766 or c8h9fn2o3 or ccg-100959 or ccg-50110 or ccris-2762 or chebi-32188 or chembl20883 or citofur$2 or coparogin$2 or cs-1128 or d01244 or db09256 or dtxsid001009966 or einecs-241-846-2 or en300-21668 or exonal$2 or f-5-fu or fental$2 or florafur$2 or fluorafur$2 or fluorofur$2 or franrose$2 or franroze$2 or ft-0653732 or ft-0654170 or ft-0674829 or ft-0693965 or ft207 or ft-207 or ftorafur$2 or fulaid$2 or fulfeel$2 or furafluor$2 or furflucil$2 or furofutran$2 or futraful$2 or gtpl10513 or hms1665i05 or hms2051b15 or hms2090k04 or hms2232e05 or hms3371h21 or hms3393b15 or hms3654p13 or hms3715d14 or hy-17400 or lamar$2 or lifril$2 or mfcd00012351 or mjf12264 or mjf-12264 or mls000069497 or mls000759414 or mls001076521 or mls001424119 or nc00209 or ncgc00159418-02 or ncgc00159418-04 or ncgc00159418-05 or neberk$2 or nitobanil$2 or nsc148958 or nsc-148958 or opera-id-1726 or phthorafur$2 or q-201784 or q413370 or racemic-ftorafur or riol$2 or schembl4552 or sfsp$2 or sf-sp or sinoflurol$2 or smr000059106 or sr-01000639511 or sr-01000639511-1 or sr-01000639511-4 or sunfral$2 or sunfural$2 or tefsiel-c or tegaful$2 or tegafur$2 or tegafurum$2 or ts-1 or uftoral$2 or upcmld-dp063 or utefos$2 or z104508106).ti,ab,kw. [TEGAFUR TERMS] | 12581 |
| 364 | (1121b or 947687-13-0 or 947687-13-0 or a168 or a-168 or cyramza$2 or d99yvk4l0x or hlx12 or hlx-12 or hsdb-8314 or imc1121b or imc1121-b or imc-1121b or imc-1121-b or l01xc21 or ly3009806 or ly-3009806 or nsc-749128 or pbp2001 or pbp-2001 or ramucirumab$2 or ramucirumabum$2 or ro7234952 or ro-7234952).ti,ab,kw. [RAMUCIRUMAB TERMS] | 6008 |
| 365 | (339177-26-3 or 6a901e312a or abenix$2 or abx-egf or amg954 or amg-954 or e7-6-3 or l01xc08 or moab-abx-egf or moab-e7-6-3 or monoclonal-antibody-abx-egf or monoclonal-antibody-e7-6-3 or nsc-742319 or panitumab$2 or panitumumab$2 or panitumumabum$2 or panitunumab$2 or vectibex$2 or vectibix$2).ti,ab,kw. [PANITUMUMAB TERMS] | 6826 |
| 366 | (6ns400bxkh or 780758-10-3 or 828933-51-3 or biomab-egfr or diacim$2 or h-r3 or nimotuzumab$2 or osag-101 or radiotheracim$2 or theracim$2 or theraloc$2).ti,ab,kw. [NIMOTUZUMAB TERMS] | 1470 |
| 367 | (339186-68-4 or emd7200 or emd-7200 or emd72000 or emd-72000 or kgaa$2 or matuzumab$2 or merck-kgaa or mg4m3qb242).ti,ab,kw. [MATUZUMAB TERMS] | 50610 |
| 368 | (12-igg1 or 1438851-35-4 or 216974-75-3 or 2s9zzm9q9v or abevmy$2 or abp215 or abp-215 or ainex$2 or altuzan$2 or alymsys$2 or ankeda$2 or anti-vegf or askb1202 or ask-b1202 or avastin$2 or avegra$2 or aybintio$2 or ba1101 or ba-1101 or bambevi$2 or bat1706 or bat-1706 or bcd021 or bcd-021 or bevacizumab$2 or bevacizumabum$2 or bevagen$2 or bevatas$2 or bevax$2 or bevz92 or bevz-92 or bi695502 or bi-695502 or bow030 or bow-030 or boyounuo$2 or bp01 or bp-01 or bp102 or bp-102 or bryxta$2 or bs503a or bs-503a or bxt2316 or bxt-2316 or byvasda$2 or cbt124 or cbt-124 or chs305 or chs-305 or chs5217 or chs-5217 or cizumab$2 or ctp16 or ct-p16 or equidacent$2 or fkb238 or fkb-238 or gb222 or gb-222 or gbs004 or gbs-004 or hanbeitai$2 or hd204 or hd-204 or hlx04 or hlx-04 or hot1010 or hot-1010 or hsdb-8080 or ibi305 or ibi-305 or idb0072 or idb-0072 or intp24 or intp-24 or ipique$2 or jhl1149 or jhl-1149 or js501 or js-501 or jy028 or jy-028 or krabeva$2 or kyomarc$2 or l01xc07 or lextemy$2 or "lumiere-(drug)" or ly01008 or ly-01008 or mabionvegf$2 or mb02 or mb-02 or mil60 or mil-60 or mvasi$2 or myl14020 or myl-14020 or myl1402o or myl-1402o or nsc704865 or nsc-704865 or onbevzi$2 or ons1045 or ons-1045 or ons5010 or ons-5010 or oyavas$2 or pf06439535 or pf-06439535 or pf6439535 or pf-6439535 or pmc901 or pmc-901 or pobevcy$2 or pro169 or pro-169 or pusintin$2 or ql1101 or ql-1101 or r435 or r-435 or rg435 or rg-435 or rhumab$2 or ro4876646 or ro-4876646 or rph001 or rph-001 or rtpr023 or r-tpr-023 or sb8 or sb-8 or sct501 or sct-501 or sct510 or sct-510 or sibp04 or sibp-04 or stc103 or stc-103 or stivant$2 or tab008 or tab-008 or tab014 or tab-014 or tot102 or tot-102 or trs003 or trs-003 or tx16 or tx-16 or vegzelma$2 or versavo$2 or zirabev$2 or zrc113 or zrc-113 or zybev$2).ti,ab,kw. [BEVACIZUMAB TERMS] | 87453 |
| 369 | (2022215-59-2 or anb011 or anb-011 or dostarlimab$2 or gsk4057190 or gsk-4057190 or jemperli$2 or p0gvq9a4s5 or tsr042 or tsr-042 or wbp285 or wbp-285).ti,ab,kw. [DOSTARLIMAB TERMS] | 493 |
| 370 | (chembl5095383 or retlirafusp-alfa or shr1701 or shr-1701).ti,ab,kw,kf,ot,hw,rn,nm. [SHR-1701 TERMS] | 56 |
| 371 | (2368219-35-4 or 45x7ou8c4j or ab154 or ab-154 or domvanalimab$2 or who-11559).ti,ab,kw. [DOMVANALIMAB TERMS] | 49 |
| 372 | (2259860-24-5 or ab122 or ab-122 or gls010 or gls-010 or gs0122 or gs-0122 or wbp3055 or wbp-3055 or who-11413 or zbl7o904il or zimberelimab$2).ti,ab,kw. [ZIMBERELIMAB TERMS] | 126 |
| 373 | (0vua21238f or 1092929-10-6 or 1210608-87-9 or 1xkk or 231277-92-2 or 388082-78-8 or 437755-78-7 or 913989-15-8 or a25184 or ab01273965-01 or ab01273965-02 or ab01273965-03 or ab01273965-04 or ab01273965-05 or ac-1314 or akos005145766 or am20090641 or as-14065 or bc164610 or bcp01874 or bcp9000837 or bcp9000838 or bcpp000188 or bcpp000189 or bdbm5445 or brd-k19687926-001-01-7 or brd-k19687926-379-02-5 or c29h26clfn4o4s or ccg-270133 or chebi-49603 or chembl554 or cid-208908 or d08108 or db01259 or dtxcid5026675 or dtxsid7046675 or en300-117254 or ex-a402 or fmm$2 or ft-0659650 or gsk572016 or gsk-572016 or gtpl5692 or gw2016 or gw-2016 or gw282974x or gw-282974x or gw572016 or gw-572016 or gw572016f or gw-572016f or gw-572016x or hms2089h10 or hms3244n06 or hms3244n10 or hms3244n14 or hms3744k11 or hsdb-8209 or hy-50898 or kinome-3684 or kinome-3685 or l0360 or lapatinib$2 or mfcd09264194 or ncgc00167507-01 or ncgc00167507-02 or ncgc00167507-03 or ncgc00167507-04 or ncgc00167507-09 or ns00003012 or nsc745750 or nsc-745750 or nsc800780 or nsc-800780 or q-101353 or q420323 or sb16918 or schembl8100 or sr-05000001472-1 or sw199101-5 or tox21-112505 or tykerb$2 or tyverb$2).ti,ab,kw. [LAPATINIB TERMS] | 11804 |
| 374 | (857890-39-2 or a825653 or ac-25047 or aiv007 or aiv-007 or akos025401742 or amy9240 or as-16203 or bcp01799 or bcp9000633 or bcpp000247 or bdbm50331094 or bl164616 or c21h19cln4o4 or ccg-264842 or chebi-85994 or chembl1289601 or cs-0109 or d09919 or db09078 or dtxcid50117096 or dtxsid50194605 or e7080 or e-7080 or ee083865g2 or en300-7418350 or er203492-00 or er-203492-00 or ex-a249 or ft-0700727 or gtpl7426 or hms3244a07 or hms3244a08 or hms3244b07 or hms3654a14 or hy-10981 or j-513372 or kisplyx$2 or l01xe29 or lenvatinib$2 or lenvatinibum$2 or lenvima$2 or lev$2 or mfcd16038644 or mk7902 or mk-7902 or mls006011239 or ncgc00263198-01 or ncgc00263198-04 or ncgc00263198-07 or ns00069283 or nsc755980 or nsc-755980 or nsc800781 or nsc-800781 or q6523413 or ro7071618 or ro-7071618 or s1164 or sb16580 or schembl864638 or smr004702999 or sw219259-1 or z2235801899).ti,ab,kw. [LENVATINIB TERMS] | 6247099 |
| 375 | (0rf or 1001264-89-6 or 524y3ib4hq or ac-28420 or akos025396463 or as-17027 or bcp0726000195 or bcp9000712 or bdbm50398379 or ccg-269312 or chebi-95089 or chembl2177390 or cs-0975 or d10641 or db11743 or dtxsid101025595 or ex-a2077 or gdc0068 or gdc-0068 or gdc0068-di-hcl or gdc-0068-di-hcl or gtpl7887 or hy-15186 or ipatasertib$2 or mfcd22124514 or ncgc00346714-01 or ns00072927 or nsc767898 or nsc-767898 or nsc781451 or nsc-781451 or nsc800986 or nsc-800986 or nsc832484 or nsc-832484 or q27078088 or rg7440 or rg-7440 or rg-7440-di-hcl or s2808 or schembl191659).ti,ab,kw. [IPATASERTIB TERMS] | 724 |
| 376 | or/323-363 [INTERVENTION & COMPARATORS & CHEMO TERMS] | 2345868 |
| 377 | 322 and 376 | 32251 |
| 378 | 377 use coch [CDSR results] | 7 |
| 379 | 181 or 254 or 321 or 378 [All results - no date limit] | 8418 |

Supplemental Searches

Selected databases and grey literature for supplemental searches

| **Search Engine** | **Database** |
| --- | --- |
| ***Ovid*** | - Ovid MEDLINE^®^ - Ovid MEDLINE Epub Ahead of Print, In-Process & Other Non-Indexed Citations and Daily - Ovid Embase - EBM Reviews – Cochrane Central Register of Controlled Trials - EBM Ovid EBM Reviews – Cochrane Database of Systematic Reviews |
| ***Conferences*** | - ASCO - ASCO GI - AACR - CSCO - ISPOR - ISPOR EU - JGCA - KSMO - SEOM - WCGI |
| ***Other Sources*** | - ClinicalTrials.gov <https://www.clinicaltrials.gov/> - International Clinical Trials Registry Platform <https://www.who.int/clinical-trials-registry-platform> - Bibliographic search of select relevant SLRs |
| ***HTA Agencies*** | - PBAC - CADTH - NICE - SMC |

Abbreviations: *AACR* American Association for Cancer Research; *ASCO* American Society of Clinical Oncology; *ASCO GI* American Society of Clinical Oncology Gastrointestinal; *CADTH* Canadian Agency for Drugs and Technologies in Health; *CSCO* Chinese Society of Clinical Oncology; *EBM* evidence-based medicine; *HTA* Health Technology Assessment; *ICTRP* International Clinical Trials Registry Platform; *ISPOR* International Society of Pharmacoeconomics and Outcomes Research; *ISPOR* *EU* International Society of Pharmacoeconomics and Outcomes Research European Union; *JGCA* Japanese Gastric Cancer Association; *KSMO* Korean Society of Medical Oncology; *NICE* National Institute for Health and Care Excellence; *PBAC* Pharmaceutical Benefits Advisory Committee; *SEOM* Sociedad Espanola de Oncologia Medica; *SLR* Systematic literature review; *SMC* Scottish Medicines Consortium; *WCGI* World Congress on Gastrointestinal Cancer.

### Study Eligibility Criteria

Study Selection Criteria

|  | **Inclusion Criteria** | **Exclusion Criteria** |
| --- | --- | --- |
| ***Population*** | - 1L unresectable, locally advanced, or metastatic HER2 negative GC/GEJ adenocarcinoma - Adult patients (18+) | - 2L or later GC/GEJ adenocarcinoma - HER2+ patients - Other cancers - Pediatric patients (<18 years) |
| ***Interventions*** | - Immuno-oncology treatments (PD-1/PD-L1 inhibitors, immune checkpoint inhibitors, etc.) - Other targeted therapies (TKIs, EGFR inhibitors, VEGFR-2 inhibitors, CLDN18.2 inhibitors, FGFR2b inhibitors, etc.) - Chemotherapy | - Those not listed |
| ***Comparators*** | - Any intervention above (alone or in combination with chemotherapy/targeted therapy/any other immunotherapy) - Placebo | - Those not listed |
| ***Outcomes*** | - OS, PFS, ORR, DoR, HRQoL, AE | - Any study not including at least one eligible outcome |
| ***Study Design*** | - Phase 2 and Phase 3 RCTs - Full text articles from database inception to present | - Phase 1 and 4 RCTs - Non-randomized studies - Conference abstracts prior to 2022 |
| ***Date*** | - Full text publications (database inception to present) - Conference abstracts (2022 to 2024) | - Those not listed |
| ***Language*** | - English language articles | - Non-English articles |

Abbreviations: *1L* first line; *2L* second line; *AE* adverse events, *CLDN18.2* claudin 18 isoform 2; *CR* complete response; *DoR* duration of response; *EGFR* epidermal growth factor receptor; *FGFR2b* anti-fibroblast growth factor receptor-2 isoform IIb; *GC* gastric cancer; *GEJ* gastroesophageal junction adenocarcinoma; *HER2* human epidermal growth factor receptor 2; *HGF* hepatocyte growth factor; *HRQoL* health-related quality of life; *ORR* objective response rate; *OS* overall survival; *PD-1* programmed death protein 1; *PD-L1* programmed death-ligand protein 1; *PFS* progression-free survival; *RCT* randomized controlled trial; *TKI* tyrosine kinase inhibitor; *VEGFR-2* vascular endothelial growth factor receptor-2.

### Assessment of Study Quality

- A risk of bias assessment of each included trial was also conducted using the NICE Single Technology Appraisal Evidence Submission Checklist for assessment of risk of bias in RCTs.
- Results of the risk assessment for each include RCT are included in **Appendix Table** below.

Assessment of Study Quality Results

| **Trial; NCT** | **Was randomization carried out appropriately?** | **Was the concealment of treatment allocation adequate?** | **Were the groups similar to the outset of the study in terms of prognostic factors?** | **Were the care providers, participants, and the outcome assessors blind to treatment allocation?** | **Were there any unexpected imbalances in dropouts between groups?** | **Is there any evidence to suggest that the authors measured more outcomes than they reported?** | **Did the analysis include an ITT analysis? If so, was this appropriate and were appropriate methods used to account for missing data?** |
| --- | --- | --- | --- | --- | --- | --- | --- |
| ATTRACTION-4 Part 1;  NCT02746796 | Yes | Yes | Yes | No | No | No | Yes |
| ATTRACTION-4 Part 2;  NCT02746796 | Yes | Yes | Yes | Yes | No | No | Yes |
| CheckMate 649; (NCT02872116) | Yes | Yes | Yes | No | No | No | Yes |
| EXELOX | Yes | Unclear | Yes | No | No | No | Yes |
| EXPAND; (EudraCT number 2007-004219-75 | Yes | Yes | Yes | No | No | No | Yes |
| FAST;  NCT01630083 | Unclear | Yes | Yes | No | No | No | Yes |
| GAMMA-1;  NCT02545504 | Yes | Yes | Yes | Yes | No | No | Yes |
| GAPSO;  NCT03801668 | Yes | No | Yes | No | No | No | Yes |
| GLOW;  NCT03653507 | Yes | Yes | Yes | Yes | No | Yes | Yes |
| HERBIS-2;  UMIN000006105 | Yes | Unclear | Yes | No | No | No | Yes |
| Japic CTI, number 111635 | Yes | Unclear | Yes | No | No | No | Yes |
| JCOG1013;  UMIN000007652 | Yes | Unclear | Yes | No | No | No | Yes |
| JCOG1108/ WJOG7312G;  Nakajima 2020 | Yes | Yes | Yes | No | No | No | Yes |
| KCSG ST13-10;  NCT02114359 | Yes | No | Yes | No | No | No | No |
| KEYNOTE-062;  NCT02494583 | Yes | Yes | Yes | Yes | No | No | Yes |
| KEYNOTE-859;  NCT03675737 | Yes | Yes | Yes | Yes | No | No | Yes |
| LEGA/GISCAD;  NCT02076594/2011-005537-39 | Yes | Yes | Yes | No | No | No | Yes |
| METGastric;  NCT01662869 | Yes | Unclear | Yes | Yes | No | No | Yes |
| NA | No | No | Yes | No | Unclear | No | No |
| NCT00719550 | Yes | Unclear | Yes | Yes | No | No | Yes |
| NCT01283204 | Yes | Unclear | Yes | No | No | No | Unclear |
| NCT01896531 | Unclear | Unclear | Yes | Yes | No | No | Yes |
| NCT02445209 | Yes | Unclear | Yes | No | Unclear | No | Yes |
| NCT03472365 | No | No | Unclear | No | Yes | No | No |
| OGSG1105, HERBIS-4A;  UMIN000006755 | Unclear | Unclear | Yes | No | No | No | Unclear |
| PaFLO;  AIO-STO-0510 | No | Unclear | Yes | No | Unclear | No | Yes |
| RAINFALL;  NCT02314117 | Yes | Unclear | Yes | Yes | No | No | Yes |
| RAINSTORM;  NCT02539225 | Yes | Yes | Yes | Yes | No | No | Unclear |
| RATIONALE-305;  NCT03777657 | Yes | Yes | Yes | Yes | No | No | Yes |
| RILOMET-1;  NCT01697072 | Yes | Yes | Yes | Yes | No | Yes | Yes |
| SOLAR;  NCT02322593 | Yes | Yes | Yes | No | No | No | Unclear |
| SPOTLIGHT;  NCT03504397 | Yes | Yes | Yes | Yes | No | Yes | Yes |
| SYLT/FNF-004 | Yes | No | Yes | No | No | No | No |
| XParTS II;  NCT01406249 | Yes | Yes | Yes | No | No | No | Unclear |
| YO28252;  NCT01590719 | Yes | Yes | Yes | Yes | No | No | Yes |
