# Supplementary material for: Comparative efficacy and safety of tislelizumab and other programmed cell death protein 1 inhibitors in first-line treatment of advanced gastroesophageal cancers: a systematic review and network meta-analysis
Source: Gastric Cancer. 2025 Oct 4;28(6):1021–32. doi: 10.1007/s10120-025-01660-4 (PMC12630173; doi:10.1007/s10120-025-01660-4)
Supplement: Supplementary file 2 — Supplementary file2 (DOCX 169 KB) [file 10120_2025_1660_MOESM2_ESM.docx]

# Supplementary material

Online Resource #2 for Comparative Efficacy and Safety of Tislelizumab and other Programmed Cell Death Protein 1 Inhibitors in First-line Treatment of Advanced Gastroesophageal Cancers: A Systematic Review and Network Meta-Analysis

Jaffer A. Ajani^1^ • Maria Alsina ^2^ • Markus Moehler^3^ • Keun-Wook Lee^4^ • Wenxi Tang^5^ • Jason Steenkamp^6^ • Emily Prentiss^6^ • Kaijun Wang^5^ • Becky Hooper^6^ • Lin Zhan^5^

^1^ Department of Gastrointestinal Medical Oncology, Division of Cancer Medicine, The University of Texas MD Anderson Cancer Center, Houston, TX, USA

^2^ Medical Oncology Department, Unidad de Oncología Médica Traslacional, Hospital Universitario de Navarra, Navarrabiomed – IdiSNA, Pamplona, Spain

^3^ Gastrointestinal Oncology, Johannes Gutenberg-University Clinic, Mainz, Germany

^4^ Department of Internal Medicine, Seoul National University College of Medicine, Seoul National University Bundang Hospital, Seongnam, Republic of Korea

^5^BeOne Medicines, Ltd. San Carlos, CA, USA

^6^Value & Evidence Services, EVERSANA, Burlington, ON, Canada

*** Correspondence:**Jaffer A. Ajani
[jajani@mdanderson.org](mailto:jajani@mdanderson.org)

713-792-2828

## Online Resource 2 – Summary of Relevant Trials Identified in the SLR

Summary of Relevant Trials Evaluated in the Feasibility Assessment

| **Trial Name; NCT** | **Data Cutoff Date** | **Arms (n patients)** | **Median Follow-Up (months)^a^** |
| --- | --- | --- | --- |
| RATIONALE-305[1];  NCT03777657 | 28-Feb-23* | TIS + CT  (ITT = 501)  (safety = 498) | TIS + CT: 14.1  PBO + CT: 12.6 |
|  |  | PBO + CT  (ITT = 496)  (safety = 494) |  |
| ATTRACTION-4  (Part 2)[2];  NCT02746796 | Interim analysis for PFS: 31-Oct-18*  Final analysis for OS: 31-Jan-20* | NIV + CT  (ITT = 362)  (safety = 359) | Interim analysis for PFS: 11.6  Final analysis for OS: 26.6 |
|  |  | PBO + CT  (ITT = 362)  (safety = 358) |  |
| CheckMate 649[3];  NCT02872116 | 27-May-21* | NIV + CT  (ITT = 789)  (safety = 782) | NIV + CT: 13.1  CT : 11.2  NIV + IPI: 11.4  CT: 11.5 |
|  |  | CT  (ITT = 792)  (safety = 767) |  |
|  |  | NIV + IPI  (ITT = 409)  (safety = 403) |  |
|  |  | CT  (ITT = 404)  (safety = 389) |  |
|  | 27-May-20 | NIV + CT  (ITT = 789)  (safety = 782) | OS NIV + CT: 13.1  OS CT: 11.1 |
|  |  | CT  (ITT = 792)  (safety = 767) |  |
| MOONLIGHT[4];  NCT03647969 | NR | NIV + IPI + CT  (Arm A, ITT = 60)  (safety = 59) | 9.7 |
|  |  | CT  (Arm B, ITT = 60)  (safety = 60) |  |
| KEYNOTE-062[5];  NCT02494583 | 26-Mar-19* | PEM  (ITT = 256)  (safety = 254) | 29.4 |
|  |  | PEM + CT  (ITT = 257)  (safety = 250) |  |
|  |  | PBO + CT  (ITT = 250)  (safety = 244) |  |
|  | 19-Apr-21 | PEM  (ITT = 256)  (safety = 254) | 54.3 |
|  |  | PEM + CT  (ITT = 257)  (safety = 250) |  |
|  |  | PBO + CT  (ITT = 250)  (safety = 244) |  |
| KEYNOTE-859[6];  NCT03675737 | 3-Oct-22* | PEM + CT  (ITT = 790)  (safety = 785) | 31.0 |
|  |  | PBO + CT  (ITT = 789)  (safety = 787) |  |
|  | 1-Jul-22 | PEM + CT  (ITT = 149)  (safety = NR) | 25.8 |
|  |  | PBO + CT  (ITT = 185)  (safety = NR) |  |
| ORIENT-16[7];  NCT03745170 | 20-Jun-21 | SIN + CT  (ITT = 327)  (safety = 328) | Overall: 18.8  OS SIN + CT = 19.3  OS PBO + CT: 18.1 |
|  |  | PBO + CT  (ITT = 323)  (safety = 320) |  |
|  | 2-Sep-22 | SIN + CT  (ITT = NR)  (safety = NR) | 33.9 |
|  |  | PBO + CT  (ITT = NR)  (safety = NR) |  |
| GEMSTONE-303[8];  NCT03802591 | PFS: 6-Aug-2022 OS: 9-Jul-2023 | SUG + CT (ITT = 241)  (safety = 241) | NR |
|  |  | PBO + CT (ITT = 238)  (safety = 238) |  |
| GLOW[9];  NCT03653507 | 7-Oct-22 | ZOL + CT  (ITT = 254)  (safety = 254) | ZOL + CT  PFS: 12.6  OS: 17.7  PBO + CT  PFS: 12.1  OS: 18.4 |
|  |  | PBO + CT  (ITT = 253)  (safety = 249) |  |
|  | 29-Jun-23 | ZOL + CT  (ITT = 254)  (safety = NR) | ZOL + CT  PFS: 17.8  OS: 26.1  PBO + CT  PFS: 15.1  OS: 26.2 |
|  |  | PBO + CT  (ITT = 253)  (safety = NR) |  |
| SPOTLIGHT[10];  NCT03504397 | NR (primary analysis) | ZOL + CT  (ITT = 283)  (safety = 279) | ZOL + CT  PFS: 12.9  OS: 22.1  PBO + CT  PFS: 12.7  OS: 20.9 |
|  |  | PBO + CT  (ITT = 282)  (safety = 278) |  |
|  | 29-Jun-23 | ZOL + CT  (ITT = 283)  (safety = NR) | ZOL + CT  PFS: 17.9  OS: 31.1  PBO + CT  PFS: 15.2  OS: 29.6 |
|  |  | PBO + CT  (ITT = 282)  (safety = NR) |  |
| FAST[11];  NCT01630083 | 31-Jan-19 | ZOL + CT (1000 mg/m²)  (ITT = 85)  (safety = 85) | NR |
|  |  | ZOL + CT (800/600 mg/m²) (ITT = 77)  (safety = 77) |  |
|  |  | CT (ITT = 84)  (safety = 84) |  |
| FIGHT[12];  NCT03694522 | PFS: 23-Sept-20  OS: 28-Feb-21 | BEM + CT  (ITT = 77)  (safety = 76) | PFS: 10.9  OS: 12.5 |
|  |  | PBO + CT  (ITT = 214)  (safety = 77) |  |
|  | 13-May-22 | BEM + CT  (ITT = NR)  (safety = NR) | BEM + CT  OS: 19.2  PFS: 9.5  PBO + CT  OS: 13.5  PFS: 7.4 |
|  |  | PBO + CT  (ITT = NR)  (safety = NR) |  |

Pink shading indicates trials included in the NMA.

* Data cutoff used in base case NMA analyses presented in this manuscript. Final analysis data from RATIONALE-305 (data cutoff February 28^th^, 2023) were made available to the research team for inclusion in the NMA before their publication in Qiu et al., 2024 [1]. As the data are now publicly available, the Qiu et al. publication is cited in the manuscript and this table despite being published after the SLR was conducted. ^a^ There was cross-trial variability in the reporting of median follow-up times, with some trials reporting overall follow-up times for the trial as a whole and others reporting median follow-up times by treatment arm. Further, some trials reported median follow-up times for specific outcomes of interest.

Abbreviations: *BEM* bemarituzumab; *CT* chemotherapy; *ITT* intent-to-treat; *IPI* ipilimumab; *NIV* nivolumab; *NMA* network meta-analysis; *OS* overall survival; *PBO* placebo; *PEM* pembrolizumab; *PFS* progression-free survival; *SER* serplulimab; *SIN* sintilimab; *SLR* systematic literature review; *SUG* sugemalimab; *TIS* tislelizumab; *ZOL* zolbetuximab.

**Baseline Patient Characteristics in Trials included in NMA Base Case Analyses**

|  | **RATIONALE-305**  **(N = 997)** [1] | **ATTRACTION-4**  **(N=724)** [2] | **CheckMate 649 (N=1,581)^a^** [3] | **KEYNOTE-062**  **(N = 507)^b^** [5] | **KEYNOTE-859 (N=1,579)**[6] |
| --- | --- | --- | --- | --- | --- |
| **Median age** | TIS + CT: 60.0  PBO + CT: 61.0 | NIV + CT: 64  PBO + CT: 65 | NIV + CT: 62  CT: 61 | PEM + CT: 62.0  CT: 62.5 | PEM + CT: 61  PBO + CT: 62 |
| **Male, %** | 69.4 | 72.2 | 69.6 | 73.8 | 67.8 |
| **Geographic region, %** | | | | | |
| Asia | 75.0 | 100 | 22.5 | 24.7 | 33.2 |
| **ECOG PS, %** | | | | | |
| 0 | 32.4 | 53.7 | 41.9 | 46.2^c^ | 36.9 |
| **Disease at entry, %** | | | | | |
| Locally advanced | 1.1 | NR^d^ | 3.9 | NR | 3.7 |
| Metastatic | 98.7 | NR^d^ | 95.7 | 94.3 | 96.3 |
| Recurrent | 0.1 | NR^d^ | 0.4 | NR | NR |
| **Primary tumor location, %** | | | | | |
| Stomach | 80.2 | 65.6 | 70.2 | 69.2 | 78.7 |
| GEJ | 19.7 | 8.6 | 16.4 | 30.0 | 21.2 |
| Not available | - | 25.8 | - | - | <1 |
| Esophageal adenocarcinoma | - | - | 13.3 | - | - |
| **Organs with metastases by type, %** | | | | | |
| Peritoneum | 43.5 | 46.4 | 23.8 | NR | NR |
| Liver | 37.9 | 36.5 | 38.9 | NR | 39.6 |
| **PD-L1 (%)** | | | | | |
| ≥1% | 88.8 | 15.7 | 82.0 | 100 | 78.2 |
| ≥5% | 54.8 | NR | 60.4 | NR | 49.6^e^ |
| ≥10% | 27.1 | NR | 48.5 | 37.3 | 34.9 |
| Method used | TAP | NR | CPS | CPS | CPS |
| **Prior therapy, %** | | | | | |
| Surgery | 32.9 | 28.9 | 21.3 | NR | 21.2 |
| Radiotherapy | 1.5 | NR | NR | NR | NR |
| Adjuvant or neoadjuvant | 20.8 | 17.5 | 14.5 | NR | NR |

Note: The table presents the baseline characteristics of the ITT population (pooled data for the treatment arms of interest, i.e., immunotherapy plus chemotherapy and chemotherapy control).

^a^ Baseline characteristics in the nivolumab + CT arm (n = 789) and the corresponding CT control arm (n = 792).

^b^ Baseline characteristics in the pembrolizumab + CT arms (n = 257) and the CT control arm (n = 250).

^c^ Reported ECOG PS of 1 in 53.8% of participants, while ECOG PS for the remaining 46.2% of patients was not reported.

Per eligibility criteria, only patients with ECOG PS of 0 or 1 were included in the trial.

^d^ Reported as 77.2% advanced and 22.8% recurrent.

^e^ Data were not reported in the source publication, but were retrieved from an FDA Briefing Document on immune checkpoint inhibitors in patients with metastatic or unresectable HER2-negative gastric adenocarcinoma [13].

Abbreviations: *CPS* combined positive score; *CT* chemotherapy; *ECOG* Eastern Cooperative Oncology Group; *FDA* Food and Drug Administration; *ITT* intent-to-treat; *NIV* nivolumab; *NR* not reported; *PBO* placebo; *PD-L1* programmed death-ligand 1; *PEM* pembrolizumab; *TAP* Tumor Area Positivity; *TIS* tislelizumab.

# References

1. Qiu MZ, Oh DY, Kato K, Arkenau T, Tabernero J, Correa MC, et al. Tislelizumab plus chemotherapy versus placebo plus chemotherapy as first line treatment for advanced gastric or gastro-oesophageal junction adenocarcinoma: RATIONALE-305 randomised, double blind, phase 3 trial. BMJ. 2024;385:e078876.

2. Kang YK, Chen LT, Ryu MH, Oh DY, Oh SC, Chung HC, et al. Nivolumab plus chemotherapy versus placebo plus chemotherapy in patients with HER2-negative, untreated, unresectable advanced or recurrent gastric or gastro-oesophageal junction cancer (ATTRACTION-4): a randomised, multicentre, double-blind, placebo-controlled, phase 3 trial. Lancet Oncol. 2022;23(2):234-47.

3. Shitara K, Ajani JA, Moehler M, Garrido M, Gallardo C, Shen L, et al. Nivolumab plus chemotherapy or ipilimumab in gastro-oesophageal cancer. Nature. 2022;603(7903):942-8.

4. Lorenzen S, Thuss-Patience PC, Riera Knorrenschild J, Goekkurt E, Dechow TN, Hofheinz R-D, et al. FOLFOX versus FOLFOX plus nivolumab and ipilimumab administered in parallel or sequentially versus FLOT plus nivolumab administered in parallel in patients with previously untreated advanced or metastatic adenocarcinoma of the stomach or gastroesophageal junction: A randomized phase 2 trial of the AIO. Journal of Clinical Oncology. 2022;40(16_suppl):4043-.

5. Shitara K, Van Cutsem E, Bang YJ, Fuchs C, Wyrwicz L, Lee KW, et al. Efficacy and Safety of Pembrolizumab or Pembrolizumab Plus Chemotherapy vs Chemotherapy Alone for Patients With First-line, Advanced Gastric Cancer: The KEYNOTE-062 Phase 3 Randomized Clinical Trial. JAMA Oncol. 2020;6(10):1571-80.

6. Rha SY, Oh DY, Yanez P, Bai Y, Ryu MH, Lee J, et al. Pembrolizumab plus chemotherapy versus placebo plus chemotherapy for HER2-negative advanced gastric cancer (KEYNOTE-859): a multicentre, randomised, double-blind, phase 3 trial. The Lancet Oncology. 2023;24(11):1181-95.

7. Xu J, Jiang H, Pan Y, Gu K, Cang S, Han L, et al. Sintilimab Plus Chemotherapy for Unresectable Gastric or Gastroesophageal Junction Cancer: The ORIENT-16 Randomized Clinical Trial. JAMA. 2023;330(21):2064-74.

8. Zhang X, Wang J, Wang G, Zhang Y, Fan Q, Chuangxin L, et al. LBA79 GEMSTONE-303: Prespecified progression-free survival (PFS) and overall survival (OS) final analyses of a phase III study of sugemalimab plus chemotherapy vs placebo plus chemotherapy in treatment-naive advanced gastric or gastroesophageal junction (G/GEJ) adenocarcinoma. Annals of Oncology. 2023;34(Supplement 2):S1319.

9. Shah MA, Shitara K, Ajani JA, Bang Y-J, Enzinger P, Ilson D, et al. Zolbetuximab plus CAPOX in CLDN18.2-positive gastric or gastroesophageal junction adenocarcinoma: the randomized, phase 3 GLOW trial. Nature Medicine. 2023;29(8):2133-41.

10. Shitara K, Lordick F, Bang YJ, Enzinger P, Ilson D, Shah MA, et al. Zolbetuximab plus mFOLFOX6 in patients with CLDN18.2-positive, HER2-negative, untreated, locally advanced unresectable or metastatic gastric or gastro-oesophageal junction adenocarcinoma (SPOTLIGHT): a multicentre, randomised, double-blind, phase 3 trial. The Lancet. 2023;401(10389):1655-68.

11. Sahin U, Tureci O, Manikhas G, Lordick F, Rusyn A, Vynnychenko I, et al. FAST: a randomised phase II study of zolbetuximab (IMAB362) plus EOX versus EOX alone for first-line treatment of advanced CLDN18.2-positive gastric and gastro-oesophageal adenocarcinoma. Ann Oncol. 2021;32(5):609-19.

12. Wainberg ZA, Enzinger PC, Kang YK, Qin S, Yamaguchi K, Kim IH, et al. Bemarituzumab in patients with FGFR2b-selected gastric or gastro-oesophageal junction adenocarcinoma (FIGHT): a randomised, double-blind, placebo-controlled, phase 2 study. The Lancet Oncology. 2022;23(11):1430-40.

13. FDA. FDA Briefing Document: Immune checkpoint inhibitors in patients with metastatic or unresectable HER2-negative gastric adenocarcinoma. Oncology Advisory Committee Meeting September 26, 2024 Available at: https://www.fda.gov/media/182138/download.
