# Supplementary material for: Comparative efficacy and safety of tislelizumab and other programmed cell death protein 1 inhibitors in first-line treatment of advanced gastroesophageal cancers: a systematic review and network meta-analysis
Source: Gastric Cancer. 2025 Oct 4;28(6):1021–32. doi: 10.1007/s10120-025-01660-4 (PMC12630173; doi:10.1007/s10120-025-01660-4)
Supplement: Supplementary file 3 — Supplementary file3 (DOCX 111 KB) [file 10120_2025_1660_MOESM3_ESM.docx]

# Supplementary material

Online Resource #3 for Comparative Efficacy and Safety of Tislelizumab and other Programmed Cell Death Protein 1 Inhibitors in First-line Treatment of Advanced Gastroesophageal Cancers: A Systematic Review and Network Meta-Analysis

Jaffer A. Ajani^1^ • Maria Alsina ^2^ • Markus Moehler^3^ • Keun-Wook Lee^4^ • Wenxi Tang^5^ • Jason Steenkamp^6^ • Emily Prentiss^6^ • Kaijun Wang^5^ • Becky Hooper^6^ • Lin Zhan^5^

^1^ Department of Gastrointestinal Medical Oncology, Division of Cancer Medicine, The University of Texas MD Anderson Cancer Center, Houston, TX, USA

^2^ Medical Oncology Department, Unidad de Oncología Médica Traslacional, Hospital Universitario de Navarra, Navarrabiomed – IdiSNA, Pamplona, Spain

^3^ Gastrointestinal Oncology, Johannes Gutenberg-University Clinic, Mainz, Germany

^4^ Department of Internal Medicine, Seoul National University College of Medicine, Seoul National University Bundang Hospital, Seongnam, Republic of Korea

^5^BeOne Medicines, Ltd. San Carlos, CA, USA

^6^Value & Evidence Services, EVERSANA, Burlington, ON, Canada

*** Correspondence:**Jaffer A. Ajani
[jajani@mdanderson.org](mailto:jajani@mdanderson.org)

713-792-2828

## Online Resource 3

## SUCRA and P-Best Results for Base Case ITT Analyses

Table S1: Summary of SUCRA values from the fixed-effects NMA for OS

| **Treatment Arm** | **SUCRA (%)** | **Probability Best (%)** |
| --- | --- | --- |
| PEM + CT | 72 | 40 |
| TIS + CT | 69 | 40 |
| NIV + CT | 58 | 20 |
| (PBO) + CT | 0 | 0 |

Abbreviations: *CT* chemotherapy; *NIV* nivolumab; *NMA* network meta-analysis; *OS* overall survival; *PBO* placebo; *PEM* pembrolizumab; *SUCRA* Surface Area Under the Cumulative Ranking Curve; *TIS* tislelizumab.

Table S2: Summary of SUCRA values from the fixed-effects NMA for PFS

| **Treatment Arm** | **SUCRA (%)** | **Probability Best (%)** |
| --- | --- | --- |
| NIV + CT | 73 | 42 |
| TIS + CT | 64 | 33 |
| PEM + CT | 63 | 25 |
| (PBO) + CT | 0 | 0 |

Abbreviations: *CT* chemotherapy; *NIV* nivolumab; *NMA* network meta-analysis; *PBO* placebo; *PEM* pembrolizumab; *PFS* progression-free survival; *SUCRA* Surface Area Under the Cumulative Ranking Curve; *TIS* tislelizumab.

Table S3: Summary of SUCRA values from the fixed-effects NMA for ORR

| **Treatment Arm** | **SUCRA (%)** | **Probability Best (%)** |
| --- | --- | --- |
| NIV + CT | 84 | 60 |
| PEM + CT | 71 | 31 |
| TIS + CT | 44 | 9 |
| (PBO) + CT | 1 | 0 |

Abbreviations: *CT* chemotherapy; *NIV* nivolumab; *NMA* network meta-analysis; *PBO* placebo; *PEM* pembrolizumab; *SUCRA* Surface Area Under the Cumulative Ranking Curve; *TIS* tislelizumab; *TRAE* treatment-related adverse event.

Table S4: Summary of SUCRA values from the fixed-effects NMA for Grade ≥3 TRAE

| **Treatment Arm** | **SUCRA (%)** | **Probability Best (%)** |
| --- | --- | --- |
| (PBO) + CT | 96 | 89 |
| TIS + CT | 64 | 11 |
| PEM + CT | 38 | 0 |
| NIV + CT | 1 | 0 |

Abbreviations: *CT* chemotherapy; *NIV* nivolumab; *NMA* network meta-analysis; *PBO* placebo; *PEM* pembrolizumab; *SUCRA* Surface Area Under the Cumulative Ranking Curve; *TIS* tislelizumab; *TRAE* treatment-related adverse event.

## Additional Subgroup Results

Fig. S1 Fixed-effects League Table for OS in PD-L1 Positive Patients (TAP score ≥ 1%, CPS ≥ 1)

| NIV + CT |  |  |  |
| --- | --- | --- | --- |
| 0.98  (0.84–1.14) | PEM + CT |  |  |
| 0.97  (0.80–1.17) | 0.99  (0.83–1.19) | TIS + CT |  |
| **0.76**  **(0.67–0.85)** | **0.77**  **(0.70–0.86)** | **0.78**  **(0.67–0.90)** | (PBO) + CT |

Note: Reported as HR [95% CrI], HR < 1 implies that column is better than row. Pink squares are statistically significant. The treatment with the most favorable estimate is positioned at the top left corner; the second, third, and fourth most favorable treatments are shown in descending order to the lower right.

RATIONALE-305 data are reflective of the TAP scoring method, CheckMate 649, KEYNOTE-062, and KEYNOTE-859 data are reflective of the CPS method, ATTRACTION-4 part 2 methods are not specified, but assumed to be CPS.​

Stratified HRs were provided for RATIONALE-305 (stratified Cox proportional hazard model including treatment as a covariate stratified by geographic region and peritoneal metastasis), and KEYNOTE-859, and KEYNOTE-062 (both using a stratified Cox proportional hazards model).

Abbreviations: *CrI* credible interval; *CPS* combined positive score; *CT* chemotherapy; *HR* hazard ratio; *NIV* nivolumab; *OS* overall survival; *PBO* placebo; *PD-L1* programmed death-ligand-1; *PEM* pembrolizumab; *TAP* tumor area positivity; *TIS* tislelizumab.

Table S5: Summary of SUCRA values from the fixed-effects NMA for OS in PD-L1 Positive Patients (TAP score ≥ 1%, CPS ≥ 1)

| **Treatment Arm** | **SUCRA (%)** | **Probability Best (%)** |
| --- | --- | --- |
| NIV + CT | 74 | 45 |
| PEM + CT | 64 | 26 |
| TIS + CT | 62 | 29 |
| (PBO) + CT | 0 | 0 |

Abbreviations: *CT* chemotherapy; *NIV* nivolumab; *NMA* network meta-analysis; *OS* overall survival; *PBO* placebo; *PEM* pembrolizumab; *PD-1* programmed cell death-1; *PD-L1* programmed cell death ligand-1; *SUCRA* Surface Area Under the Cumulative Ranking Curve; *TIS* tislelizumab.

Fig. S2 Fixed-effects League Table for OS in PD-L1 Positive Patients (TAP score ≥ 5%, CPS ≥ 5)

| NIV + CT |  |  |  |
| --- | --- | --- | --- |
| 1.00  (0.81–1.23) | PEM + CT |  |  |
| 0.99  (0.77–1.26) | 0.99  (0.77–1.27) | TIS + CT |  |
| **0.70**  **(0.61–0.81)** | **0.70**  **(0.60–0.82)** | **0.71**  **(0.58–0.87)** | (PBO) + CT |

Note: Reported as HR [95% CrI], HR < 1 implies that column is better than row. Pink squares are statistically significant. The treatment with the most favorable estimate is positioned at the top left corner; the second, third, and fourth most favorable treatments are shown in descending order to the lower right.

RATIONALE-305 data are reflective of the TAP scoring method, and CheckMate 649 and KEYNOTE-859 data are reflective of the CPS method.​

Stratified HRs were provided for RATIONALE-305 (stratified Cox proportional hazard model including treatment as a covariate stratified by geographic region and peritoneal metastasis) and CheckMate 649 (stratified Cox proportional hazards regression model, with the randomization factors as the stratification factors and treatment group as a single covariate). Unstratified HRs for KEYNOTE-859 were extracted from the FDA Briefing Document.

Abbreviations: *CrI* credible interval; *CT* chemotherapy; *HR* hazard ratio; *NIV* nivolumab; *PBO* placebo; *PEM* pembrolizumab; *TIS* tislelizumab.

Table S6: Summary of SUCRA values from the fixed-effects NMA for OS in PD-L1 Positive Patients (TAP score ≥ 5%, CPS ≥ 5)

| **Treatment Arm** | **SUCRA (%)** | **Probability Best (%)** |
| --- | --- | --- |
| PEM + CT | 68 | 33 |
| NIV + CT | 68 | 35 |
| TIS + CT | 64 | 32 |
| (PBO) + CT | 0 | 0 |

Abbreviations: *CT* chemotherapy; *NIV* nivolumab; *NMA* network meta-analysis; *OS* overall survival; *PBO* placebo; *PEM* pembrolizumab; *PD-L1* programmed cell death ligand-1; *SUCRA* Surface Area Under the Cumulative Ranking Curve; *TIS* tislelizumab.

Fig. S3 Fixed-effects League Table for PFS in PD-L1 Positive Patients (TAP score ≥ 1%, CPS ≥ 1)

| NIV + CT |  |  |  |
| --- | --- | --- | --- |
| 0.98  (0.83–1.16) | PEM + CT |  |  |
| 0.95  (0.78–1.17) | 0.97  (0.81–1.17) | TIS + CT |  |
| **0.74**  **(0.65–0.85)** | **0.76**  **(0.68–0.84)** | **0.78**  **(0.67–0.91)** | (PBO) + CT |

Note: Reported as HR [95% CrI], HR < 1 implies that column is better than row. Pink squares are statistically significant. The treatment with the most favorable estimate is positioned at the top left corner; the second, third, and fourth most favorable treatments are shown in descending order to the lower right.

RATIONALE-305 data are reflective of the TAP scoring method, CheckMate 649, KEYNOTE-062, and KEYNOTE-859 data are reflective of the CPS method, ATTRACTION-4 part 2 methods are not specified, but assumed to be CPS.​

Stratified HRs were provided for RATIONALE-305 (stratified Cox proportional hazard model including treatment as a covariate stratified by geographic region and peritoneal metastasis), KEYNOTE-859 and KEYNOTE-062 (both using a stratified Cox proportional hazards model), and CheckMate 649 (stratified Cox proportional hazards regression model, with the randomization factors as the stratification factors and treatment group as a single covariate).

Abbreviations: *CrI* credible interval; *CPS* combined positive score; *CT* chemotherapy; *HR* hazard ratio; *NIV* nivolumab; *PBO* placebo; *PD-L1* programmed death-ligand-1; *PEM* pembrolizumab; *PFS* progression-free survival; *TAP* tumor area positivity; *TIS* tislelizumab.

Table S7: Summary of SUCRA values from the fixed-effects NMA for PFS in PD-L1 Positive Patients (TAP score ≥ 1%, CPS ≥ 1)

| **Treatment Arm** | **SUCRA (%)** | **Probability Best (%)** |
| --- | --- | --- |
| NIV + CT | 76 | 48 |
| PEM + CT | 67 | 30 |
| TIS + CT | 57 | 22 |
| (PBO) + CT | 0 | 0 |

Abbreviations: *CT* chemotherapy; *NIV* nivolumab; *NMA* network meta-analysis; *PBO* placebo; *PEM* pembrolizumab; *PD-L1* programmed cell death ligand-1; *PFS* progression-free survival; *SUCRA* Surface Area Under the Cumulative Ranking Curve; *TIS* tislelizumab.

Fig. S4 Fixed-effects League Table for PFS in PD-L1 Positive Patients (TAP score ≥ 5%, CPS ≥ 5)

| TIS + CT |  |  |  |
| --- | --- | --- | --- |
| 0.99  (0.76–1.28) | PEM + CT |  |  |
| 0.97  (0.76–1.25) | 0.99  (0.79–1.23) | NIV + CT |  |
| **0.68**  **(0.56–0.83)** | **0.69**  **(0.58–0.81)** | **0.70**  **(0.60–0.81)** | (PBO) + CT |

Note: Reported as HR [95% CrI], HR < 1 implies that column is better than row. Pink squares are statistically significant. The treatment with the most favorable estimate is positioned at the top left corner; the second, third, and fourth most favorable treatments are shown in descending order to the lower right.

RATIONALE-305 data are reflective of the TAP method, CheckMate 649 and KEYNOTE-859 data are reflective of the CPS method.

Stratified HRs were provided for RATIONALE-305 (stratified Cox proportional hazard model including treatment as a covariate stratified by geographic region and peritoneal metastasis) and CheckMate 649 (stratified Cox proportional hazards regression model, with the randomization factors as the stratification factors and treatment group as a single covariate). Unstratified HRs for KEYNOTE-859 were extracted from the FDA Briefing Document.

Abbreviations: *CrI* credible interval; *CT* chemotherapy; *HR* hazard ratio; *Niv* nivolumab; *PBO* placebo; *Pem* pembrolizumab; *Tis* tislelizumab.

Table S8: Summary of SUCRA values from the fixed-effects NMA for PFS in PD-L1 Positive Patients (TAP score ≥ 5%, CPS ≥ 5)

| **Treatment Arm** | **SUCRA (%)** | **Probability Best (%)** |
| --- | --- | --- |
| TIS + CT | 71 | 42 |
| PEM + CT | 67 | 33 |
| NIV + CT | 62 | 25 |
| (PBO) + CT | 0 | 0 |

Abbreviations: *CT* chemotherapy; *NIV* nivolumab; *NMA* network meta-analysis; *PBO* placebo; *PEM* pembrolizumab; *PD-L1* programmed cell death ligand-1; *PFS* progression-free survival; *SUCRA* Surface Area Under the Cumulative Ranking Curve; *TIS* tislelizumab.

Fig. S5 Fixed-effects League Table for OS in ROW (non-Asian) Regions

| TIS + CT |  |  |  |
| --- | --- | --- | --- |
| 0.88  (0.64–1.22) | PEM + CT |  |  |
| 0.88  (0.64–1.20) | 1.00  (0.81–1.23) | NIV + CT |  |
| **0.72**  **(0.54–0.96)** | **0.82**  **(0.69–0.96)** | **0.82**  **(0.72–0.94)** | (PBO) + CT |

Note: Reported as HR [95% CrI], HR < 1 implies that column is better than row. Pink squares are statistically significant. The treatment with the most favorable estimate is positioned at the top left corner; the second, third, and fourth most favorable treatments are shown in descending order to the lower right.

Tislelizumab HR was stratified by PD-L1 status (5% cutoff) and presence of peritoneal metastases. All other inputs were not stratified.

Abbreviations: *CrI* credible interval; *CT* chemotherapy; *HR* hazard ratio; *NIV* nivolumab; *OS* overall survival; *PBO* placebo; *PEM* pembrolizumab; *TIS* tislelizumab.

Table S9: Summary of SUCRA values from the fixed-effects NMA for OS in ROW (non-Asian) Regions

| **Treatment Arm** | **SUCRA (%)** | **Probability Best (%)** |
| --- | --- | --- |
| TIS + CT | 85 | 71 |
| PEM + CT | 58 | 16 |
| NIV + CT | 56 | 13 |
| (PBO) + CT | 1 | 0 |

Abbreviations: *CT* chemotherapy; *NIV* nivolumab; *NMA* network meta-analysis; *OS* overall survival; *PBO* placebo; *PEM* pembrolizumab; *SUCRA* Surface Area Under the Cumulative Ranking Curve; *TIS* tislelizumab.

Fig. S6 Fixed-effects League Table for OS in Asian Regions

| PEM + CT |  |  |  |
| --- | --- | --- | --- |
| 0.95  (0.71–1.28) | NIV + CT |  |  |
| 0.87  (0.68–1.11) | 0.91  (0.69–1.22) | TIS + CT |  |
| **0.72**  **(0.61–0.87)** | **0.76**  **(0.60–0.96)** | **0.83**  **(0.71–0.98)** | (PBO) + CT |

Note: Reported as HR [95% CrI], HR < 1 implies that column is better than row. Pink squares are statistically significant. The treatment with the most favorable estimate is positioned at the top left corner; the second, third, and fourth most favorable treatments are shown in descending order to the lower right.

Tislelizumab HR was stratified by PD-L1 status (5% cutoff) and presence of peritoneal metastases. All other inputs were not stratified.

Abbreviations: *CrI* credible interval; *CT* chemotherapy; *HR* hazard ratio; *NIV* nivolumab; *OS* overall survival; *PBO* placebo; *PEM* pembrolizumab; *TIS* tislelizumab.

Table S10: Summary of SUCRA values from the fixed-effects NMA for OS in Asian Regions

| **Treatment Arm** | **SUCRA (%)** | **Probability Best (%)** |
| --- | --- | --- |
| PEM + CT | 83 | 58 |
| NIV + CT | 70 | 35 |
| TIS + CT | 46 | 7 |
| (PBO) + CT | 1 | 0 |

Abbreviations: *CT* chemotherapy; *NIV* nivolumab; *NMA* network meta-analysis; *OS* overall survival; *PBO* placebo; *PEM* pembrolizumab; *SUCRA* Surface Area Under the Cumulative Ranking Curve; *TIS* tislelizumab.

Fig. S7 Fixed-effects League Table for PFS in ROW (non-Asian) Regions

| PEM + CT |  |  |
| --- | --- | --- |
| 0.93  (0.65–1.34) | TIS + CT |  |
| **0.79**  **(0.63–0.99)** | 0.85  (0.64–1.13) | (PBO) + CT |

Note: Reported as HR [95% CrI], HR < 1 implies that column is better than row. Pink squares are statistically significant. The treatment with the most favorable estimate is positioned at the top left corner; the second, and third most favorable treatments are shown in descending order to the lower right.

Tislelizumab HR was stratified by PD-L1 status (5% cutoff) and presence of peritoneal metastases. Pembrolizumab HR was unstratified.

Abbreviations: *CrI* credible interval; *CT* chemotherapy; *HR* hazard ratio; *NMA* network meta-analysis; *PBO* placebo; *PEM* pembrolizumab; *PFS* progression-free survival; *RCT* randomized controlled trial; *TIS* tislelizumab.

Table S11: Summary of SUCRA values from the fixed-effects NMA for PFS in ROW (non-Asia) Regions

| **Treatment Arm** | **SUCRA (%)** | **Probability Best (%)** |
| --- | --- | --- |
| PEM + CT | 81 | 65 |
| TIS + CT | 61 | 35 |
| (PBO) + CT | 8 | 0 |

Abbreviations: *CT* chemotherapy; *NMA* network meta-analysis; *PBO* placebo; *PEM* pembrolizumab; *PFS* progression-free survival; *SUCRA* Surface Area Under the Cumulative Ranking Curve; *TIS* tislelizumab.

Fig. S8 Fixed-effects League Table for PFS in Asian Regions

| PEM + CT |  |  |
| --- | --- | --- |
| 0.95  (0.73–1.24) | TIS + CT |  |
| **0.72**  **(0.58–0.89)** | **0.76**  **(0.64–0.89)** | (PBO) + CT |

Note: Reported as HR [95% CrI], HR < 1 implies that column is better than row. Pink squares are statistically significant. The treatment with the most favorable estimate is positioned at the top left corner; the second, and third most favorable treatments are shown in descending order to the lower right.

Tislelizumab HR was stratified by PD-L1 status (5% cutoff) and presence of peritoneal metastases. Pembrolizumab HR was not stratified.

Abbreviations: *CrI* credible interval; *CT* chemotherapy; *HR* hazard ratio; *NMA* network meta-analysis; *PBO* placebo; *PEM* pembrolizumab; *PFS* progression-free survival; *RCT* randomized controlled trial; *TIS* tislelizumab.

Table S12: Summary of SUCRA values from the fixed-effects NMA for PFS in Asian Regions

| **Treatment Arm** | **SUCRA (%)** | **Probability Best (%)** |
| --- | --- | --- |
| PEM + CT | 82 | 65 |
| TIS + CT | 68 | 35 |
| (PBO) + CT | 0 | 0 |

Abbreviations: *CT* chemotherapy; *NMA* network meta-analysis; *PBO* placebo; *PEM* pembrolizumab; *PFS* progression-free survival; *SUCRA* Surface Area Under the Cumulative Ranking Curve; *TIS* tislelizumab.

Fig. S9 Fixed-effects League Table for OS in Patients with Gastric Primary Tumor Location

| PEM + CT |  |  |  |
| --- | --- | --- | --- |
| 1.00  (0.85–1.17) | NIV + CT |  |  |
| 0.94  (0.77–1.14) | 0.94  (0.77–1.14) | TIS + CT |  |
| **0.78**  **(0.70–0.87)** | **0.78**  **(0.70–0.87)** | **0.83**  **(0.71–0.97)** | (PBO) + CT |

Note: Reported as HR [95% CrI], HR < 1 implies that column is better than row. Pink squares are statistically significant. The treatment with the most favorable estimate is positioned at the top left corner; the second, and third most favorable treatments are shown in descending order to the lower right.

All hazard ratios were unstratified except for RATIONALE-305, which was stratified by geographic region (East Asia vs. rest of world), PD-L1 status (5% cutoff), and presence of peritoneal metastases.

Abbreviations: *CT* chemotherapy; *NIV* nivolumab; *OS* overall survival; *PBO* placebo; *PEM* pembrolizumab; *TIS* tislelizumab.

Table S13: Summary of SUCRA values from the fixed-effects NMA for OS in patients with gastric primary tumor location

| **Treatment Arm** | **SUCRA (%)** | **Probability Best (%)** |
| --- | --- | --- |
| PEM + CT | 75 | 43 |
| NIV + CT | 74 | 42 |
| TIS + CT | 51 | 16 |
| (PBO) + CT | 0 | 0 |

Abbreviations: *CT* chemotherapy; *NIV* nivolumab; *NMA* network meta-analysis; *OS* overall survival; *PBO* placebo; *PEM* pembrolizumab; *SUCRA* Surface Area Under the Cumulative Ranking Curve; *TIS* tislelizumab.

Fig. S10 Fixed-effects League Table for OS in Patients with Gastroesophageal Junction Primary Tumor Location

| TIS + CT |  |  |  |
| --- | --- | --- | --- |
| 0.93  (0.63–1.38) | PEM + CT |  |  |
| 0.82  (0.54–1.25) | 0.89  (0.65–1.22) | NIV + CT |  |
| **0.75**  **(0.53–1.05)** | **0.80**  **(0.66–0.98)** | 0.91  (0.71–1.16) | (PBO) + CT |

Note: Reported as HR [95% CrI], HR < 1 implies that column is better than row. Pink squares are statistically significant. The treatment with the most favorable estimate is positioned at the top left corner; the second, and third most favorable treatments are shown in descending order to the lower right.

All hazard ratios were unstratified except for RATIONALE-305, which was stratified by geographic region (East Asia vs. rest of world), PD-L1 status (5% cutoff), and presence of peritoneal metastases.

Abbreviations: *CT* chemotherapy; *NIV* nivolumab; *OS* overall survival; *PBO* placebo; *PEM* pembrolizumab; *TIS* tislelizumab.

Table S14: Summary of SUCRA values from the fixed-effects NMA for OS in patients with gastroesophageal junction primary tumor location

| **Treatment Arm** | **SUCRA (%)** | **Probability Best (%)** |
| --- | --- | --- |
| TIS + CT | 80 | 60 |
| PEM + CT | 71 | 31 |
| NIV + CT | 40 | 8 |
| (PBO) + CT | 9 | 0 |

Abbreviations: *CT* chemotherapy; *NIV* nivolumab; *NMA* network meta-analysis; *OS* overall survival; *PBO* placebo; *PEM* pembrolizumab; *SUCRA* Surface Area Under the Cumulative Ranking Curve; *TIS* tislelizumab.

Fig. S11 Fixed-effects League Table for OS in Patients who Received CAPOX

| PEM + CT |  |  |  |
| --- | --- | --- | --- |
| 0.96  (0.80–1.15) | TIS + CT |  |  |
| 0.93  (0.78–1.11) | 0.97  (0.79–1.19) | NIV + CT |  |
| **0.76**  **(0.69–0.85)** | **0.79**  **(0.68–0.92)** | **0.82**  **(0.71–0.94)** | (PBO) + CT |

Note: Reported as HR [95% CrI], HR < 1 implies that column is better than row. Pink squares are statistically significant. The treatment with the most favorable estimate is positioned at the top left corner; the second, and third most favorable treatments are shown in descending order to the lower right.

All hazard ratios were unstratified except for RATIONALE-305, which was stratified by geographic region (East Asia vs. ROW), PD-L1 (5% cutoff), and presence of peritoneal metastases.

Abbreviations: *CT* chemotherapy; *NIV* nivolumab; *OS* overall survival; *PBO* placebo; *PEM* pembrolizumab; *TIS* tislelizumab.

Table S15: Summary of SUCRA values from the fixed-effects NMA for OS in patients who received CAPOX

| **Treatment Arm** | **SUCRA (%)** | **Probability Best (%)** |
| --- | --- | --- |
| PEM + CT | 82 | 56 |
| TIS + CT | 64 | 28 |
| NIV + CT | 54 | 16 |
| (PBO) + CT | 0 | 0 |

Abbreviations: *CT* chemotherapy; *NIV* nivolumab; *NMA* network meta-analysis; *OS* overall survival; *PBO* placebo; *PEM* pembrolizumab; *SUCRA* Surface Area Under the Cumulative Ranking Curve; *TIS* tislelizumab.

Fig. S12 Fixed-effects League Table for ORR in PD-L1 Positive Patients (TAP score ≥ 5%, CPS ≥ 5)

| NIV + CT |  |  |  |
| --- | --- | --- | --- |
| 1.20  (0.82–1.76) | PEM + CT |  |  |
| 1.29  (0.84–1.97) | 1.07  (0.69–1.67) | TIS + CT |  |
| 1.84  (1.42–2.38) | 1.53  (1.16–2.04) | 1.43  (1.02–2.00) | (PBO) + CT |

Note: Reported as OR [95% CrI], OR > 1 implies that column is better than row. Pink squares are statistically significant. The treatment with the most favorable estimate is positioned at the top left corner; the second, and third most favorable treatments are shown in descending order to the lower right.

RATIONALE-305 data are reflective of the TAP method, CheckMate 649 and KEYNOTE-859 data are reflective of the CPS method. Data for KEYNOTE-859 were extracted from the FDA Briefing Document.

Abbreviations: *CT* chemotherapy; *NIV* nivolumab; *ORR* objective response rate; *PBO* placebo; *PD-L1* programmed death ligand-1; *PEM* pembrolizumab; *TAP* Tumor Area Positivity; *TIS* tislelizumab.

Table S16: Summary of SUCRA values from the fixed-effects NMA for ORR in PD-L1 Positive Patients (TAP score ≥ 5%, CPS ≥ 5)

| **Treatment Arm** | SUCRA (%) | Probability Best (%) |
| --- | --- | --- |
| NIV + CT | 90 | 75 |
| PEM + CT | 60 | 15 |
| TIS + CT | 49 | 10 |
| (PBO) + CT | 1 | 0 |

Abbreviations: *CT* chemotherapy; *NIV* nivolumab; *NMA* network meta-analysis; *ORR* objective response rate; *PBO* placebo; *PEM* pembrolizumab; *SUCRA* Surface Area Under the Cumulative Ranking Curve; *TIS* tislelizumab.

Fig. S13 Fixed-effects League Table for OS Sensitivity Analysis using CPS ≥ 5

| NIV + CT |  |  |  |
| --- | --- | --- | --- |
| 1.00  (0.81–1.23) | PEM + CT |  |  |
| 1.00  (0.78–1.28) | 0.99  (0.77–1.29) | TIS + CT |  |
| 0.70  (0.61–0.81) | 0.70  (0.60–0.82) | 0.70  (0.57–0.86) | (PBO) + CT |

Note: Reported as HR [95% CrI], HR < 1 implies that column is better than row. Pink squares are statistically significant. The treatment with the most favorable estimate is positioned at the top left corner; the second, third, and fourth most favorable treatments are shown in descending order to the lower right.

Abbreviations: *CPS* combined positive score; *CT* chemotherapy; *NIV* nivolumab; *OS* overall survival; *PBO* placebo; *PEM* pembrolizumab; *TIS* tislelizumab.

Table S17: Summary of SUCRA values from the fixed-effects NMA for OS Sensitivity Analysis using CPS ≥ 5

| **Treatment Arm** | SUCRA (%) | Probability Best (%) |
| --- | --- | --- |
| NIV + CT | 67 | 32 |
| PEM + CT | 67 | 33 |
| TIS + CT | 66 | 35 |
| (PBO) + CT | 0 | 0 |

Abbreviations: *CPS* combined positive score; *CT* chemotherapy; *NIV* nivolumab; *NMA* network meta-analysis; *OS* overall survival; *PBO* placebo; *PEM* pembrolizumab; *SUCRA* Surface Area Under the Cumulative Ranking Curve; *TIS* tislelizumab.
