# Supplementary material for: Comparative efficacy and safety of tislelizumab and other programmed cell death protein 1 inhibitors in first-line treatment of advanced gastroesophageal cancers: a systematic review and network meta-analysis
Source: Gastric Cancer. 2025 Oct 4;28(6):1021–32. doi: 10.1007/s10120-025-01660-4 (PMC12630173; doi:10.1007/s10120-025-01660-4)
Supplement: Supplementary file 4 — Supplementary file4 (DOCX 325 KB) [file 10120_2025_1660_MOESM4_ESM.docx]

# Supplementary material

Online Resource #4 for Comparative Efficacy and Safety of Tislelizumab and other Programmed Cell Death Protein 1 Inhibitors in First-line Treatment of Advanced Gastroesophageal Cancers: A Systematic Review and Network Meta-Analysis

Jaffer A. Ajani^1^ • Maria Alsina ^2^ • Markus Moehler^3^ • Keun-Wook Lee^4^ • Wenxi Tang^5^ • Jason Steenkamp^6^ • Emily Prentiss^6^ • Kaijun Wang^5^ • Becky Hooper^6^ • Lin Zhan^5^

^1^ Department of Gastrointestinal Medical Oncology, Division of Cancer Medicine, The University of Texas MD Anderson Cancer Center, Houston, TX, USA

^2^ Medical Oncology Department, Unidad de Oncología Médica Traslacional, Hospital Universitario de Navarra, Navarrabiomed – IdiSNA, Pamplona, Spain

^3^ Gastrointestinal Oncology, Johannes Gutenberg-University Clinic, Mainz, Germany

^4^ Department of Internal Medicine, Seoul National University College of Medicine, Seoul National University Bundang Hospital, Seongnam, Republic of Korea

^5^BeOne Medicines, Ltd. San Carlos, CA, USA

^6^Value & Evidence Services, EVERSANA, Burlington, ON, Canada

*** Correspondence:**Jaffer A. Ajani
[jajani@mdanderson.org](mailto:jajani@mdanderson.org)

713-792-2828

## Online Resource 4 – Within-Trial Proportional Hazards Assumption Results

Cumulative hazard log plots for investigator-assessed PFS, ITT population (RATIONALE-305)


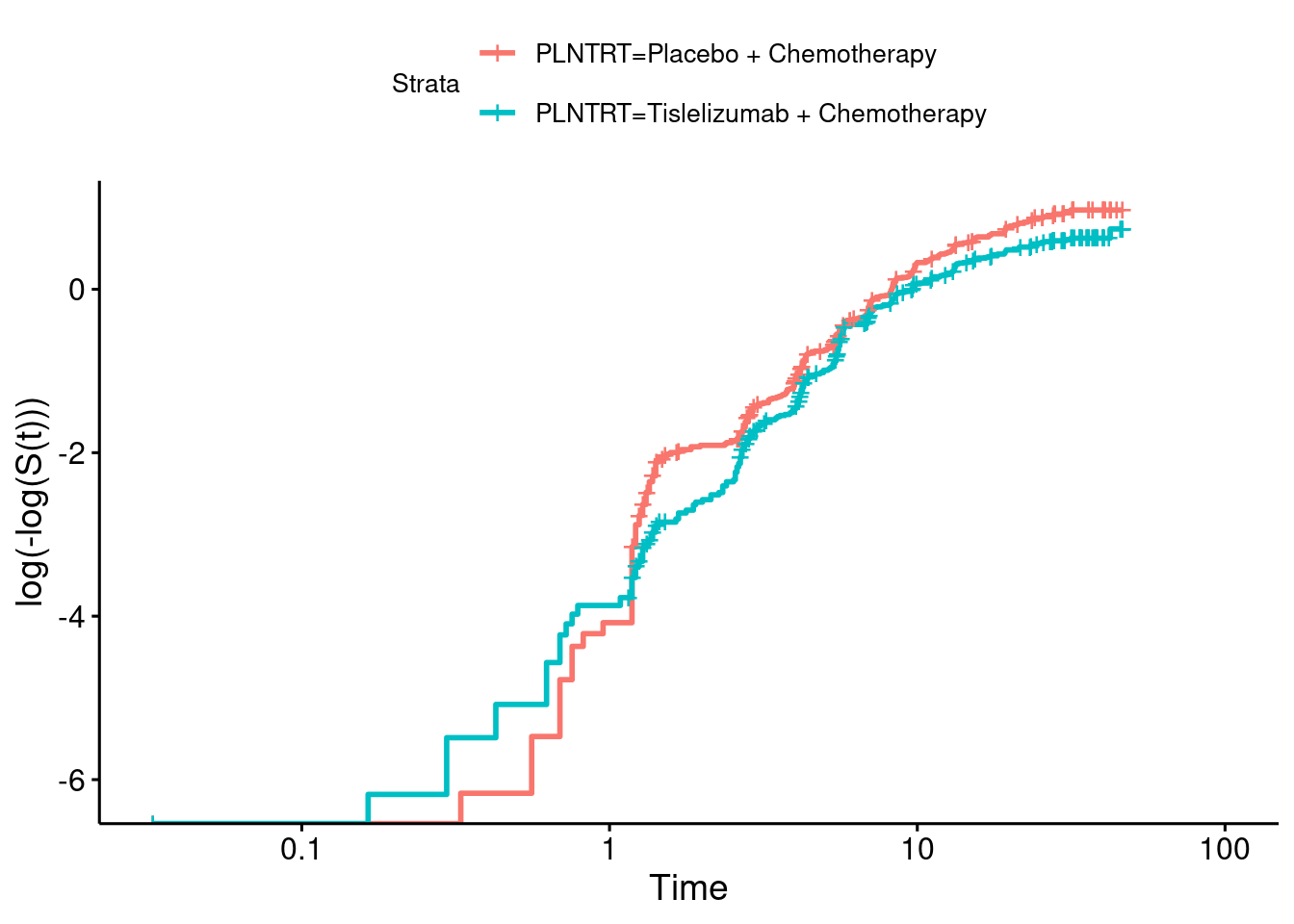


Abbreviations: *ITT* Intent-to-Treat; *PFS* progression-free survival.

Schoenfeld residual plots with Grambsch-Therneau p-values for investigator-assessed PFS, ITT population (RATIONALE-305)


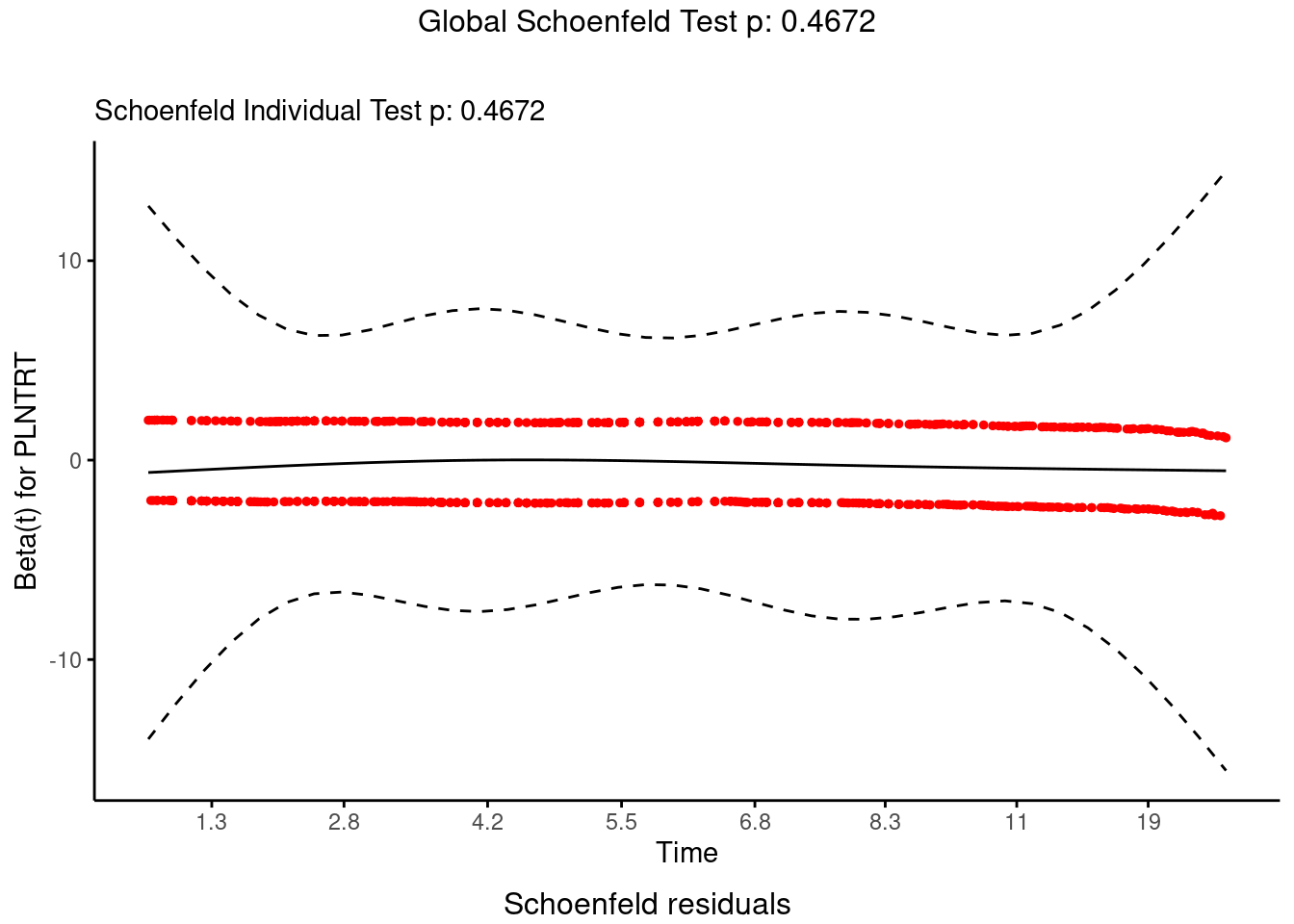


Abbreviations: *ITT* Intent-to-Treat; *PFS* progression-free survival.

Cumulative hazard log plots for OS, ITT population (RATIONALE-305)


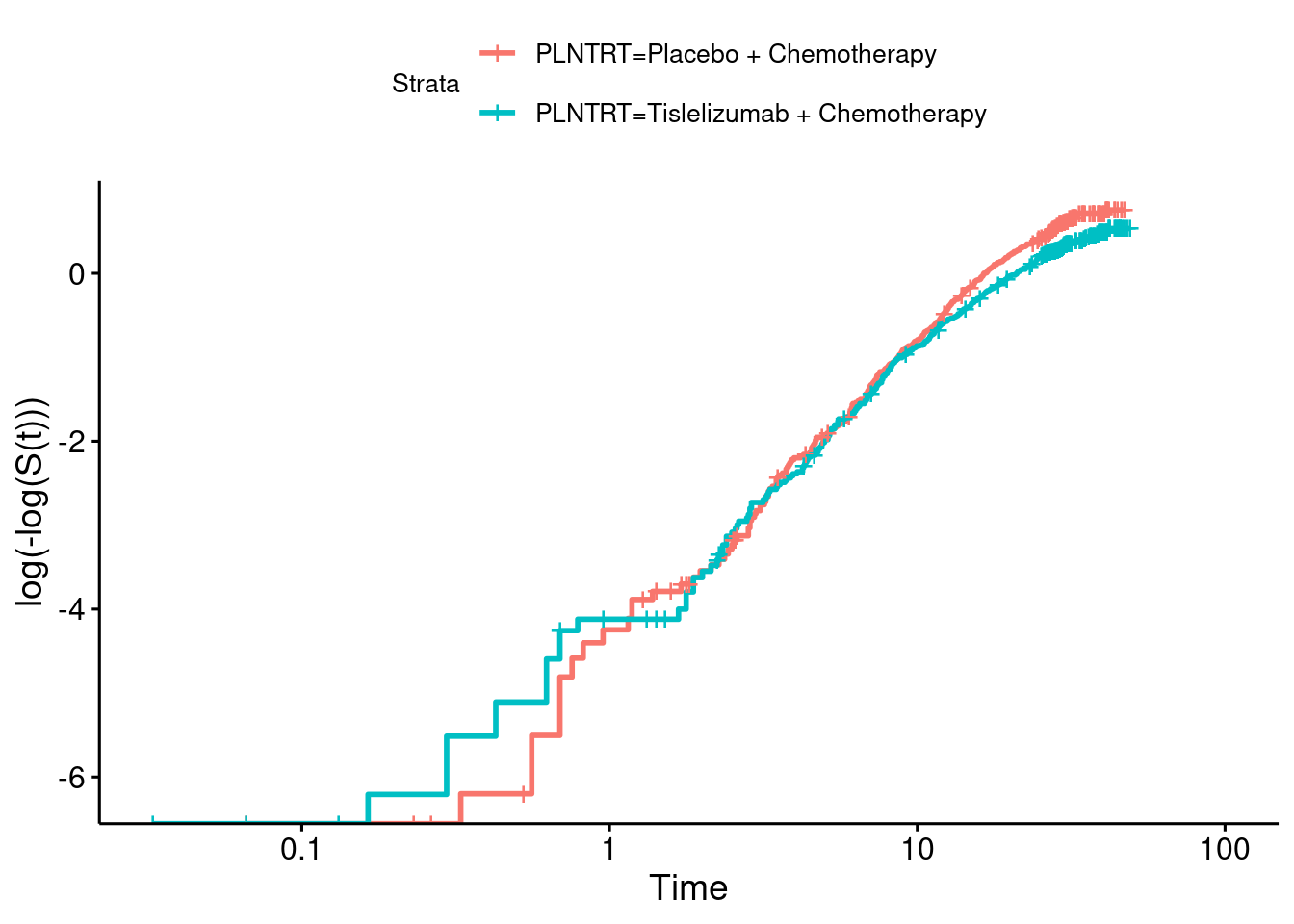


Abbreviations: *ITT* Intent-to-Treat; *OS* overall survival.

Schoenfeld residual plots with Grambsch-Therneau p-values for OS, ITT population (RATIONALE-305)


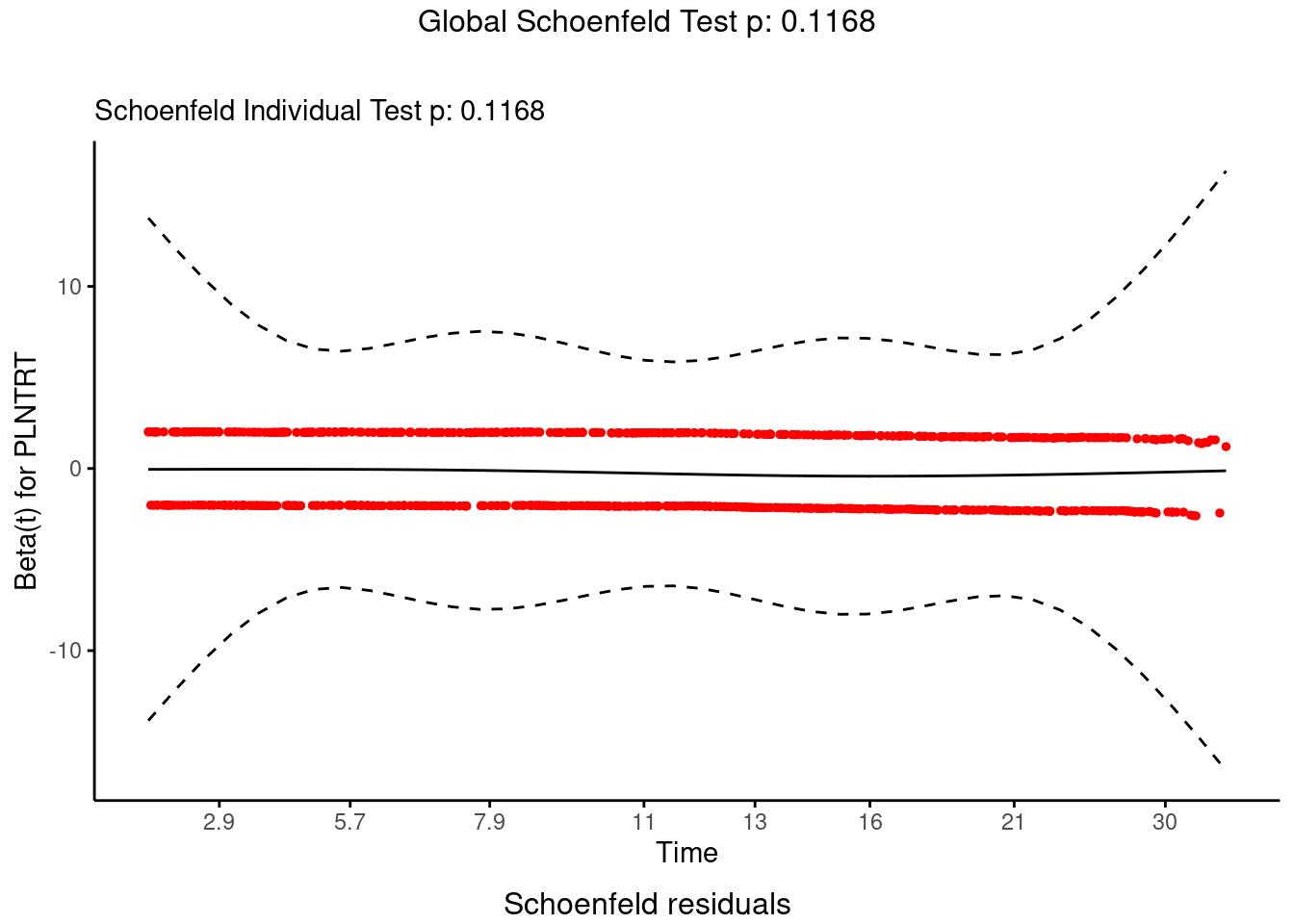


Abbreviations: *ITT* Intent-to-Treat; *OS* overall survival.
